# Diastereoselective auxiliary- and catalyst-controlled intramolecular aza-Michael reaction for the elaboration of enantioenriched 3-substituted isoindolinones. Application to the synthesis of a new pazinaclone analogue

**DOI:** 10.3762/bjoc.14.46

**Published:** 2018-03-09

**Authors:** Romain Sallio, Stéphane Lebrun, Frédéric Capet, Francine Agbossou-Niedercorn, Christophe Michon, Eric Deniau

**Affiliations:** 1 Univ, Lille, CNRS, Centrale Lille, ENSCL, Univ. Artois, UMR 8181-UCCS-Unité de Catalyse et Chimie du Solide, F-59000 Lille, France

**Keywords:** asymmetric organocatalysis, Aza-Michael reaction, phase-transfer catalyst, 3-substituted isoindolinones

## Abstract

A new asymmetric organocatalyzed intramolecular aza-Michael reaction by means of both a chiral auxiliary and a catalyst for stereocontrol is reported for the synthesis of optically active isoindolinones. A selected cinchoninium salt was used as phase-transfer catalyst in combination with a chiral nucleophile, a Michael acceptor and a base to provide 3-substituted isoindolinones in good yields and diastereomeric excesses. This methodology was applied to the asymmetric synthesis of a new pazinaclone analogue which is of interest in the field of benzodiazepine-receptor agonists.

## Introduction

**Figure 1:**
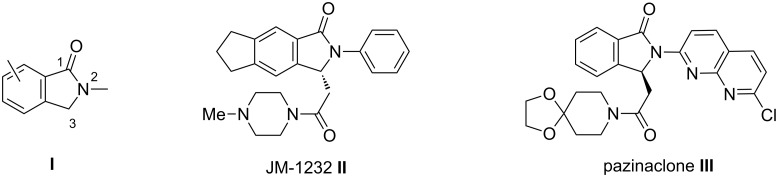
Examples of synthetic pharmacologically active chiral 3-substituted isoindolinones.

Isoindolinones **I** (Figure 1), e.g., 2,3-dihydro-1*H*-isoindol-1-ones, also called phthalimidines are bicyclic lactams whose molecular structure is the basis of a wide range of alkaloids and biologically active compounds [1–11]. Among the latter, optically pure compounds functionalized at C-3 by acetamido groups play an important role as key targets for the pharmaceutical industry. For example, substituted 3-isoindolinones, such as JM-1232 (**II**) [12–14] and pazinaclone (**III**) [15–16] (Figure 1), have shown sedative-hypnotic activities used for the treatment of anxiety by acting as partial agonists at GABA_A_ (γ-aminobutyric acid type A) benzodiazepine receptors [17]. All these studies have highlighted a strong correlation between the compound pharmacological activities and the absolute configurations of their stereocenter [14]. Hence, the asymmetric synthesis of functionalized 3-substituted isoindolinones using short, versatile and selective procedures is clearly a topic of current interest.

Two strategies can be applied for the asymmetric synthesis of 3-substituted isoindolinones. First, diastereoselective reactions implying the use of a chiral auxiliary resulted effectively in various optically pure compounds [10,18–20]. Second, enantioselective syntheses of these bicylic lactams were performed by using chiral transition metal- or organocatalysts which control the configuration of the trisubstituted carbon stereocenter alpha to the nitrogen [10,20–34]. Though various metal or organic catalysts were used to promote the aza-Michael reaction in different syntheses for the creation of nitrogen–carbon bonds, phase-transfer catalysts were less studied (see reviews [35–38]) in intermolecular [39–43] and intramolecular [44–46] sequences. Among the latter, a short regio- and stereoselective organocatalyzed intramolecular aza-Michael reaction was reported by us for the asymmetric synthesis of several isoindolinones [20,34]. Indeed, we noticed along our studies some intramolecular aza-Michael reactions were effectively catalysed by cinchoninium phase-transfer catalysts (PCT) affording the targeted 3-substituted isoindolinones with promising enantioselectivities (up to 91%) [20]. However, high enantioselectivities were reached only for specific substitution patterns on the amide nitrogen atom and to a lesser extent on the Michael acceptor. In order to overcome these limitations, we decided to incorporate a chiral auxiliary in our substrates combined with a proper chiral phase-transfer organocatalyst to operate an efficient stereocontrol. To the best of our knowledge such approach involving a double auxiliary and catalyst stereocontrol was never applied before to asymmetric synthesis of enantioenriched isoindolinones.

## Results and Discussion

### Retrosynthetic analysis

**Scheme 1:**
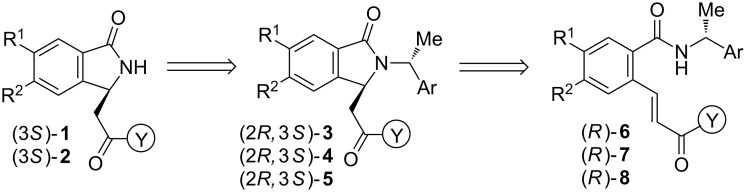
Retrosynthetic analysis of NH free chiral 3-substituted isoindolinones (3*S*)-**1** and (3*S*)-**2**.

From a retrosynthetic point of view, (3*S*)-NH free 3-substituted isoindolinones **1** and **2** could be obtained in high enantioselectivities from the intermediates (2*R*,3*S*)-**3**–**5** after removal of the chiral auxiliary (Scheme 1). (2*R*,3*S*)-bicyclic lactams **3**–**5** could be prepared by the asymmetric intramolecular organo-catalyzed aza-Michael reaction of (*R*)-benzamides **6**–**8** bearing an acrylamide group at the *ortho-*position of the benzene ring.

### Synthesis of parent chiral benzamides **6**–**8**

**Scheme 2:**
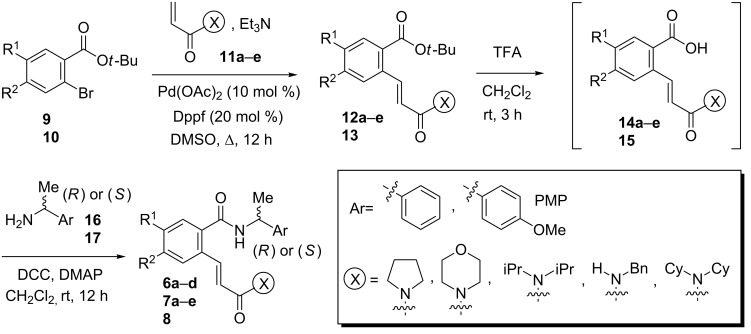
Synthesis of parent benzamides **6**–**8**.

**Figure 2:**
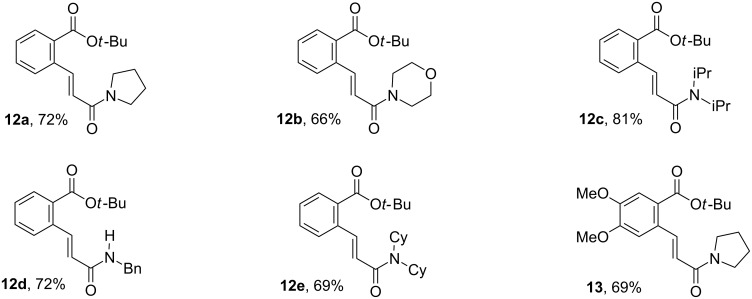
Esters **12a**–**e**, **13** prepared, isolated yield.

**Figure 3:**
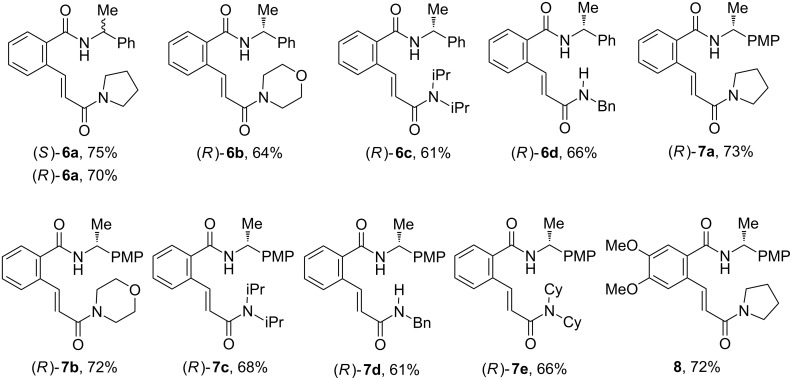
Benzamides **6a**–**d**, **7a**–**e**, **8** prepared, isolated yield.

The use of a stereoselective chiral auxiliary which could be incorporated and removed easily without racemization was crucial for the success of our strategy. These requirements prompted us to incorporate α-methylbenzylamine-type chiral auxiliaries, which have been extensively used by Davies et al. to gain access to a wide range of chiral N-heterocycles via intermolecular aza-Michael reactions [34,47–51]. The starting unsaturated benzoic acids **14a**–**e** and **15** were readily prepared via a two steps sequence involving first a palladium-catalyzed Heck cross coupling between 2-bromobenzoic *tert*-butyl esters **9** and **10** with acrylamides **11a**–**e** (69–72% isolated yields, Scheme 2, Figure 2). The subsequent removal of the *t*-butyl group in esters **12a**–**e** and **13** (Figure 2) was then achieved by treatment with trifluoroacetic acid to provide in-situ the corresponding benzoic acids **14a**–**e** and **15**. The direct coupling of these functionalized carboxylic acids with chiral benzylic primary amines, (*R*) or (*S*)-**16** (NH_2_-CH(Me)Ph) and (*R*)-**17** (NH_2_CH(Me)*p*-MeO-C_6_H_4_), afforded the required parent amides **6a**–**d, 7a**–**e** and **8** in 61–75% isolated yields after work-up (Scheme 2, Figure 3).

### Diastereoselective intramolecular aza-Michael reaction

**Figure 4:**
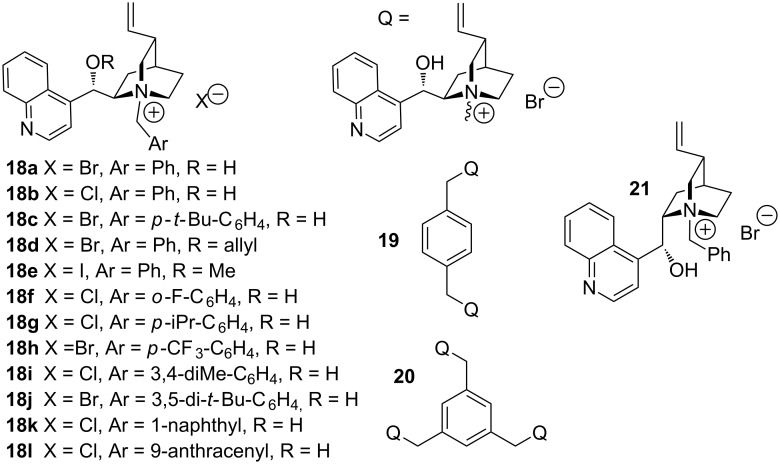
Phase transfer catalysts (PTC) used in this study.

First, the study of the diastereoselective intramolecular aza-Michael reaction of benzamide substrate (*S*)**-6a** allowed us to optimize the reaction conditions (Table 1) and latter to screen various privileged phase-transfer catalysts (Figure 4, Table 2). As some aza-Michael reactions were shown to be performed without the use of any catalyst or additional reagent [52–60], we performed control experiments (Table 1). The reaction of reagent (*S*)-**6a** led to product (*S*)-**3a** solely by using a base like Cs_2_CO_3_ in toluene with a good yield (74%) and a modest diastereomeric excess (37% de, Table 1, entry 1). Increasing the reaction time from 16 h to 36 h led to higher diastereomeric excess but no further improvement was noticed with longer reaction times (Table 1, entries 2 and 3). Such chiral amplification versus time was already found to operate through a retro-aza-Michael reaction [61–62]. Indeed, through an equilibration of aza-Michael and retro-aza-Michael reactions, the minor diastereoisomer of **3a** may lead back to a racemic starting material and subsequently favour the major diastereoisomer (Table 1, entries 1–3). The use of a catalytic amount of base led to product **3a** in a good yield (Table 1, entry 4) but with a loss of diastereoselectivity. Because the optically pure auxiliary and the conjugated ketone were not interacting well, a significant diastereoselectivity could not be obtained and we looked for improvements through the use of an appropriate chiral organocatalyst [63–65]. Indeed, within the same reaction conditions, the use of cinchoninium catalyst **18a** afforded isoindolinone (*S*)-**3a** with higher de (54%), (Table 1, entry 5). We assumed such de increase resulted from a match effect [65–67] of the diastereomeric ion pair formed by the chiral nucleophile, the conjugated ketone and the cinchoninium salt. Hence, in our case, the chirality of the new stereochemical center was shown to be controlled by both Michael acceptor and donor interacting with the chiral ammonium. Surprisingly, the diastereoselectivity initially obtained for **3a** (Table 1, entry 5) was not improved by a decrease of the temperature to −10 °C (Table 1, entry 6) or by the use of polar solvents (Table 1, entries 7 and 8) or of another base (Table 1, entry 9).

**Table 1:** Identification of the optimum reaction conditions for the diastereoselective intramolecular aza-Michael reaction of (*S*)-**6a**.

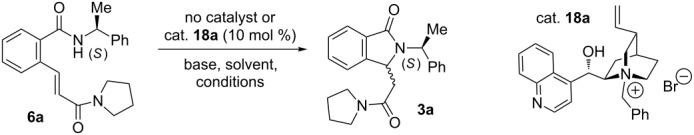						

entry	catalyst	solvent	base (equiv)	conditions	yield (%)^a^	de (%)^b^

1	–	toluene	Cs_2_CO_3_ (1.3)	rt, 16 h	(2*S*)-**3a** (74)	37
2	–	toluene	Cs_2_CO_3_ (1.3)	rt, 24 h	(2*S*)-**3a** (80)	40
3	–	toluene	Cs_2_CO_3_ (1.3)	rt, 36 h	(2*S*)-**3a** (80)	44^c^
4	–	toluene	Cs_2_CO_3_ (0.1)	rt, 36 h	(2*S*)-**3a** (82)	30
5	**18a**	toluene	Cs_2_CO_3_ (1.3)	rt, 36 h	(2*S*)-**3a** (75)	54
6	**18a**	toluene	Cs_2_CO_3_ (1.3)	−10 °C, 36 h	(2*S*)-**3a** (75)	46
7	**18a**	THF	Cs_2_CO_3_ (1.3)	rt, 36 h	(2*S*)-**3a** (73)	40
8	**18a**	CH_2_Cl_2_	Cs_2_CO_3_ (1.3)	rt, 36 h	(2*S*)-**3a** (80)	40
9	**18a**	toluene	Ba(OH)_2_ (1.3)	rt, 36 h	(2*S*)-**3a** (81)	42

^a^After purification. ^b^Determined by HPLC and ^1^H NMR. ^c^No change with longer reaction times.

In order to identify the most active catalyst for the aza-Michael reaction of (*S*)-and (*R*)-**6a**, an array of phase-transfer catalysts was screened (Figure 4, Table 2). By comparing catalysts **18a** and **18b**, a bromide anion was shown to be preferred to a chloride one (Table 2, entries 1 and 2). Catalyst **18c** *para*-substituted with a *tert-*butyl increased significantly the diastereoselectivity of the reaction with 62% de (Table 2, entry 3).

**Table 2:** Identification of the most active catalyst for the diastereoselective intramolecular aza-Michael reaction of (*S*)- and (*R*)-**6a**.

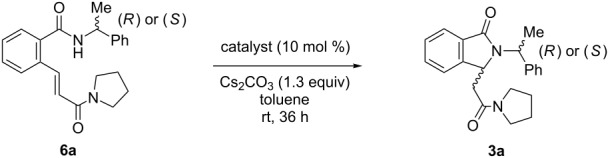				

entry	reagent	catalyst	yield (%)^a^	de (%)^b^

1	(*S*)-**6a**	**18a**	(2*S*)-**3a** (75)	54
2	(*S*)-**6a**	**18b**	(2*S*)-**3a** (73)	44
3	(*S*)-**6a**	**18c**	(2*S*)-**3a** (77)	62
4	(*S*)-**6a**	**18d**	(2*S*)-**3a** (78)	48
5	(*S*)-**6a**	**18e**	(2*S*)-**3a** (75)	42
6	(*R*)-**6a**	**18a**	(2*R*)-**3a** (76)	66
7	(*R*)-**6a**	**18c**	(2*R*)-**3a** (75)	80 (>96%)^c^
8	(*R*)-**6a**	**18f**	(2*R*)-**3a** (74)	40
9	(*R*)-**6a**	**18g**	(2*R*)-**3a** (72)	76
10	(*R*)-**6a**	**18h**	(2*R*)-**3a** (76)	74
11	(*R*)-**6a**	**18i**	(2*R*)-**3a** (72)	68
12	(*R*)-**6a**	**18j**	(2*R*)-**3a** (74)	60
13	(*R*)-**6a**	**18k**	(2*R*)-**3a** (78)	62
14	(*R*)-**6a**	**18l**	(2*R*)-**3a** (80)	54
15	(*S*)-**6a**	**19**	(2*S*)-**3a** (71)	34
16	(*S*)-**6a**	**20**	(2*S*)-**3a** (70)	31
17	(*R*)-**6a**	**21**	(2*R*)-**3a** (79)	48

^a^After purification. ^b^Determined by HPLC and ^1^H NMR. ^c^After flash chromatography on silica gel (EtOAc/hexanes 3:7) and crystallization from hexanes/toluene. Yield: 63%.

No de improvements resulted from the use of catalysts **18d,e** which were modified by methylation or allylation of the cinchoninium alcohol fragment (Table 2, entries 4 and 5). While using cinchoninium catalyst **18a** and the same reaction conditions, we noticed amide reagent (*R*)-**6a** led to a higher diastereomeric excess (de) of 66% for product (*R*)-**3a** as compared to reagent (*S*)-**6a** for product (*S*)-**3a**, one configuration being preferred from the other (Table 2, entry 6). A quite similar effect was previously observed in other Michael-additions involving chiral auxiliaries on the nucleophile and on the Michael acceptor [62]. As for (*S*)-**6a**, catalyst **18c** bearing a bulky *tert*-butyl group at the benzyl *para-*position gave the best results in term of stereoselectivity (80% de, Table 2, entry 7). However, the use of bulkier benzyl, naphthyl and anthracenyl fragments, e.g., catalysts **18i**–**l**, did not enhance the reaction diastereoselectivity (Table 2, entries 11–14). Whereas catalyst **18f** bearing an *ortho*-fluoro substituent led to a decrease of de (Table 2, entry 8), catalyst **18g** and **18h**, respectively, substituted at the benzyl *para*-position by isopropyl and CF_3_ groups, gave good results with 76 and 74% de (Table 2, entries 9 and 10). Dimeric and trimeric organocatalysts **19** and **20** based on a cinchonine core did not enhance the reaction diastereoselectivity (Table 2, entries 15 and 16). Finally, by comparison to all the studied cinchoninium catalysts, the use of cinchonidinium catalyst **21** proved to be less efficient with a 48% de (Table 2, entry 17).

**Scheme 3:**
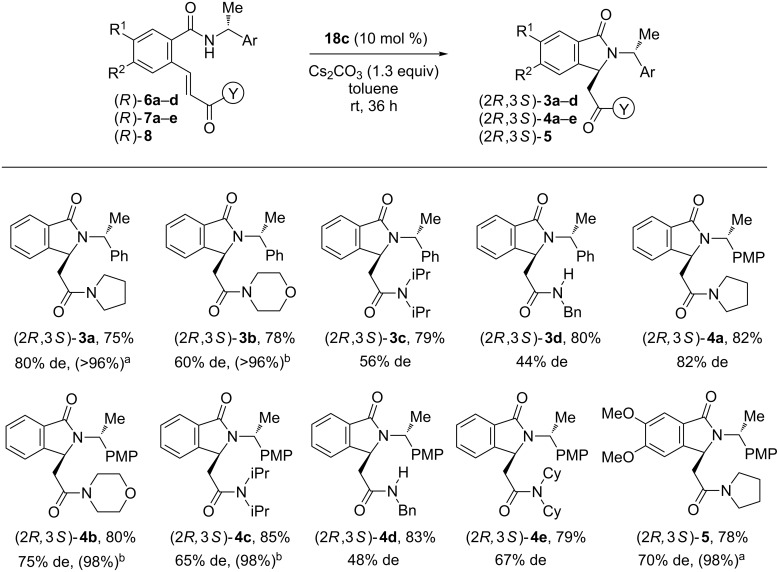
Synthesis of isoindolinones **3a**–**d, 4a**–**e, 5**; isolated yield, de by HPLC and ^1^H NMR. ^a^After flash chromatography on silica gel (EtOAc/hexanes 3:7) and crystallization from hexanes/toluene; ^b^after flash chromatography.on silica gel (EtOAc/hexanes 3:7).

With the optimized reaction conditions in hand, catalyst **18c** was employed in the asymmetric intramolecular aza-Michael reaction of benzamides (*R*)-**6a**–**d** bearing an array of acrylamide groups (Scheme 3). Substrate **6a** bearing a (*R*)-α-methylbenzyl chiral auxiliary led to isoindolinone **3a** in 80% de and a pure diastereoisomer was recovered after chromatography on silica gel (EtOAc/hexanes 3:7) and crystallization from hexanes/toluene. Reactions of substrates **6b**–**d** highlighted the diastereoselection of the reaction was highly dependent of the starting benzamide substitution, 44 to 60% de being obtained for **3b**–**d**. Finally, whereas diastereoisomers issued from **6b** could be separated by flash chromatography, this was not possible for products **3c** and **d**. Cyclisation of chiral benzamides (*R*)-**7a**–**e** and (*R*)-**8** led to isoindolinones (2*R*)-**4a**–**e**, (2*R*)-**5** in good yields and average to good diastereoselectivities (Scheme 3). In some cases, purification by flash chromatography afforded products **4b**, **4c** and **5** in higher diastereomeric purity.

**Scheme 4:**
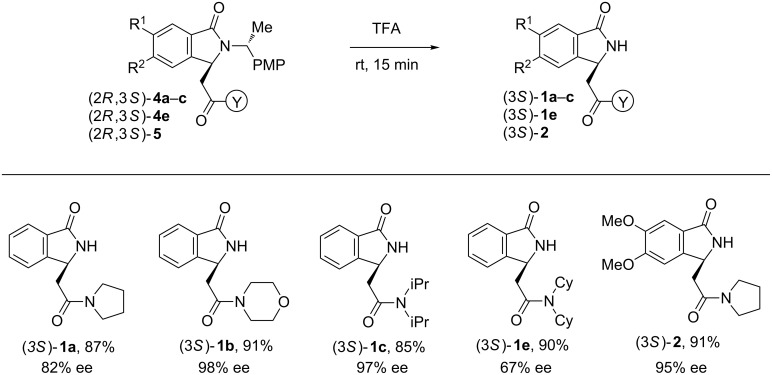
Removal of the chiral auxiliary. Synthesis of isoindolinones **1a**–**c, 1e, 2**; isolated yield, ee by HPLC.

In order to access to the targeted NH-free isoindolinones, the cleavage of the (*R*)-α-methylbenzyl chiral auxiliary was performed in acidic conditions but the reactions proved to be ineffective. However, a change in our models for the more electron rich (*R*)-α-methyl-*para*-methoxybenzyl group resulted in a straightforward and selective cleavage in mild acidic conditions without racemization (Scheme 4). Indeed, further cleavage of the α-methyl-*para*-methoxyphenyl chiral auxiliary in protected isoindolinones **4a–c**, **4e** and **5** resulted in the corresponding NH-free lactams **1a**–**c**, **1e** and **2** without any racemization (Scheme 4).

**Figure 5:**
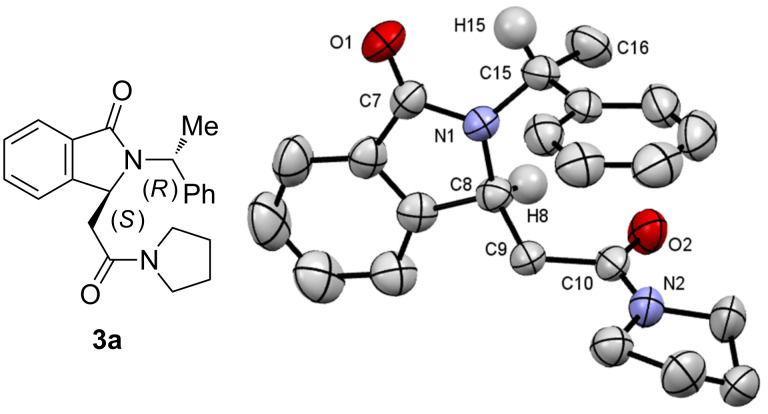
ORTEP plot of isoindolinone (2*R*,3*S*)-**3a** (CCDC 1590565) [68].

A subsequent X-ray analysis of a single crystal allowed us to assert the (2*R*,3*S*) configuration of **3a** (Figure 5). This result allowed for the determination of the absolute configurations of all isolated isoindolinones.

### Asymmetric synthesis of a new pazinaclone analogue

**Scheme 5:**
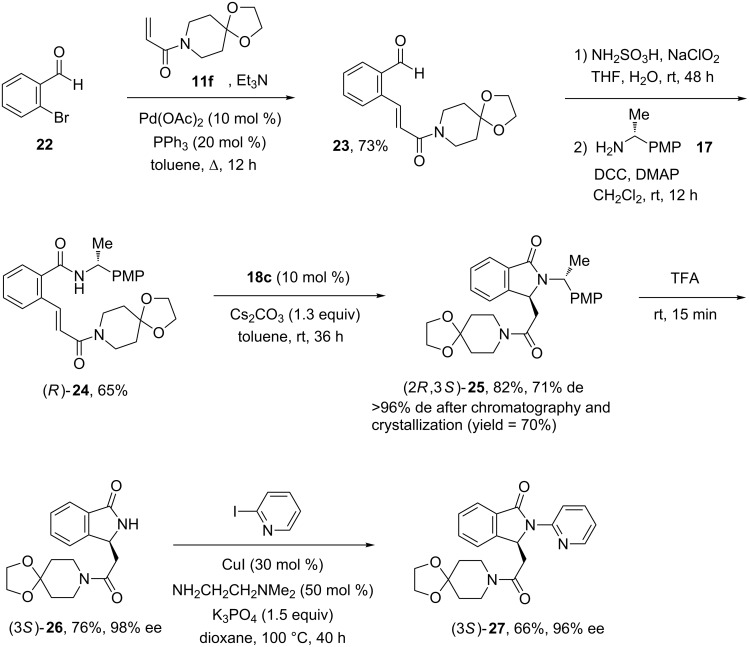
Synthesis of pazinaclone analogue (3*S*)-**27**.

With this handful methodology in hands, we then turned our attention to the asymmetric synthesis of a new pazinaclone analogue, which could be of particular interest in the field of benzodiazepine-receptor agonists [8–17]. Indeed, pazinaclone produces its sedative and anxiolytic effects by acting as a partial agonist at GABA_A_ (γ-aminobutyric acid type A) benzodiazepine receptors [17]. In order to circumvent any hydrolysis of the ketal group during the preparation of the starting benzamide (see Supporting Information File 1), the synthesis of intermediate **24** was performed according another pathway depicted in Scheme 5. Aldehyde **23** was first readily prepared via a Heck cross coupling reaction between 2-bromobenzaldehyde (**22**) and acrylamide **11f**. Next, a Pinnick oxidation of the aldehyde **23** followed with a coupling reaction with chiral benzylamine **17** delivered the targeted benzamide **24** in good yield (65%).

The intramolecular aza-Michael reaction of acrylamide (*R*)-**24** was then performed using the best phase-transfer catalyst **18c** and the optimized experimental conditions to give isoindolinone (2*R*,3*S*)-**25** as a mixture of diastereoisomers (82% yield, 71% de) which were separated by chromatography and purified by crystallization (70% yield, >96% de). Lactam (2*R*,3*S*)-**25** bearing a α-methyl-*para*-methoxyphenyl chiral auxiliary was then deprotected with trifluoroacetic acid at room temperature to deliver the NH-free isoindolinone (3*S*)-**26** (76% yield, 98% ee) which is a key building block in the synthesis of benzodiazepine-receptor agonists [8–16]. Indeed, the copper-catalyzed N-arylation of (3*S*)-**26** was performed in dioxane with *N*,*N*-dimethylethylenediamine as ligand [27] to deliver the targeted pazinaclone analogue (3*S*)-**27** in a fair yield (66%) without significant loss in enantiomeric purity. Moreover, it was worth to note compound (3*S*)-**27** was not racemizing when heated in DMF at 150 °C for 48 h.

## Conclusion

Herein, a new synthetic route towards optically active 3-substituted isoindolinones was developed. These organic compounds are useful for the development of agonists of GABA_A_ (γ-aminobutyric acid type A) benzodiazepine-receptors. Various functionalized isoindolinones were prepared in good yields and diastereomeric excesses by intramolecular aza-Michael reactions using a double stereo-induction approach. The combined use of selected cinchoninium salts as phase-transfer catalysts and of nucleophiles bearing a chiral auxiliary enabled an effective match effect between the diastereomeric ion pair formed by the nucleophile, the Michael acceptor and the cinchoninium salt. Further investigations on this synthetic methodology will be reported in due course.

## Supporting Information

File 1 File Name S1.pdf.
Experimental procedures, characterization data, copies of the ^1^H, ^13^C NMR spectra, HPLC chromatograms, ORTEP drawing of **3a** and the summary of **3a** crystallographic information.

File 2 File Name S1.cif.
Crystallographic information file of compound **3a**.
