# Supplementary material for: Diastereoselective auxiliary- and catalyst-controlled intramolecular aza-Michael reaction for the elaboration of enantioenriched 3-substituted isoindolinones. Application to the synthesis of a new pazinaclone analogue
Source: Beilstein J Org Chem. 2018 Mar 9;14:593–602. doi: 10.3762/bjoc.14.46 (PMC5852534; doi:10.3762/bjoc.14.46)
Supplement: File 1 — File Name S1.pdf. Experimental procedures, characterization data, copies of the 1H, 13C NMR spectra, HPLC chromatograms, ORTEP drawing of 3a and the summary of 3a crystallographic information. [file Beilstein_J_Org_Chem-14-593-s001.pdf]

Supporting Information

for

Diastereoselective auxiliary- and catalyst-controlled intramolecular aza-Michael reaction for the elaboration of enantioenriched 3-substituted isoindolinones. Application to the synthesis of a new pazinaclone analogue

Romain Sallio, Stéphane Lebrun, Frédéric Capet, Francine Agbossou-Niedercorn, Christophe Michon* and Eric Deniau*

Address: Univ, Lille, CNRS, Centrale Lille, ENSCL, Univ. Artois, UMR 8181-UCCS-Unité de Catalyse et Chimie du Solide, F-59000 Lille, France

Email: Christophe Michon* - christophe.michon@ensc-lille.fr;

Eric Deniau* - Eric.Deniau@univ-lille1.fr

*Corresponding author

Experimental procedures, characterization data, copies of the ^{1}H, ^{13}C NMR spectra, HPLC chromatograms, ORTEP drawing of 3a and the summary of 3a crystallographic information

1. General information. S2
2. Experimental procedures and characterizations. S2
2.1. Procedure for the preparation of acid *tert*-butyl ester **10** S3
2.2. General procedure for the synthesis of benzamide derivatives **6–8** S4
2.3. General procedure for the intramolecular aza-Michael reaction: synthesis of isoindolinone **3–5** S9
2.4. General procedure for the synthesis of NH isoindolinone **1** and **2** S14
2.5. General procedure for the synthesis of pazinaclone analogue **27** S17
2.5.1 Synthesis of benzamide derivative **24** (first strategy) S17
2.5.2 Synthesis of benzamide derivative **24** (second strategy) S19
2.5.3 Synthesis of pazinaclone analogue **27** S21
3. References. S23
4. ^{1}H and ^{13}C spectra. S24
5. HPLC for compounds S58
6. X-ray analysis of compound **3a**. S69

1.General information

Melting points were determined on a Reichert-Thermopan apparatus and are uncorrected. NMR spectra were recorded on Bruker AV 300 spectrometer and were referenced against internal tetramethylsilane; Coupling constants (*J*) are given in Hz and rounded to the nearest 0.1 Hz. IR absorption spectra were run on a Perkin-Elmer 881. HPLC analyses were performed on a Hitachi-VWR LaChromElite L-2000. Elemental analyses were obtained using a Carlo-Erba CHNS-11110 equipment. Flash chromatography was performed on Sorbent Technologies 32–63 μm 60 Å silica gel. Reactions were monitored by thin-layer chromatography with Sorbent Technologies 0.20 mm silica gel 60 Å plates. Dry glassware was obtained by oven-drying and assembly under inert gas. Dry nitrogen was used as the inert atmosphere. The glassware was equipped with rubber septa and reagent transfers were performed by syringe techniques. Tetrahydrofuran (THF) was distilled from sodium benzophenone ketyl prior to use. Methanol (MeOH), ethanol (EtOH) and isopropanol (*i*-PrOH) were distilled over magnesium turnings, CH_2Cl_2 over CaH_2 and toluene over sodium.

2. Experimental procedures and characterizations

Retrosynthetic analysis of chiral 3-substituted isoindolinones.

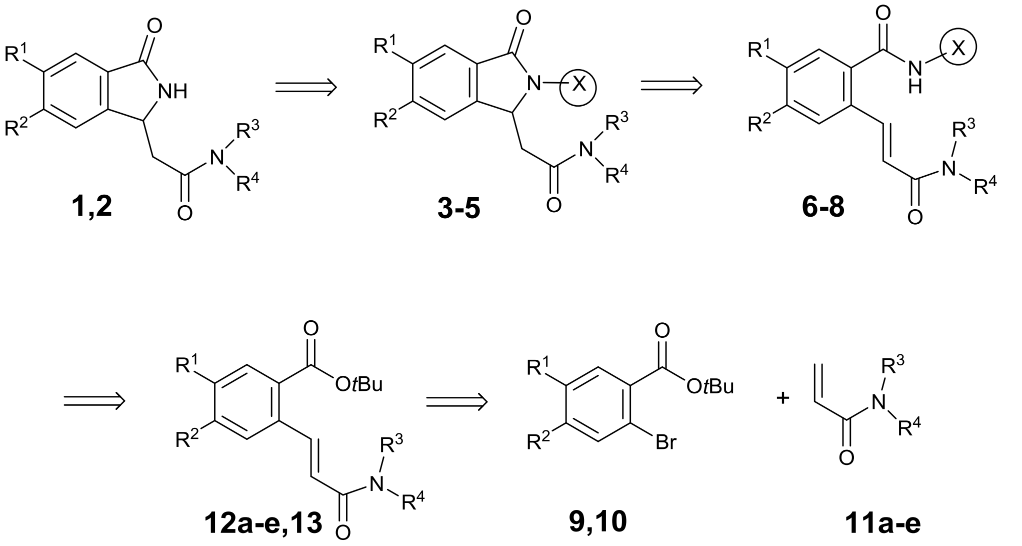

Benzamides **6–8** were prepared in few steps. See the following text for details. 2-Bromobenzoic acid *tert*-butyl ester **9** and **10** were prepared according to reported procedures.[1] Acrylamides **11a–e** were synthesized following literature methods.[2]

2.1. Procedure for the preparation of acid tert-butyl ester **13**

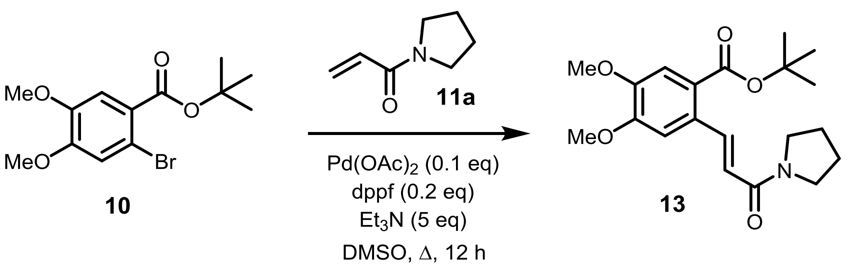

To a solution of 2-Bromo-4,5-dimethoxybenzoic acid *tert*-butyl ester **10** (1.24 g, 3.9 mmol) in DMSO (20 mL) maintained under nitrogen atmosphere, were added $Pd(OAc)_2$ (88 mg, 10 mol %), dppf (431 mg, 20 mol %), Et_3N (2.7 mL, 19.5 mmol) and the corresponding acrylamide **11a** (975 mg, 7.8 mmol). The mixture was stirred for 12 h at reflux, and then it was diluted with water (5 mL) and extracted with CH_2Cl_2 (3 × 50 mL). The combined organic layers were dried over $MgSO_4$ and concentrated under vacuum to give an oil which was purified by flash column chromatography on silica gel using EtOAc/hexanes (50:50) as eluent. Evaporation of solvents under vacuum afforded compound **13**.

4,5-Dimethoxy-2-((E)-3-oxo-3-(pyrrolidin-1-yl)propenyl)benzoic acid tert-butyl ester (**13**)

Yield 69% (971 mg). Mp 142-143°C. ^{1}H NMR (300 MHz, $CDCl_3$): δ = 1.62 (s, 9 H, 3 x CH_3), 1.88-2.02 (m, 4 H, 2 x CH_2), 3.57-3.66 (m, 4 H, 2 x CH_2), 3.93 (s, 3 H, OCH_3), 3.95 (s, 3 H, OCH_3), 6.47 (d, J = 15.4 Hz, 1 H, =CH), 6.97 (s, 1 H, H_{arom}), 7.42 (s, 1 H, H_{arom}), 8.37 (d, J = 15.4 Hz, 1 H, =CH). ^{13}C NMR (75 MHz, $CDCl_3$): **C** 166.2 (CO), 164.6 (CO), 151.4, 149.2, 130.7, 124.9, 82.2, **CH** 141.5, 119.9, 113.1, 109.9, **CH_2** 46.6, 46.0, 26.2, 24.3, **CH_3** 56.0 (2 x OCH_3), 28.2 (3 x CH_3).

2.2. General procedure for the synthesis of benzamide derivatives **6–8**

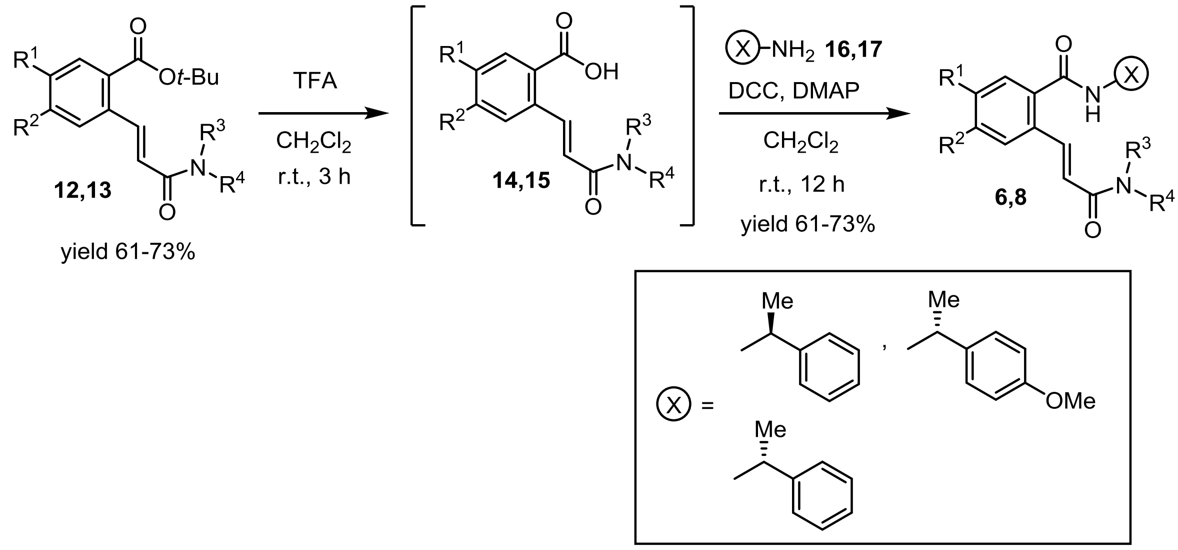

To a solution of ester **12,13** (2 mmol) in dry CH_2Cl_2 (10 mL) was added trifluoroacetic acid (1.54 mL, 20 mmol). The mixture was stirred at room temperature for 3 h. The mixture was concentrated to dryness, and the residue was washed successively with 3 × 30 mL of EtOAc to afford the corresponding acid which was used for the next step without further purification. To a stirred solution of benzoic acid derivatives (2 mmol) in anhydrous CH_2Cl_2 (20 mL) under argon, were added *N*,*N*'-dicyclohexylcarbodiimide (412 mg, 2 mmol), *N*,*N*-(dimethylamino)pyridine (25 mg, 0.2 mmol) and the appropriate amine, i.e., (*S*)-**16** or (*R*)-**16** or (*R*)-**17** (2 mmol). After stirring for 12 h, water (30 mL) was added and the aqueous layer was extracted with CH_2Cl_2 (2 × 20 mL). The organic layer was dried $MgSO_4$, filtered and concentrated. Purification by flash chromatography over silica gel using EtOAc/hexanes (80/20) afforded benzamide derivatives followed par recrystallization *(S)*-**6a,** (*R*)-**6a-d,** (*R*)-**7a-e** and (*R*)-**8.**

*2-((E)-3-Oxo-3-(pyrrolidin-1-yl)propenyl)-N-((S)-1-phenylethyl)benzamide ((S)-***6a***)*

Yield 75% (522 mg). Mp 140-141°C. R*f* (100% EtOAc) 0.55. $[\alpha]_D^{20}$ -18.5 (*c* 0.86, $CHCl_3$). IR (cm^{-1}): 2942, 1653, 1639, 1597, 1435, 1311, 758, 696. ^1H NMR (300 MHz, $CDCl_3$): δ = 1.61 (d, *J* = 6.9 Hz, 3 H, CH_3), 1.83-1.94 (m, 4 H, 2 x CH_2), 3.31-3.54 (m, 4 H, 2 x CH_2), 5.24-5.34 (m, 1 H, NCH), 6.54 (d, *J* = 15.5 Hz, 1 H, =CH), 6.56 (brs, 1 H, NH), 7.25-7.50 (m, 9 H, H_{arom}), 7.83 (d, *J* = 15.5 Hz, 1 H, CH=). ^{13}C NMR (75 MHz, $CDCl_3$): **C** 168.2 (CO), 164.2 (CO), 143.1, 136.9, 133.5, **CH** 138.7, 129.9, 128.9, 128.7 (2 x CH), 127.9, 127.8,

127.3, 126.4 (2 x CH), 122.5, 49.6, **CH_2** 46.6, 46.0, 26.1, 24.3, **CH_3** 22.1. Anal. Calcd for $C_{22}H_{24}N_2O_2$: C, 75.83; H, 6.94; N, 8.04 %. Found: C, 75.92; H, 7.01; N, 7.92%.

2-((E)-3-Oxo-3-(pyrrolidin-1-yl)propenyl)-N-((R)-1-phenylethyl)benzamide ((*R*)-**6a**)

Yield 70% (487 mg). Mp 140-141°C. R*f* (100% EtOAc) 0.55. $[\alpha]_D^{20}$ +18.5 (*c* 0.54, $CHCl_3$). IR (cm^{-1}): 2942, 1653, 1639, 1597, 1435, 1311, 758, 696. ^1H NMR (300 MHz, $CDCl_3$): δ = 1.61 (d, *J* = 6.9 Hz, 3 H, CH_3), 1.83-1.94 (m, 4 H, 2 x CH_2), 3.31-3.54 (m, 4 H, 2 x CH_2), 5.30 (m, 1 H, NCH), 6.54 (d, *J* = 15.5 Hz, 1 H, =CH), 6.56 (brs, 1 H, NH), 7.25-7.50 (m, 9 H, H_{arom}), 7.83 (d, *J* = 15.5 Hz, 1 H, CH=). ^{13}C NMR (75 MHz, $CDCl_3$): **C** 168.2 (CO), 164.2 (CO), 143.1, 136.9, 133.5, **CH** 138.7, 129.9, 128.9, 128.7 (2 x CH), 127.9, 127.8, 127.3, 126.4 (2 x CH), 122.5, 49.6, **CH_2** 46.6, 46.0, 26.1, 24.3, **CH_3** 22.1. HRMS (ESI+) m/z calcd for $C_{22}H_{25}O_2N_2$ $[MH]^+$ 349.19105, found 349.19135.

2-((E)-3-(Morpholin-4-yl)-3-oxopropenyl)-N-((R)-1-phenylethyl)benzamide ((*R*)-**6b**)

Yield 64% (466 mg). Mp 178-179°C. R*f* (100% EtOAc) 0.69. $[\alpha]_D^{20}$ + 35.5 (*c* 0.72, $CHCl_3$). IR (cm^{-1}): 2972, 1635, 1544, 1114, 1062, 760. ^1H NMR (300 MHz, $CDCl_3$): δ = 1.61 (d, *J* = 6.9 Hz, 3 H, CH_3), 3.43-3.71 (m, 8 H, 4 x CH_2), 5.31 (m, 1 H, NCH), 6.12 (brd, *J* = 7.9 Hz, 1 H, NH), 6.68. (d, *J* = 15.6 Hz, 1 H, =CH), 7.27-7.56 (m, 9 H, H_{arom}), 7.79 (d, *J* = 15.6 Hz, 1 H, CH=). ^{13}C NMR (75 MHz, $CDCl_3$): **C** 168.0 (CO), 165.4 (CO), 143.4, 136.7, 133.2, **CH** 139.9, 129.7, 128.9, 128.6 (2 x CH), 127.7, 127.3, 127.2, 126.2 (2 x CH), 119.6, 49.4, **CH_2** 66.6 (4 x CH_2), **CH_3** 22.1.HRMS (ESI+) m/z calcd for $C_{22}H_{25}O_3N_2$ $[MH]^+$ 365.18597, found 365.18649.

2-((E)-2-(Diisopropylcarbamoyl)vinyl)-N-((R)-1-phenylethyl)benzamide ((*R*)-**6c**)

Yield 61% (461 mg). Mp 147-148°C. R*f* (100% EtOAc) 0.68. $[\alpha]_D^{20}$ + 16.7 (*c* 0.21, $CHCl_3$). IR (cm^{-1}): 2929, 1639, 1625, 1589, 1541, 1442, 1338, 758, 700. ^1H NMR (300 MHz, $CDCl_3$): δ = 1.20-1.42 (m, 12 H, 4 x CH_3), 1.67 (d, *J* = 6.8 Hz, 3 H, CH_3), 3.65 (brs, 1 H, CH), 3.99 (br

s, 1 H, CH), 5.24-5.32 (m, 1 H, NCH), 6.36 (d, *J* = 15.5 Hz, 1 H, =CH), 7.20-7.48 (m, 10 H, 9 H_{arom} + NH), 7.61 (d, *J* = 15.5 Hz, 1 H, CH=). ^{13}C NMR (75 MHz, $CDCl_3$): **C** 168.2 (CO), 166.1 (CO), 144.2, 137.5, 133.6, **CH** 137.3, 129.3, 128.6 (2 x CH), 128.3, 128.0, 127.1, 126.55, 126.5 (2 x CH), 122.4, 49.6, 48.5, 45.8, **CH_3** 22.4, 21.3 (2 x CH_3), 20.8, 20.6. HRMS (ESI+) m/z calcd for $C_{24}H_{31}O_2N_2$ $[MH]^+$ 379.23800, found 379.23856.

2-((E)-2-(Benzylcarbamoyl)vinyl)-N-((R)-1-phenylethyl)benzamide ((*R*)-**6d**)

Yield 66% (507 mg). Mp 155-156°C. R*f* (100% EtOAc) 0.43. $[\alpha]_D^{20}$ + 27.4 (*c* 0.56, $CHCl_3$). IR (cm^{-1}): 3271, 3061, 1635, 1620, 1537, 1334, 1224, 972, 752. ^{1}H NMR (300 MHz, $CDCl_3$): δ = 1.54 (d, *J* = 7.0 Hz, 3 H, CH_3), 4.33 (dd, *J* = 5.7, 14.8 Hz, 1 H, NCH_2Ph), 4.44 (dd, *J* = 6.0, 14.8 Hz, 1 H, NCH_2Ph), 5.10-5.21 (m, 1 H, NCH), 5.98 (d, *J* = 15.7 Hz, 1 H, =CH), 6.77-6.84 (m, 1 H, H_{arom}), 6.95-7.37 (m, 15 H, 13 H_{arom} + 2 x NH), 7.54 (d, *J* = 15.7 Hz, 1 H, CH=). ^{13}C NMR (75 MHz, $CDCl_3$): **C** 168.3 (CO), 165.8 (CO), 143.8, 138.6, 136.7 132.9, **CH** 137.7, 129.7, 128.6 (2 x CH), 128.5 (2 x CH), 128.0 (2 x CH), 127.3, 127.25 (2 x CH), 127.2, 127.0 (2 x CH), 126.4, 123.2, 49.6, **CH_2** 43.6, **CH_3** 21.9. HRMS (ESI+) m/z calcd for $C_{25}H_{25}O_2N_2$ $[MH]^+$ 385.19105, found 385.19156.

N-((R)-1-(4-Methoxyphenyl)ethyl)-2-((E)-3-oxo-3(-pyrrolidin-1-yl)propenyl)benzamide ((*R*)-**7a**)

Yield 73% (552 mg). Mp 162-163°C. R*f* (100% EtOAc) 0.62. $[\alpha]_D^{20}$ + 17.1 (*c* 0.47 , $CHCl_3$). IR (cm^{-1}): 3253, 2976, 1639, 1597, 1512, 1431, 1236, 1184, 1008, 835, 763. ^{1}H NMR (300 MHz, $CDCl_3$): δ = 1.59 (d, *J* = 6.9 Hz, 3 H, CH_3), 1.84-1.97 (m, 4 H, 2 x CH_2), 3.37-3.56 (m, 4 H, 2 x CH_2), 3.78 (s, 3 H, OCH_3), 5.21-5.31 (m, 1 H, NCH), 6.20 (brd, 1 H, NH), 6.57 (d, *J* = 15.5 Hz, 1 H, =CH), 6.87 (d, *J* = 8.7 Hz, 2 H, H_{arom}), 7.31 (d, *J* = 8.7 Hz, 2 H, H_{arom}), 7.33-7.49 (m, 4 H, H_{arom}), 7.85 (d, *J* = 15.5 Hz, 1 H, CH=). ^{13}C NMR (75 MHz, $CDCl_3$): **C** 168.0 (CO), 164.3 (CO), 158.8, 136.9, 135.1, 133.5, **CH** 138.7, 129.9, 128.9, 127.9, 127.8, 127.6 (2 x CH), 122.6, 114.0 (2 x CH), 49.0, 48.5, 45.8, **CH_2** 46.6, 46.0, 26.1, 24.3 **CH_3** 55.3, 21.9. HRMS (ESI+) m/z calcd for $C_{23}H_{27}O_3N_2$ $[MH]^+$ 379.20162, found 379.20248.

N-((R)-1-(4-Methoxyphenyl)ethyl)-2-((E)-3-morpholin-4-yl-3-oxo-propenyl)-benzamide ((*R*)-**7b**)

Yield 72% (567 mg). Mp 188-189°C. R*f* (100% EtOAc) 0.69. $[\alpha]_D^{20}$ + 26.7 (*c* 0.61, $CHCl_3$). IR (cm^{-1}): 3278, 3068, 1649, 1627, 1606, 1514, 1425, 1255, 1184, 1112, 1037, 974, 833, 761. ^1H NMR (300 MHz, $CDCl_3$): δ = 1.59 (d, *J* = 6.9 Hz, 3 H, CH_3), 3.41-3.66 (m, 8 H, 4 x CH_2), 3.80 (s, 3 H, OCH_3), 5.21-5.30 (m, 1 H, NCH), 6.07 (brd, *J* = 7.8 Hz, 1 H, NH), 6.65 (d, *J* = 15.6 Hz, 1 H, =CH), 6.84 (d, *J* = 8.7 Hz, 2 H, H_{arom}), 7.30 (d, *J* = 8.7 Hz, 2 H, H_{arom}), 7.32-7.55 (m, 4 H, H_{arom}), 7.78 (d, *J* = 15.6 Hz, 1 H, CH=). ^{13}C NMR (75 MHz, $CDCl_3$): **C** 168.0 (CO), 165.4 (CO), 159.0, 136.7, 135.0, 133.6, **CH** 139.8, 130.1, 129.1, 127.7 (2 x CH), 127.5, 120.6, 114.1 (2 x CH), 49.0, **CH_2** 66.8 (4 x CH_2), **CH_3** 55.3, 21.8. HRMS (ESI+) m/z calcd for $C_{23}H_{27}O_4N_2$ $[MH]^+$ 395.19653, found 395.19739.

2-((E)-2-(Diisopropylcarbamoyl)vinyl)-N-((R)-1-4-(methoxyphenyl)ethyl)benzamide ((*R*)-**7c**)

Yield 68% (555 mg). Mp 98-99°C. R*f* (100% EtOAc) 0.71. $[\alpha]_D^{20}$ + 11.6 (*c* 0.57, $CHCl_3$). IR (cm^{-1}): 3228, 3061, 1643, 1593, 1512, 1442, 1371, 1338, 1246, 1178, 1043, 970, 831, 759. ^1H NMR (300 MHz, $CDCl_3$): δ = 1.20-1.46 (m, 12 H, 4 x CH_3), 1.62 (d, *J* = 6.9 Hz, 3 H, CH_3), 3.78 (s, 3 H, OCH_3), 3.86-4.01 (brs, 2 H, 2 x NCH), 5.21-5.32 (m, 1 H, NCH), 6.58 (d, *J* = 15.5 Hz, 1 H, =CH), 6.71 (brd, 1 H, NH), 6.88 (d, *J* = 8.7 Hz, 2 H, H_{arom}), 7.22-7.47 (m, 6 H, H_{arom}), 7.65 (d, *J* = 15.5 Hz, 1 H, CH=). ^{13}C NMR (75 MHz, $CDCl_3$): **C** 168.0 (CO), 165.9 (CO), 158.9, 137.0, 135.5, 134.0, **CH** 137.4, 129.7, 128.5, 127.9, 127.6 (2 x CH), 127.0, 123.8, 114.1 (2 x CH), 49.0, **CH_3** 55.3, 21.9, 21.4, 21.2, 20.7. HRMS (ESI+) m/z calcd for $C_{25}H_{33}O_3N_2$ $[MH]^+$ 409.24857, found 409.24927.

2-((E)-2-(Benzylcarbamoyl)vinyl)-N-((R)-1-(4-methoxyphenyl)ethyl)benzamide ((*R*)-**7d**)

Yield 61% (505 mg). Mp 161-162°C. R*f* (100% EtOAc) 0.45. $[\alpha]_D^{20}$ + 80.1 (*c* 0.54, $CHCl_3$). IR (cm^{-1}): 3277, 3066, 1631, 1622, 1541, 1512, 1246, 1029, 829, 754. ^1H NMR (300 MHz, $CDCl_3$): δ = 1.53 (d, *J* = 6.9 Hz, 3 H, CH_3), 3.74 (s, 3 H, OCH_3), 4.37 (dd, *J* = 5.7, 14.8 Hz, 1 H, NCH_2Ph), 4.45 (dd, *J* = 6.0, 14.8 Hz, 1 H, NCH_2Ph), 5.09-5.18 (m, 1 H, NCH), 6.03 (d, *J* = 15.7 Hz, 1 H, =CH), 6.81-7.26 (m, 13 H, H_{arom}), 7.60 (d, *J* = 15.7 Hz, 1 H, CH=). ^{13}C NMR

(75 MHz, $CDCl_3$): **C** 168.1 (CO), 165.7 (CO), 158.8, 138.7, 136.8, 135.5, 132.9, **CH** 137.7, 129.8, 128.7, 128.6 (2 x CH), 127.9 (2 x CH), 127.6 (2 x CH), 127.5, 127.3, 127.2, 123.6, 114.0 (2 x CH), 49.0, **CH_2** 43.6, **CH_3** 55.3, 21.8. HRMS (ESI+) m/z calcd for $C_{26}H_{27}O_3N_2$ $[MH]^+$ 415.20162, found 415.20255.

2-((E)-3-(Dicyclohexylamino)-3-oxo-propen-1-yl)-N-((R)-1-(4-methoxyphenyl)ethyl)-benzamide ((*R*)-**7e**)

Yield 66% (644 mg). Mp 87-88°C. R*f* (100% EtOAc) 0.64. $[\alpha]_D^{20}$ + 11.0 (*c* 0.59, $CHCl_3$). IR (cm^{-1}): 3257, 2931, 1635, 1512, 1452, 1246, 1178, 1031, 829, 758. ^1H NMR (300 MHz, $CDCl_3$): δ = 1.21-1.38 (m, 6 H), 1.49-1.82 (m, 15 H), 2.11-2.40 (m, 2 H), 3.12-3.55 (m, 2 H), 3.78 (s, 3 H, OCH_3), 5.22-5.33 (m, 1 H, NCH), 6.46 (d, *J* = 15.5 Hz, 1 H, =CH), 6.88 (d, *J* = 8.8 Hz, 2 H, H_{arom}), 7.20-7.41 (m, 6 H, H_{arom}), 7.59 (d, *J* = 15.5 Hz, 1 H, CH=). ^{13}C NMR (75 MHz, $CDCl_3$): **C** 167.9 (CO), 166.2 (CO), 158.7, 137.3, 136.0, 129.4, **CH** 137.35, 129.5, 128.3, 127.9, 127.6 (2 x CH), 126.8, 123.4, 114.0 (2 x CH), 57.8, 56.0, 49.0, **CH_2** 31.8, 31.7, 30.2, 30.1, 26.6, 26.2, 26.0, 25.4, **CH_3** 55.2, 22.1. HRMS (ESI+) m/z calcd for $C_{31}H_{41}O_3N_2$ $[MH]^+$ 489.31117, found 489.31259.

(E)-3,4-Dimethoxy-N-((R)-1-(4-methoxyphenyl)ethyl)-2-((E)-3-oxo-(3-pyrrolidin-1-yl)propen-1-yl)benzamide ((R)-**8**)

Yield 72% (631 mg). Mp 161-162°C. R*f* (100% EtOAc) 0.58. $[\alpha]_D^{20}$ -11.8 (*c* 1.15, $CHCl_3$). IR (cm^{-1}): 3441, 2972, 1631, 1604, 1512, 1444, 1361, 1271, 1247, 1211, 1178, 1076, 1035, 837. ^1H NMR (300 MHz, $CDCl_3$): δ = 1.60 (d, *J* = 7.0 Hz, 3 H, CH_3), 1.79-1.92 (m, 4 H), 3.38-3.52 (m, 4 H), 3.76 (s, 3 H, OCH_3), 3.79 (s, 3 H, OCH_3), 3.85 (s, 3 H, OCH_3), 5.17-5.25 (m, 1 H, NCH), 6.37 (d, *J* = 15.5 Hz, 1 H, =CH), 6.81-6.88 (m, 4 H, H_{arom}), 7.02 (br d, 1 H, NH), 7.35 (d, *J* = 8.7 Hz, 2 H, H_{arom}), 7.76 (d, *J* = 15.5 Hz, 1 H, CH=). ^{13}C NMR (75 MHz, $CDCl_3$): **C** 167.5 (CO), 164.3 (CO), 158.8, 150.1, 149.8, 135.1, 130.0, 126.4, **CH** 138.7, 127.6 (2 x CH), 121.1, 114.0 (2 x CH), 111.2, 109.9, 49.3, **CH_2** 46.6, 46.0, 26.1, 24.3, **CH_3** 56.1 (2 x OCH_3), 55.3, 22.0. HRMS (ESI+) m/z calcd for $C_{25}H_{31}O_5N_2$ $[MH]^+$ 439.22275, found 439.22327.

2.3. General procedure for the intramolecular aza-Michael reaction: synthesis of isoindolinone **3–5**

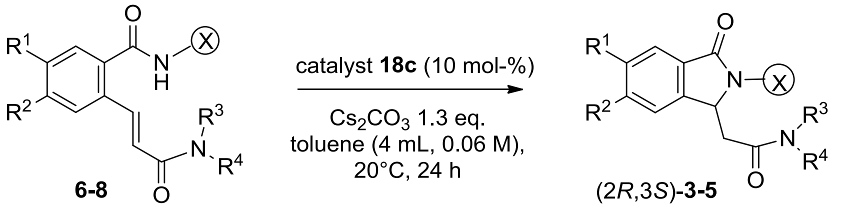

A mixture of benzamide (*S*)-**6a,** *(R)*-**6a–d,** *(R)*-**7a–e** and (*R*)-**8** (0.08 mmol), base (1.3 equiv) and catalyst **18c** (10 mol %) was stirred for 6 to 36 h (Table 2) in toluene (2 mL) at room temperature. The resulting reaction mixtured was monitored by TLC till completion. The crude product was purified by flash chromatography on silica gel by eluting with EtOAc/Hexanes to afford after evaporation of solvents under vacuum product (*S*)-**3a,** (*R*)-**3a-d,** (*R*)-**4a–e** and (*R*)-**5** as a mixture of diastereoisomers or a single diastereoisomer. The diastereomeric excess for compounds (*S*)-**3a,** (*R*)-**3a–d,** (*R*)-**4a–e** and (*R*)-**5** were determined by ^{1}H NMR and/or HPLC.

(2S,3S)-3-(2-Oxo-2-(pyrrolidin-1-yl)ethyl)-2-(1-phenylethyl)-2,3-dihydro-isoindol-1-one ((*S*)-**3a**)

Yield 77% (21.4 mg); 62% de. R*f* (40% EtOAc/Hexane) 0.46. $[\alpha]_D^{20}$ -60.8 (*c* 1.05, $CHCl_3$). IR (cm^{-1}): 1678, 1635, 1442, 1402, 1159, 740, 692. ^{1}H NMR (300 MHz, $CDCl_3$) of the major diastereoisomer: δ = 1.71-1.77 (m, 4 H, 2 CH_2), 1.82 (d, *J* = 7.3 Hz, 3 H, CH_3), 2.05 (dd, *J* = 16.5 , 7.3 Hz, 1 H, CH_2), 2.29 (dd, *J* = 16.5, 5.1 Hz, 1 H, CH_2), 2.48-2.57 (m, 1 H, CH_2), 2.78-2.87 (m, 1 H, CH_2), 3.35-3.46 (m, 4 H, 2 CH_2), 5.40 (dd, *J* = 7.3, 5.1 Hz, 1 H, CH), 5.85 (q, *J* = 7.2 Hz, 1 H, CH), 7.23-7.31 (m, 5 H, H_{arom}), 7.42-7.49 (m, 3 H, H_{arom}) 7.86-7.91 (m, 1 H, H_{arom}). ^{13}C NMR (75 MHz, $CDCl_3$) of the major diastereoisomer: **C** 168.7 (CO), 167.8 (CO), 146.6, 142.0, 131.7, **CH** 131.8, 128.3 (3 x CH), 127.4 (2 x CH), 127.1, 123.7, 122.9, 55.1, 48.6, **CH_2** 46.0, 45.6, 39.8, 25.9, 24.3, **CH_3** 16.5.

(2R,3S)-3-(2-Oxo-2-(pyrrolidin-1-yl)ethyl)-2-(1-phenylethyl)-2,3-dihydro-isoindol-1-one ((*R*)-**3a**)

Yield 75% (20.9 mg); 80% de by NMR. Purification by flash chromatography on silica gel by eluting with EtOAc/Hexanes (3/7) and crystallization from hexanes/toluene furnish the major diastereoisomer (yield 63%, 97% de). Mp 154-155°C. R*f* (40% EtOAc/Hexane) 0.46. $[\alpha]_D^{20}$

+62.0 (*c* 0.5, $CHCl_3$). IR (cm^{-1}): 1689, 1627, 1446, 1388, 1149, 758, 698. ^1H NMR (300 MHz, $CDCl_3$) of the major diastereoisomer: δ = 1.71-1.77 (m, 4 H, 2 CH_2), 1.82 (d, *J* = 7.3 Hz, 3 H, CH_3), 2.05 (dd, *J* = 7.3, 16.5 Hz, 1 H, CH_2), 2.29 (dd, *J* = 16.5, 5.1 Hz, 1 H, CH_2), 2.48-2.57 (m, 1 H, CH_2), 2.78-2.87 (m, 1 H, CH_2), 3.35-3.46 (m, 4 H, 2 CH_2), 5.40 (dd, *J* = 7.3, 5.1 Hz, 1 H, CH), 5.85 (q, *J* = 7.2 Hz, 1 H, CH), 7.23-7.31 (m, 5 H, H_{arom}), 7.42-7.49 (m, 3 H, H_{arom}) 7.86-7.91 (m, 1 H, H_{arom}). ^{13}C NMR (75 MHz, $CDCl_3$) of the major diastereoisomer : **C** 168.7 (CO), 167.8 (CO), 146.6, 142.0, 131.7, **CH** 131.8, 128.3 (3 x CH), 127.4 (2 x CH), 127.1, 123.7, 122.9, 55.1, 48.6, **CH_2** 46.0, 45.6, 39.8, 25.9, 24.3, **CH_3** 16.5. HRMS (ESI+) m/z calcd for $C_{22}H_{25}O_2N_2$ $[MH]^+$ 349.19105, found 349.19159.

(2R,3S)-3-(2-(Morpholin-4-yl)-2-oxoethyl)-2-(1-phenylethyl)-2,3-dihydro-isoindol-1-one ((*R*)-**3b**)

Yield 78% (22.7 mg); 60% d.e. by HPLC using C18 Grav-1 CSP (Macherey), at 25°C, with (80/20) H_2O/CH_3CN, 1 mL/min, λ 196 nm. Purification by flash chromatography on silica gel by eluting with EtOAc/Hexanes (3/7) furnish the major diastereoisomer (yield 60%, 97% de). R*f* (40% EtOAc/Hexane) 0.51. IR (cm^{-1}): 1683, 1641, 1467, 1234, 1114, 756, 696. ^1H NMR (300 MHz, $CDCl_3$) of the major diastereoisomer : δ = 1.79 (d, *J* = 7.3 Hz, 3 H, CH_3), 1.99 (dd, *J* = 16.7 , 8.2 Hz, 1 H, CH_2), 2.28 (dd, *J* = 16.7, 4.4 Hz, 1 H, CH_2), 2.73-2.78 (m, 1 H, CH_2), 2.83-2.90 (m, 1 H, CH_2), 3.37-3.41 (m, 2 H, CH_2), 3.43-3.66 (m, 4 H, 2 CH_2), 5.38 (dd, *J* = 8.2, 4.0 Hz, 1 H, CH), 5.86 (q, *J* = 7.2 Hz, 1 H, CH), 7.24-7.35 (m, 3 H, H_{arom}), 7.45-7.52 (m, 5 H, H_{arom}) 7.87-7.91 (m, 1 H, H_{arom}). ^{13}C NMR (75 MHz, $CDCl_3$) of the major diastereoisomer : **C** 168.7 (CO), 168.2 (CO), 146.5, 142.0, 131.5, **CH** 131.8, 128.4, 128.3 (2 x CH), 127.4 (2 x CH), 127.3, 123.7, 123.1, 55.1, 48.7, **CH_2** 66.7, 66.2, 45.3, 41.9, 37.5, **CH_3** 16.6. HRMS (ESI+) m/z calcd for $C_{22}H_{25}O_3N_2$ $[MH]^+$ 365.18597, found 365.18701.

(2R,3S)-N,N-Diisopropyl-2-[3-oxo-2-(1-phenylethyl)-2,3-dihydro-1H-isoindol-1-yl]acetamide ((*R*)-**3c**)

Yield 79% (23.9 mg); 56% d.e. by HPLC using C18 Grav-1 CSP (Macherey), at 25°C, with (60/40) H_2O/CH_3CN, 1 mL/min, λ 197 nm). R*f* (40% EtOAc/Hexane) 0.60. IR (cm^{-1}): 1687, 1625, 1573, 1446, 1213, 758, 696. ^1H NMR (300 MHz, $CDCl_3$) of the major diastereoisomer: δ = 0.87 (d, *J* = 6.7 Hz, 3 H, CH_3), 0.88 (d, *J* = 6.7 Hz, 3 H, CH_3), 1.37 (d, *J* = 6.7 Hz, 3 H, CH_3), 1.38 (d, *J* = 6.7 Hz, 3 H, CH_3), 1.85-1.92 (m, 1 H, CH_2), 2.37 (dd, *J* =

16.1 , 3.7 Hz, 1 H, CH_2), 3.28 (sept, *J* = 6.7 Hz, 1 H, CH), 3.51-3.60 (brs 1 H, CH), 5.38 (dd, *J* = 9.8, 3.5 Hz, 1 H, CH), 5.77 (q, *J* = 7.2 Hz, 1 H, CH), 7.23-7.34 (m, 3 H, H_{arom}), 7.41-7.53 (m, 5 H, H_{arom}) 7.85-7.89 (m, 1 H, H_{arom}). ^{13}C NMR (75 MHz, $CDCl_3$) of the major diastereoisomer : **C** 168.7 (CO), 168.5 (CO), 146.7, 142.1, 132.3, **CH** 131.7, 128.4 (3 x CH), 128.2, 127.3 (2 x CH), 127.2, 123.5, 123.4, 56.0, 49.0, 45.7, **CH_2** 34.0, **CH_3** 20.8 (2 CH_3), 20.7, 20.5, 16.9. HRMS (ESI+) m/z calcd for $C_{24}H_{31}O_2N_2$ $[MH]^+$ 379.23800, found 379.23868.

(2R,3S)-N-Benzyl-2-(3-Oxo-2-(1-phenyl-ethyl)-2,3-dihydro-1H-isoindol-1-yl]-acetamide ((*R*)-**3d**)

Yield 80% (24.6 mg); 44% D.e by NMR. R*f* (40% EtOAc/Hexane) 0.36. IR (cm^{-1}): 3321, 2927, 1662, 1647, 1512, 1446, 1357, 1247, 1211, 1174, 1076, 1029, 839, 750. ^1H NMR (300 MHz, $CDCl_3$) of the major diastereoisomer: δ = 1.76 (d, *J* = 6.7 Hz, 3 H, CH_3), 1.86 (dd, *J* = 6.7, 14.9 Hz, 1 H, CH_2CO), 2.45 (dd, *J* = 4.7, 14.9 Hz, 1 H, CH_2CO), 4.15-4.46 (m, 2 H, NCH_2Ph), 5.24 (dd, *J* = 4.7, 8.5 Hz, 1 H, CH), 5.64-5.72 (m, 1 H, NCH), 7.13-7.44 (m, 13 H, H_{arom}), 7.77-7.83 (m, 1 H, H_{arom}). ^{13}C NMR (75 MHz, $CDCl_3$) of the major diastereoisomer: **C** 169.3 (CO), 168.8 (CO), 145.6, 141.8, 137.9, **CH** 131.8 (2 x CH), 128.7 (2 x CH), 128.6 (2 x CH), 128.1 (2 x CH), 127.7, 127.5, 127.2 (2 x CH), 123.7, 122.9, 56.2, 49.7, **CH_2** 43.8, 29.8, **CH_3** 17.2. HRMS (ESI+) m/z calcd for $C_{25}H_{25}O_2N_2$ $[MH]^+$ 385.19105, found 385.19202.

(2R,3S)-2-(1-(4-Methoxyphenyl)ethyl)-3-(2-oxo-2-(pyrrolidin-1-yl)ethyl))-2,3-dihydroisoindol-1-one ((*R*)-**4a**)

Yield 82% (24.8 mg); 82% de by HPLC using C18 Grav-1 CSP (Macherey), at 25 °C, with (70/30) H_2O/CH_3CN, 1 mL/min, λ 198 nm; or using Whelk01 CSP, at 25 °C, with (75/25) n-hexane/EtOH, 1 mL/min, λ 202 nm; or using IA CSP, at 25 °C, with (9/1) n-hexane/EtOH, 1 mL/min, λ 203 nm. Purification by flash chromatography on silica gel by eluting with EtOAc/Hexanes furnish the major diastereoisomer (97% de). Mp 120-122°C. R*f* (40% EtOAc/Hexane) 0.50. IR (cm^{-1}): 2970, 1678, 1635, 1512, 1438, 1406, 1249, 1180, 1124, 1031, 837, 748. ^1H NMR (300 MHz, $CDCl_3$) of the major diastereoisomer: δ = 1.75-1.83 (m, 4 H, 2 CH_2), 2.07 (dd, *J* = 7.3, 16.4 Hz, 1 H, CH_2), 2.34 (dd, *J* = 5.0, 16.4 Hz, 1 H, CH_2), 2.61-2.72 (m, 1 H, CH_2), 2.78-2.91 (m, 1 H, CH_2), 3.34-3.49 (m, 4 H, 2 CH_2), 3.78 (s, 3 H, OCH_3), 5.38 (dd, *J* = 5.1, 7.3 Hz, 1 H, CH), 5.72-5.83 (m, 1 H, NCH), 6.83 (d, *J* = 8.7 Hz, 2

H, H_{arom}), 7.29-7.49 (m, 5 H, H_{arom}), 7.85-7.88 (m, 1 H, H_{arom}). ^{13}C NMR (75 MHz, $CDCl_3$) of the major diastereoisomer: **C** 168.7 (CO), 167.8 (CO), 158.7, 146.6, 134.0, 131.8, **CH** 131.6, 128.6 (3 x CH), 128.2, 123.6, 122.7, 113.6 (2 x CH), 55.1, 48.3, **CH_2** 46.2, 45.7, 39.7, 25.7, 24.3, **CH_3** 55.3, 16.6. HRMS (ESI+) m/z calcd for $C_{23}H_{27}O_3N_2$ $[MH]^+$ 379.20162, found 379.20224.

(2R,3S)-2-(1-(4-Methoxyphenyl)ethyl)-3-(2-(morpholin-4-y)l-2-oxo-ethyl))-2,3-dihydroisoindol-1-one ((*R*)-**4b**)

Yield 80% (25.2 mg); 75% de Purification by flash chromatography on silica gel by eluting with EtOAc/Hexanes (3/7) and crystallization from hexanes/toluene furnish the major diastereoisomer (yield 63%) with 100% de by HPLC using Whelk01 CSP, at 25°C, with (75/25) *n*-hexane/EtOH, 0.8 mL/min, λ 202 nm; or using IA CSP, at 25°C, with (9/1) *n*-hexane/iPrOH, 1 mL/min, λ 202 nm. Mp 144-146°C. R*f* (40% EtOAc/Hexane) 0.54; IR (cm^{-1}): 2987, 1670, 1645, 1633, 1514, 1400, 1249, 1184, 1116, 1029, 850. ^1H NMR (300 MHz, $CDCl_3$) of the major diastereoisomer : δ = 1.76 (d, *J* = 7.3 Hz, 3 H, CH_3), 2.03 (dd, *J* = 8.3, 16.6 Hz, 1 H, CH_2), 2.35 (dd, *J* = 4.0, 16.6 Hz, 1 H, CH_2), 2.76-2.85 (m, 1 H, CH_2), 2.88-2.95 (m, 1 H, CH_2), 3.40 (t, *J* = 4.8 Hz, 2 H, CH_2), 3.54-3.65 (m, 4 H, 2 CH_2), 3.79 (s, 3 H, OCH_3), 5.37 (dd, *J* = 4.0, 8.3 Hz, 1 H, CH), 5.69-5.83 (m, 1 H, CH), 6.84 (d, *J* = 8.6 Hz, 2 H, H_{arom}), 7.38 (d, *J* = 8.6 Hz, 2 H, H_{arom}), 7.43-7.50 (m, 3 H, H_{arom}), 7.84-7.90 (m, 1 H, H_{arom}). ^{13}C NMR (75 MHz, $CDCl_3$) of the major diastereoisomer: **C** 168.6 (CO), 168.3 (CO), 158.9, 146.5, 133.8, **CH** 131.7, 128.6 (2 x CH), 128.3, 123.6, 123.0, 113.7 (2 x CH), 55.1, 48.4, **CH_2** 45.4, 41.9 (2 x CH_2), 37.5 (2 x CH_2), **CH_3** 55.3, 16.9. HRMS (ESI+) m/z calcd for $C_{23}H_{27}O_4N_2$ $[MH]^+$ 395.19653, found 395.19739.

(2R,3S)-N,N-Diisopropyl-2-[2-(1-(4-methoxyphenyl)ethyl)-3-oxo-2,3-dihydro-1H-isoindol-1-yl]acetamide ((*R*)-**4c**)

Yield 85% (27.7 mg); 65% de by NMR. Purification by flash chromatography on silica gel by eluting with EtOAc/Hexanes (3/7) and crystallization from hexanes/toluene furnish the major diastereoisomer (yield 65%) with 98% de by HPLC using C18 Grav-1 CSP (Macherey), at 25°C, with (60/40) H_2O/CH_3CN, 1 mL/min, λ 197 nm. Mp 104-106°C. R*f* (40% EtOAc/Hexane) 0.60. IR (cm^{-1}): 2968, 1683, 1635, 1512, 1471, 1249, 1180, 1041, 1033, 837, 758. ^1H NMR (300 MHz, $CDCl_3$) of the major diastereoisomer: δ = 0.90 (dd, *J* = 2.1, 6.7 Hz,

3 H, CH_3), 1.38 (dd, *J* = 2.4, 6.7 Hz, 3 H, CH_3), 1.76 (d, *J* = 7.3 Hz, 3 H, CH_3), 1.93 (dd, *J* = 9.5, 16.1 Hz, 1 H, CH_2Ph), 2.39 (dd, *J* = 3.6, 16.1 Hz, 1 H, CH_2Ph), 3.26-3.37 (m, 1 H, NCH), 3.52-3.65 (m, 1 H, NCH), 3.77 (s, 3 H, OCH_3), 5.37 (dd, *J* = 3.6, 9.4 Hz, 1 H, CH), 5.65-5.73 (m, 1 H, CH), 6.84 (d, *J* = 8.6 Hz, 2 H, H_{arom}), 7.38-7.52 (m, 5 H, H_{arom}), 7.82-7.87 (m, 1 H, H_{arom}). ^{13}C NMR (75 MHz, $CDCl_3$) of the major diastereoisomer: **C** 168.6 (CO), 168.5 (CO), 158.9, 146.8, 134.1, 131.8, **CH** 131.6, 128.5 (2 x CH), 128.1, 123.5, 123.4, 113.8 (2 x CH), 56.0, 48.9, 47.6, 45.8, **CH₂** 39.5, **CH₃** 55.3, 20.8 (2 x CH), 20.7, 20.5, 17.2. HRMS (ESI+) m/z calcd for $C_{25}H_{33}O_3N_2$ [MH]$^+$ 409.24857, found 409.24896.

(2R,3S)-N-Benzyl-2-(2-(1-(4-methoxyphenyl)ethyl)-3-oxoisoindolin-1-yl)acetamide ((*R*)-**4d**)

Yield 83% (27.5 mg); 48% de by HPLC using IA CSP, at 25°C, with (9/1) n-hexane/EtOH, 1 mL/min, λ 203 nm. R*f* (40% EtOAc/Hexane) 0.36. IR (cm^{-1}): 3310, 2912, 1675, 1656, 1508, 1445, 1320, 1218, 1152, 1098, 1012, 835, 750. ^{1}H NMR (300 MHz, $CDCl_3$) of the major diastereoisomer: δ = 1.75 (d, *J* = 7.3 Hz, 3 H, CH_3), 1.84 (dd, *J* = 8.6, 14.9 Hz, 1 H, CH_2CO), 2.48 (dd, *J* = 4.5, 14.9 Hz, 1 H, CH_2CO), 3.74 (s, 3 H, OCH_3), 4.22 (dd, *J* = 5.4, 14.6 Hz, 1 H, NCH_2Ph), 4.42 (dd, *J* = 6.1, 14.6 Hz, 1 H, NCH_2Ph), 5.21 (dd, *J* = 4.5, 8.6 Hz, 1 H, NCH), 5.55 (br t, 1 H, NH), 5.63 (q, *J* = 7.3 Hz, 1 H, NCH), 6.77 (d, *J* = 8.8 Hz, 2 H, H_{arom}), 7.15-7.43 (m, 10 H, H_{arom}), 7.77-7.80 (m, 1 H, H_{arom}). ^{13}C NMR (75 MHz, $CDCl_3$) of the major diastereoisomer: **C** 169.0 (2 x CO), 158.8, 145.5, 145.4, 137.7, **CH** 131.7, 128.7 (2 x CH), 128.4, 128.3 (2 x CH), 128.0 (2 x CH), 127.7, 123.7, 122.8, 113.8 (2 x CH), 55.6, 48.7, **CH₂** 43.7, 40.8, **CH₃** 55.2, 17.0. HRMS (ESI+) m/z calcd for $C_{26}H_{27}O_3N_2$ [MH]$^+$ 415.20162, found 415.20276.

(2R,3S)-N,N-Dicyclohexyl-2-[2-(1-(4-methoxyphenyl)ethyl)-3-oxo-2,3-dihydro-1H-isoindol-1-yl]acetamide ((*R*)-**4e**)

Yield 79% (30.8 mg); 67% de by HPLC using C18 Grav-1 CSP (Macherey), at 25°C, with (40/60) H_2O/CH_3CN, 1 mL/min, λ 198 nm. R*f* (40% EtOAc/Hexane) 0.55. IR (cm^{-1}): 2929, 1687, 1637, 1512, 1301, 1249, 1180, 846, 754. ^{1}H NMR (300 MHz, $CDCl_3$) of the major diastereoisomer: δ = 0.78-1.95 (m, 21 H), 2.28-2.51 (m, 2 H, NCH + 1 H NCH_2), 2.68-2.75 (m, 1 H, NCH), 3.77 (s, 3 H, OCH_3), 5.30 (dd, *J* = 3.4, 9.9 Hz, 1 H, NCH), 5.81 (m, 1 H, CH), 6.84 (d, *J* = 8.6 Hz, 2 H, H_{arom}), 7.32-7.53 (m, 5 H, H_{arom}), 7.83-7.87 (m, 1 H, H_{arom}). ^{13}C

NMR (75 MHz, $CDCl_3$) of the major diastereoisomer: **C** 168.7 (2 x CO), 158.9, 139.9, 134.4, 131.8, **CH** 131.6, 128.4 (2 x CH), 128.1, 123.7, 123.5, 113.7 (2 x CH), 55.8, 55.3, 52.7, 48.2, **CH_2** 31.1, 30.4, 30.1, 26.6, 25.4, 25.1, **CH_3** 55.1, 17.0. HRMS (ESI+) m/z calcd for $C_{31}H_{41}O_3N_2$ $[MH]^+$ 489.31117, found 489.31213.

(2R,3S)-5,6-Dimethoxy-2-(1-(4-methoxyphenyl)ethyl)-3-(2-oxo-2-(pyrrolidin-1-yl)ethyl))-2,3-dihydroisoindol-1-one ((*R*)-**5**)

Yield 78% (27.3 mg); 70% de. Purification by flash chromatography on silica gel by eluting with EtOAc/Hexanes (3/7) and crystallization from hexanes/toluene furnish the major diastereoisomer (yield 63%) with 98% de by HPLC using IA CSP, at 25 °C, with (8/2) n-hexane/EtOH, 1 mL/min, λ 217 nm. Mp 152-154°C. R*f* (40% EtOAc/Hexane) 0.42. IR (cm^{-1}): 2968, 1654, 1618, 1508, 1446, 1400, 1301, 1220, 1178, 1020, 846, 777. ^1H NMR (300 MHz, $CDCl_3$) of the major diastereoisomer: δ = 1.75-1.94 (m, 7 H, 2 CH_2 + CH_3), 1.98 (dd, *J* = 8.0, 16.3 Hz, 1 H, CH_2), 2.32 (dd, *J* = 4.8, 16.3 Hz, 1 H, CH_2), 2.67-2.73 (m, 1 H, CH_2), 2.86-2.94 (m, 1 H, CH_2), 3.39-3.44 (m, 2 H, CH_2), 3.78 (s, 3 H, OCH_3), 3.88 (s, 3 H, OCH_3), 3.94 (s, 3 H, OCH_3), 5.24 (dd, *J* = 4.8, 7.9 Hz, 1 H, CH), 5.68-5.76 (m, 1 H, NCH), 6.83 (d, *J* = 8.7 Hz, 2 H, H_{arom}), 7.01 (s, 1 H, H_{arom}), 7.33 (s, 1 H, H_{arom}), 7.39 (d, *J* = 8.7 Hz, 2 H, H_{arom}). ^{13}C NMR (75 MHz, $CDCl_3$) of the major diastereoisomer: **C** 168.8 (CO), 168.2 (CO), 158.7, 152.6, 149.8, 140.3, 134.1, 124.0, **CH** 128.5 (2 x CH), 113.6 (2 x CH), 105.4, 105.2, 55.2, 48.4, **CH_2** 46.2, 45.6, 39.8, 25.8, 24.3, **CH_3** 56.2 (2 x OCH_3), 54.7, 16.8. HRMS (ESI+) m/z calcd for $C_{25}H_{31}O_5N_2$ $[MH]^+$ 439.22275, found 439.22339.

2.4. *General procedure for the synthesis of NH isoindolinone* **1,2**

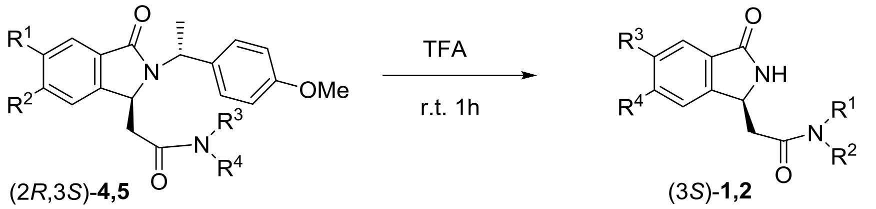

(2*R*,3*S*)-**4,5** (3*S*)-**1,2**

A solution of isoindolinone (*R*)-**4a–c,e** and (*R*)-**5** (0.15 mmol) and trifluoroacetic acid (1 mL) was stirred at room temperature for 1 h. The reaction mixture was concentrated and the residue was diluted with EtOAc (5 mL) and washed with saturated aqueous $NaHCO_3$ solution (5 mL) and water (5 mL). The organic solution was dried ($MgSO_4$), filtered and concentrated

under vacuum. The oily residue was purified by flash column chromatography on silica gel with acetone/hexanes (50:50) as eluant to yield the expected product (*S*)-**1a–c,e** and (*S*)-**2**. The enantiomeric excess for compounds (*S*)-**1a–c,e** and (*S*)-**2** was determined by ^{1}H NMR and/or HPLC.

(3S)-3-(2-Oxo-2-(pyrrolidin-1-yl)ethyl)-2,3-dihydroisoindol-1-one (*S*)-**1a**)

Yield 87% (31.8 mg). Mp 64-65 °C. 82 % E.e. by HPLC using Whelk01 CSP, at 25°C, with (75/25) n-hexane/EtOH, 0.8 mL/min, λ 201 nm. R*f* (60% Acetone/Hexane) 0.45. $[\alpha]_D^{20}$ -75.5 (*c* 0.88, $CHCl_3$). IR (cm^{-1}): 3255, 2970, 1701, 1685, 1631, 1618, 1444, 1136, 748. ^{1}H NMR (300 MHz, $CDCl_3$): δ = 1.81-1.98 (m, 4 H, 2 CH_2), 2.42 (dd, *J* = 10.3, 16.4 Hz, 1 H, CH_2Ph), 2.95 (dd, *J* = 3.7, 16.4 Hz, 1 H, CH_2Ph), 3.32-3.38 (m, 2 H, CH_2), 3.49-3.55 (m, 2 H, CH_2), 5.04 (dd, *J* = 3.6, 10.3 Hz, 1 H, CH), 7.37-7.59 (m, 4 H, 3 H_{arom} + NH), 7.84 (d, *J* = 7.4 Hz, 1 H, H_{arom}). ^{13}C NMR (75 MHz, $CDCl_3$): **C** 169.8 (CO), 168.5 (CO), 146.7, 132.2, **CH** 131.7, 128.3, 123.8, 122.5, 53.3, **CH_2** 46.5, 45.8, 40.0, 25.9, 24.3. HRMS (ESI+) m/z calcd for $C_{14}H_{17}O_2N_2$ $[MH]^+$ 245.12845, found 245.12897.

(3S)-3-(2-Morpholino-2-oxoethyl)-2,3-dihydroisoindol-1-one (*S*)-**1b**)

Yield 91% (35.5 mg). Mp 46-47 °C. 98 % E.e. by HPLC using Whelk01 CSP, at 25°C, with (70/30) n-hexane/EtOH, 0.5 mL/min, λ 202 nm. R*f* (60% Acetone/Hexane) 0.48. $[\alpha]_D^{20}$ -91.6 (*c* 0.52, $CHCl_3$). IR (cm^{-1}): 3219, 2922, 1681, 1631, 1469, 1238, 1201, 1112, 1035, 846, 748. ^{1}H NMR (300 MHz, $CDCl_3$): δ = 2.43 (dd, *J* = 10.3, 16.5 Hz, 1 H, CH_2Ph), 2.98 (dd, *J* = 3.2, 16.4 Hz, 1 H, CH_2Ph), 3.42-3.48 (m, 2 H, CH_2), 3.61-3.72 (m, 6 H, 3 x CH_2), 5.06 (br d, *J* = 9.5 Hz, 1 H, CH), 7.29-7.57 (m, 4 H, 3 H_{arom} + NH), 7.85 (d, *J* = 7.6 Hz, 1 H, H_{arom}). ^{13}C NMR (75 MHz, $CDCl_3$): **C** 170.3 (CO), 168.9 (CO), 146.5, 132.0, **CH** 131.9, 128.5, 124.2, 122.4, 53.4, **CH_2** 66.7, 66.3, 45.7, 42,1, 38.6. HRMS (ESI+) m/z calcd for $C_{14}H_{17}O_3N_2$ $[MH]^+$ 261.12337, found 261.12387.

(3S)-N,N-Diisopropyl-2-(3-oxoisoindolin-1-yl)acetamide (*S*)-**1c**)

Yield 85% (34.9 mg); viscous oil. 97 % E.e. by HPLC using Whelk01 CSP, at 25°C, with (60/40) n-hexane/EtOH, 0.5 mL/min, λ 202 nm. R*f* (60% Acetone/Hexane) 0.50. $[\alpha]_D^{20}$ -61.3

(c 0.70, $CHCl_3$). IR (cm^{-1}): 3226, 2930, 1689, 1627, 1446, 1388, 1149, 758, 698. ^1H NMR (300 MHz, $CDCl_3$): δ = 1.15 (d, J = 6.7 Hz, 3 H, CH_3), 1.22 (d, J = 6.7 Hz, 3 H, CH_3), 1.42 (d, J = 7.0 Hz, 3 H, CH_3), 1.44 (d, J = 7.0 Hz, 3 H, CH_3), 2.33 (dd, J = 10.7, 16.4 Hz, 1 H, CH_2Ph), 3.02 (dd, J = 3.1, 16.4 Hz, 1 H, CH_2Ph), 3.47-3.52 (m, 1 H, NCH), 3.78-3.91 (m, 1 H, NCH), 5.03 (dd, J = 2.7, 10.7 Hz, 1 H, CH), 6.98 (brs, 1 H, NH), 7.27-7.57 (m, 3 H, 3 H_{arom}), 7.87 (d, J = 7.4 Hz, 1 H, H_{arom}). ^{13}C NMR (75 MHz, $CDCl_3$): **C** 169.7 (CO), 168.7 (CO), 146.7, 132.4, **CH** 131.7, 128.4, 124.1, 122.3, 53.5, 48.5, 46.0, **CH_2** 40.7, **CH_3** 20.8, 20.7, 20.6, 20.5. HRMS (ESI+) m/z calcd for $C_{16}H_{23}O_2N_2$ $[MH]^+$ 275.17540, found 275.17584.

(3S)-N,N-Dicyclohexyl-2-(3-oxoisoindolin-1-yl)acetamide (*S*)-**1e**)

Yield 90% (47.8 mg). Mp 108-109 °C. 67% E.e. by HPLC using Whelk01 CSP, at 25°C, with (70/30) n-hexane/EtOH, 0.5 mL/min, λ 200 nm. Mp 170-171°C. R*f* (60% Acetone/Hexane) 0.46. $[\alpha]_D^{20}$ -48.2 (c 0.36, $CHCl_3$). IR (cm^{-1}): 3259, 2924, 1703, 1635, 1359, 1143, 997, 893, 744. ^1H NMR (300 MHz, $CDCl_3$): δ = 1.05-1.92 (m, 20 H), 2.31 (dd, J = 10.5, 16.3 Hz, 1 H, CH_2Ph), 2.32-2.51 (br s, 1 H, NCH), 3.02 (dd, J = 3.1, 16.3 Hz, 1 H, CH_2Ph), 3.28-3.36 (m, 1 H, NCH), 5.02 (br d, J = 10.5 Hz, 1 H, CH), 7.04 (brs, 1 H, NH), 7.46-7.60 (m, 3 H, 3 H_{arom}), 7.86 (d, J = 7.2 Hz, 1 H, H_{arom}). ^{13}C NMR (75 MHz, $CDCl_3$): **C** 169.7 (CO), 168.9 (CO), 146.8, 132.4, **CH** 131.7, 128.3, 124.0, 122.4, 57.8, 56.2, 53.6, **CH_2** 40.8, 31.1, 31.0, 30.2, 29.9, 26.6, 26.55, 25.9, 25.8, 25.4, 25.1. HRMS (ESI+) m/z calcd for $C_{22}H_{31}O_2N_2$ $[MH]^+$ 355.23800, found 355.23856.

(3S)-5,6-Dimethoxy-3-(2-oxo-2-(pyrrolidin-1-yl)ethyl)isoindolinone (*S*)-**2**)

Yield 91% (41.5 mg); Mp 102-103 °C; 95% Ee by HPLC using Whelk01 CSP, at 25°C, with (60/40) n-hexane/EtOH, 0.5 mL/min, λ 217 nm. R*f* (80% Acetone/Hexane) 0.45. $[\alpha]_D^{20}$ -54.3 (c 0.60, $CHCl_3$). IR (cm^{-1}): 3290, 2931, 1683, 1616, 1498, 1454, 1292, 1217, 1076. ^1H NMR (300 MHz, $CDCl_3$): δ = 1.78-1.94 (m, 4 H, 2 CH_2), 2.30 (dd, J = 10.4, 16.4 Hz, 1 H, CH_2Ph), 2.82 (dd, J = 3.7, 16.4 Hz, 1 H, CH_2Ph), 3.24-3.35 (m, 2 H, CH_2), 3.42-3.48 (m, 2 H, CH_2), 3.86 (s, 3 H, OCH_3), 3.88 (s, 3 H, OCH_3), 4.89 (dd, J = 3.4, 10.1 Hz, 1 H, CH), 6.84 (s, 2 H, H_{arom}), 6.89 (brs, 1 H, NH), 7.24 (s, 2 H, H_{arom}). ^{13}C NMR (75 MHz, $CDCl_3$): **C** 170.1 (CO),

168.6 (CO), 152.8, 150.0, 140.3, 124.4, **CH** 105.5 (2 x CH), 104.6 (2 x CH), 52.9, **CH_2** 46.6, 45.8, 40.3, 26.0, 24.3, **CH_3** 56.3, 56.25.

2.5. General procedure for the synthesis of pazinaclone analogue **27**

*2.5.1. Synthesis of benzamide derivative **24**(first strategy)*

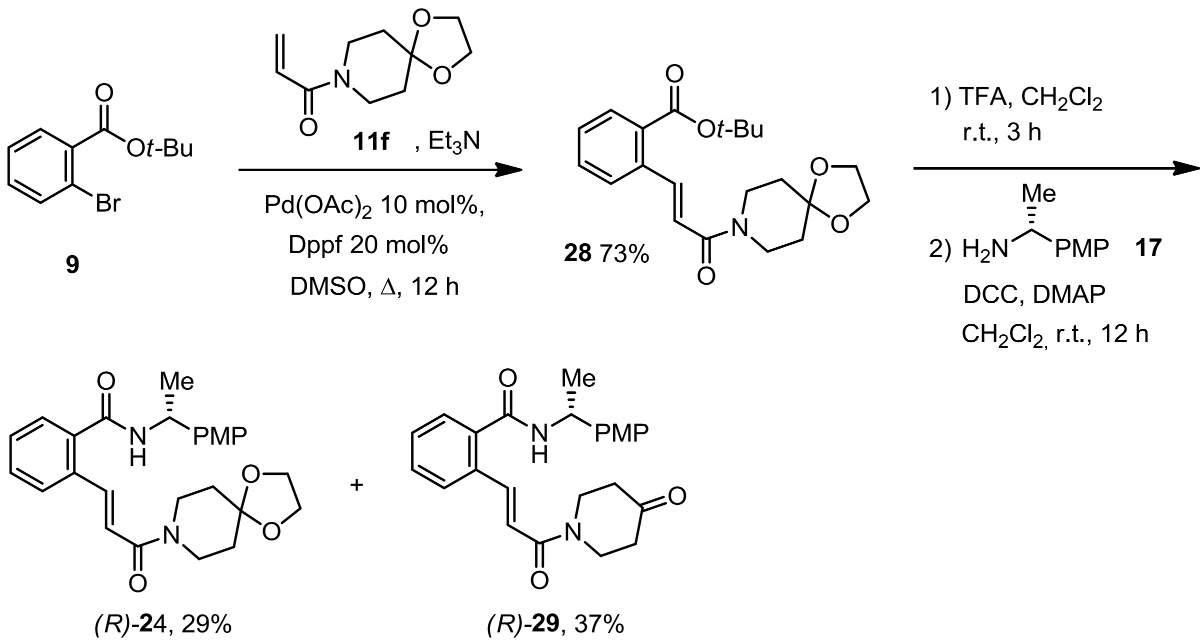

To a solution of 2-Bromobenzoic acid *tert*-butyl ester (**9**, 1 g, 3.9 mmol) in DMSO (20 mL) maintained under nitrogen atmosphere, were added $Pd(OAc)_2$ (88 mg, 10 mol%), dppf (431 mg, 20 mol %), Et_3N (2.7 mL, 19.5 mmol) and 1-(1,4-dioxa-8-azaspiro[4.5]dec-8-yl)propenone (**11f**, 1.53 g, 7.8 mmol). The mixture was stirred for 12 h at reflux, and then it was diluted with water (5 mL) and extracted with CH_2Cl_2 (3 × 50 mL). The combined organic layers were dried over $MgSO_4$ and concentrated under vacuum to give an oil which was purified by flash column chromatography on silica gel using EtOAc/hexanes (50:50) as eluent. Evaporation of solvents under vacuum afforded compound **28**.

2-[(E)-3-(1,4-Dioxa-8-azaspiro[4.5]dec-8-yl)-3-oxopropenyl]benzoic acid tert-butyl ester (**28**)

Yield: 73% (1.09 g). Mp 128-129 °C. ^1H NMR (300MHz, $CDCl_3$): δ = 1.61 (s, 9 H, 3 x CH_3), 1.72-1.78 (m, 4 H, 2 x CH_2), 3.66-3.79 (m, 4 H, 2 x NCH_2), 3.98 (s, 4 H, OCH_2CH_2O), 6.75

(d, J = 15.4 Hz, 1 H, =CH), 7.37 (td, J = 7.4-1.3 Hz, 1 H, H_{arom}), 7.48 (td, J = 7.6-1.4 Hz, 1 H, H_{arom}), 7.56 (d, J = 7.3 Hz, 1 H, H_{arom}), 7.86 (dd, J = 7.7-1.4 Hz, 1 H, H_{arom}), 8.22 (d, J = 15.4 Hz, 1 H, =CH). ^{13}C NMR (75 MHz, $CDCl_3$): **C** 166.2 (CO), 165.1 (CO), 136.6, 131.8, 106.7, 81.9, **CH** 141.8, 131.5, 130.3, 128.6, 127.6, 119.7, **CH_2** 64.3 (2 x CH_2), 43.7, 40.2, 35.7, 34.6, **CH_3** 20.0.

To a solution of ester **28** (747 mg, 2 mmol) in dry CH_2Cl_2 (10 mL) was added trifluoroacetic acid (1.54 mL, 20 mmol). The mixture was stirred at room temperature for 3 h. The mixture was concentrated to dryness, and the residue was washed successively with 3 × 30 mL of EtOAc to afford the corresponding acid which was used for the next step without further purification. To a stirred solution of benzoic acid (2 mmol) in anhydrous CH_2Cl_2 (20 mL) were added *N*,*N*'-dicylohexylcarbodiimide (412 mg, 2 mmol), *N*,*N*-(dimethylamino)pyridine (244 mg, 2 mmol) and (*R*)-4-methoxy-α-methylbenzylamine **17** (0.30 mL, 2 mmol) under an argon atmosphere. After stirring for 16 h, water (30 mL) was added and the aqueous layer was extracted with CH_2Cl_2 (2 × 20 mL). The organic layer was dried $MgSO_4$, filtered and concentrated. Flash chromatographic purification over silica using EtOAc/hexanes (80:20) afforded a mixture of benzamide derivatives **24** (29% yield) and **29** (37% yield).

N-((R)-1-(4-Methoxyphenyl)ethyl)-2-((E)-3-oxo-(1,4-dioxa-8-azaspiro[4.5]decan-8-yl)propen-1-yl)benzamide ((*R*)-**24**)

Yield 29% (261 mg). Mp 149-150°C. R*f* (100% EtOAc) 0.60. $[\alpha]_D^{20}$ + 20.4 (*c* 0.81, $CHCl_3$). IR (cm^{-1}): 3375, 2933, 1635, 1604, 1512, 1435, 1246, 1105, 1029, 948, 825, 761. ^{1}H NMR (300 MHz, $CDCl_3$): δ = 1.59 (d, J = 6.9 Hz, 3 H, CH_3), 1.64-1.72 (m, 4 H), 3.58-3.66 (m, 4 H), 3.79 (s, 3 H, OCH_3), 3.98 (s, 4 H, OCH_2CH_2O), 5.20-5.27 (m, 1 H, NCH), 6.19 (brd, J = 7.8 Hz, 1 H, NH), 6.72 (d, J = 15.6 Hz, 1 H, =CH), 6.83-6.88 (m, 2 H, H_{arom}), 7.28-7.52 (m, 6 H, H_{arom}), 7.74 (d, J = 15.6 Hz, 1 H, CH=). ^{13}C NMR (75 MHz, $CDCl_3$): **C** 167.9 (CO), 165.2 (CO), 158.9, 136.7, 135.0, 133.7, **CH** 139.4, 130.0, 129.0, 127.6, 127.5 (2 x CH), 121.4, 114.1 (2 x CH), 49.0, **CH_2** 64.5 (2 x CH_2), 43.9, 40.3, 35.8, 34.8, **CH_3** 55.3, 21.8. HRMS (ESI+) m/z calcd for $C_{26}H_{31}O_5N_2$ $[MH]^+$ 451.22725, found 451.22397.

N-[(R)-1-(4-Methoxyphenyl)ethyl]-2-[(E)-3-oxo-3-(4-oxopiperidin-1-yl)propenyl]benzamide ((*R*)-**29**)

Yield 37% (300 mg). Mp 62-63°C. $[\alpha]_D^{20}$ + 46.0 (*c* 0.57, $CHCl_3$). ^1H NMR (300 MHz, $CDCl_3$): δ = 1.59 (d, *J* = 6.9 Hz, 3 H, CH_3), 1.62-1.68 (m, 2 H, CH_2), 2.39-2.57 (m, 2 H, CH_2), 3.76-4.01 (m, 7 H, 2 x NCH_2 + OCH_3), 5.26 (m, 1 H, CH), 6.03 (d, *J* = 7.9 Hz, 1 H, NH), 6.76 (d, *J* = 15.6 Hz, 1 H, =CH), 6.88 (d, *J* = 8.7 Hz, 2 H, H_{arom}), 7.29-7.57 (m, 6 H, H_{arom}), 7.83 (d, *J* = 15.6 Hz, 1 H, =CH). ^{13}C NMR (75 MHz, $CDCl_3$): **C** 206.8 (CO), 168.0 (CO), 165.9 (CO), 159.0, 136.7, 134.9, 133.5, **CH** 140.3, 130.2, 129.3, 127.9, 127.7, 127.5 (2 x CH), 120.5, 114.2 (2 x CH), 49.1, **CH_2** 41.1 (2 x CH_2), 29.7 (2 x CH_2), **CH_3** 55.3, 21.9.

*2.5.2. Synthesis of benzamide derivative **24** (second strategy)*

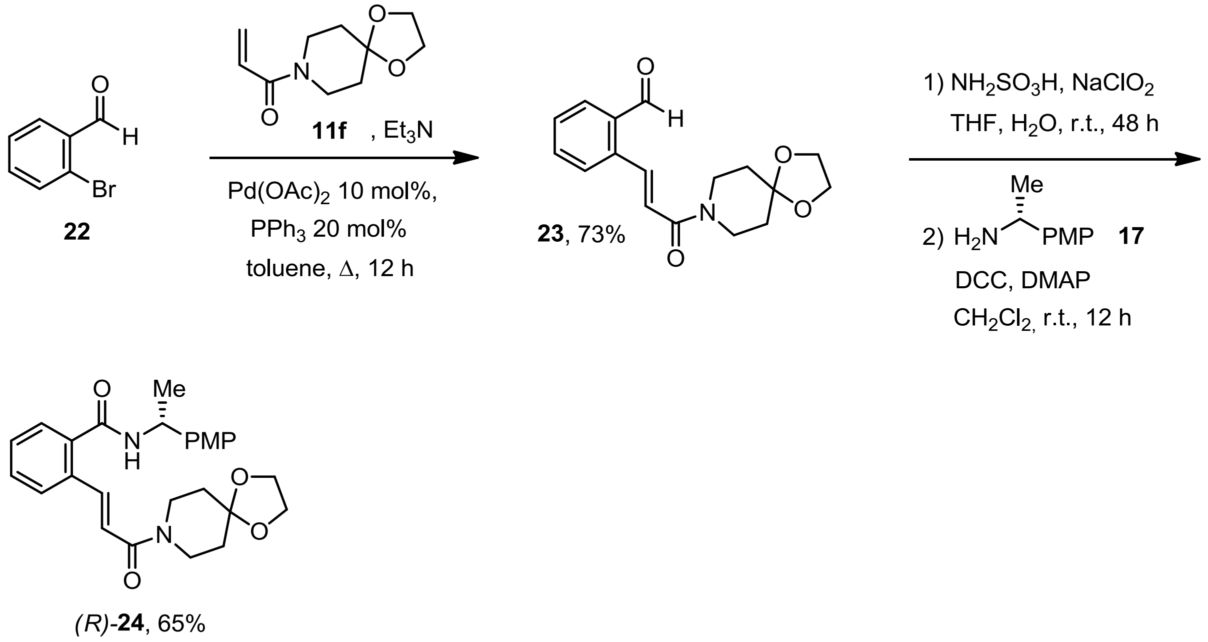

To a solution of 2-bromobenzaldehyde **22** (0.58 mL, 5 mmol) in toluene (10 mL) maintained under nitrogen atmosphere, were added $Pd(OAc)_2$ (56 mg, 5 mol %), PPh_3 (131 mg, 10 mol %), Et_3N (2.1 mL, 15 mmol) and 1-(1,4-dioxa-8-azaspiro[4.5]dec-8-yl)propenone (**11f**, 1.47 g, 7.5 mmol). The mixture was stirred for 12 h at reflux, and then it was diluted with water (5 mL) and extracted with CH_2Cl_2 (3 × 50 mL). The combined organic layers were dried over $MgSO_4$ and concentrated under vacuum to give an oil which was purified by flash column chromatography on silica gel using EtOAc/hexanes (80:20) as eluent. Evaporation of solvents under vacuum afforded compound **23**.

2-[(E)-3-(1,4-Dioxa-8-azaspiro[4.5]dec-8-yl)-3-oxopropenyl]benzaldehyde (**23**)

Yield 73% (1.098 g). Mp 142-143°C. ^{1}H NMR (300 MHz, $CDCl_3$): δ = 1.75-1.79 (m, 4 H, 2 x CH_2), 3.73-3.83 (m, 4 H, 2 x NCH_2), 4.01 (s, 4 H, OCH_2CH_2O), 6.78 (d, *J* = 15.4 Hz, 1 H, =CH), 7.49-7.62 (m, 3 H, H_{arom}), 7.90 (d, *J* = 7.5 Hz, 1 H, H_{arom}), 8.33 (d, *J* = 15.4 Hz, 1 H, =CH), 10.34 (s, 1 H, H_{CHO}); ^{13}C NMR (75 MHz, $CDCl_3$): **C** 164.8 (CO), 138.1, 133.8, 106.9, **CH** 191.6, 138.4, 133.9, 131.1, 129.3, 128.0, 123.2, **CH_2** 64.5 (2 x CH_2), 44.2, 40.4, 35.8, 34.8.

To a stirred solution of benzaldehyde **23** (723 mg, 2.4 mmol) in THF (5 mL) were added sodium chlorite 80% (675 mg, 7.5 mmol) and sulfamic acid (582 mg, 6 mmol dissolved in water 5 mL) under an argon atmosphere. The resulting mixture was stirred for 48 h at room temperature. Volatiles were removed *in vacuo* and the residue was dissolved in CH_2Cl_2. The organic layer was washed with saturated aqueous ammonium chloride (40 mL) and the aqueous layer was extracted with CH_2Cl_2 (80 mL). The organic layers were combined, dried over anhydrous $MgSO_4$, and concentrated in vacuo to afford the corresponding acid which was used for the next step without further purification. To a stirred solution of benzoic acid (634 mg, 2 mmol) in anhydrous CH_2Cl_2 (20 mL) were added *N*,*N*'-dicyclohexylcarbodiimide (412 mg, 2 mmol), *N*,*N*-(dimethylamino)pyridine (244 mg, 2 mmol) and (*R*)-4-methoxy-α-methylbenzylamine (0.30 mL, 2 mmol) under an argon atmosphere. After stirring for 16 h, water (30 mL) was added and the aqueous layer was extracted with CH_2Cl_2 (2 × 20 mL). The organic layer was dried $MgSO_4$, filtered and concentrated. Flash chromatographic purification over silica using EtOAc/hexanes (80:20) afforded benzamide derivative **24 (**Yield 65%, 702 mg).

2.5.3 *Synthesis of pazinaclone analogue* **27**

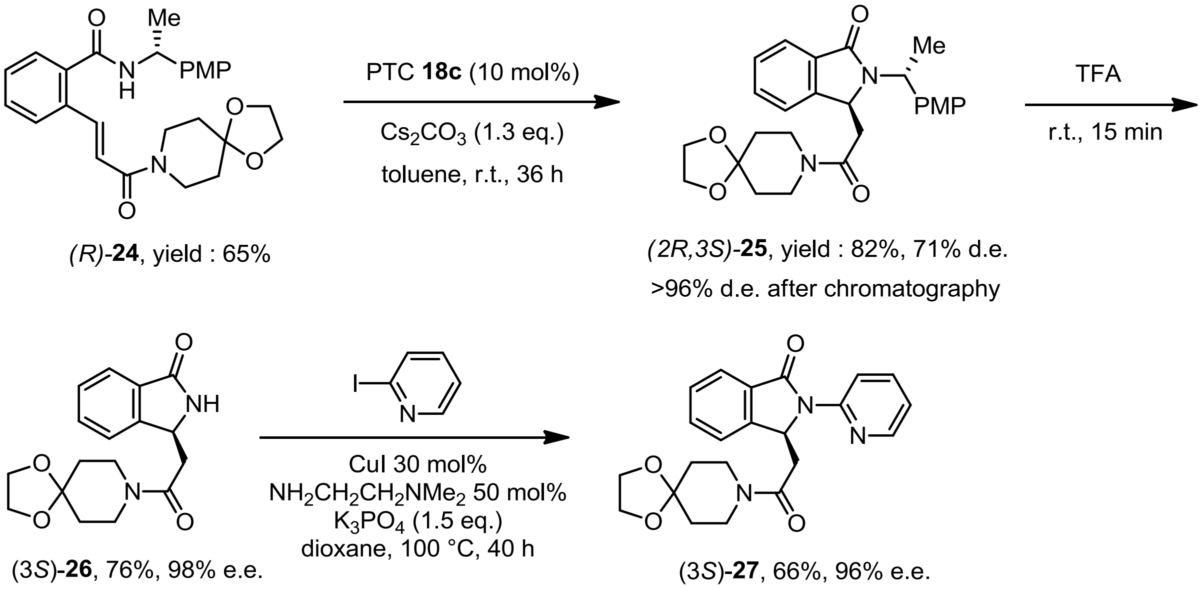

A mixture of benzamide **24** (90 mg, 0.20 mmol), cesium carbonate (84.7 mg, 0.26 mmol) and catalyst **18c** (10.4 mg, 10 mol %) was stirred for 16 h in toluene (1 mL) at room temperature. The resulting reaction mixture was monitored by TLC until completion. The crude product was purified by flash chromatography on silica gel by eluting with EtOAc/hexanes (80:20) to afford after evaporation of solvents under vacuum product **25.**

(2R,3S)-2-(1-4-Methoxyphenyl)ethyl)-3-(2-oxo-2-(1,4-dioxa-8-azaspiro[4.5]decan-8-yl)ethyl-2,3-dihydroisoindol-1-one ((*R*)-**25**)

Yield 82% (73.8 mg, 71% de); Purification by flash chromatography on silica gel by eluting with EtOAc/Hexanes (2/8) and crystallization from hexanes/toluene furnish the major diastereoisomer with 98% de (yield 70%) by HPLC using IA CSP, at 25 °C, with (9/1) n-hexane/EtOH, 1 mL/min, λ 201 nm; or using Whelk01 CSP, at 25 °C, with (75/25) n-hexane/EtOH, 0.8 mL/min, λ 201 nm.. Mp 59-60°C. R*f* (40% EtOAc/Hexane) 0.58. $[\alpha]_D^{20}$ + 154.9 (*c* 0.25, $CHCl_3$). IR (cm^{-1}): 2931, 1681, 1641, 1512, 1467, 1400, 1247, 1180, 1099, 1031, 945, 835, 758. ^1H NMR (300 MHz, $CDCl_3$) of the major diastereoisomer: δ = 1.38-1.41 (m, 2 H, CH_2), 1.63-1.67 (m, 2 H, CH_2), 1.76 (d, J = 7.2 Hz, 3 H, CH_3), 2.04 (dd, J = 8.6, 16.6 Hz, 1 H, CH_2), 2.38 (dd, J = 3.9, 16.6 Hz, 1 H, CH_2), 2.91-3.06 (m, 2 H, NCH_2), 3.65-3.68 (m, 2 H, NCH_2), 3.79 (s, 3 H, OCH_3), 3.92-3.96 (m, 4 H, OCH_2CH_2O), 5.35 (dd, J = 3.8, 8.6 Hz, 1 H, NCH), 5.73-5.81 (m, 1 H, CH), 6.85 (d, J = 8.8 Hz, 2 H, H_{arom}), 7.37-7.50 (m, 5 H, H_{arom}), 7.85-7.87 (m, 1 H, H_{arom}); ^{13}C NMR (75 MHz, $CDCl_3$) of the major diastereoisomer: **C**

168.6 (CO), 167.9 (CO), 158.9, 146.7, 133.9, 106.6, **CH** 131.7, 128.6 (2 x CH), 128.3, 123.6, 123.1, 113.7 (2 x CH), 55.3, 48.4, **CH_2** 64.5 (2 x CH_2), 43.1, 39.8, 37.5, 35.2, 34.7, **CH_3** 55.4, 16.9. HRMS (ESI+) m/z calcd for $C_{26}H_{31}O_5N_2$ $[MH]^+$ 451.22275, found 451.22394.

A solution of isoindolinone **25** (67.5 mg, 0.15 mmol) in TFA (1 mL) was stirred at room temperature for 15 min. After which time EtOAc (2 mL) was added and the resulting mixture was concentrated under reduced pressure. This operation was repeated three times and the oily residue was purified by flash column chromatography on silica gel with Acetone/Hexanes (50:50) as eluant to yield the expected product **26**.

(3S)-3-(2-oxo-2-(1,4-dioxa-8-azaspiro[4.5]decan-8-yl)ethyl)-2,3-dihydro-isoindol-1-one ((*S*)-**26**)

Yield 76% (38.7 mg); 98% E.e by HPLC using Whelk01 CSP, at 25°C, with (75/25) n-hexane/EtOH, 0.8 mL/min, λ 202 nm. Mp 154-155°C. R*f* (50% acetone/hexane) 0.48. $[\alpha]_D^{20}$ -39.5 (*c* 0.60, $CHCl_3$). IR (cm^{-1}): 3406, 2900, 1705, 1625, 1473, 1450, 1226, 1112, 947, 896, 748. ^1H NMR (300 MHz, $CDCl_3$): δ = 1.67-1.75 (m, 4 H), 2.42 (dd, *J* = 10.6, 16.5 Hz, 1 H, CH_2Ph), 3.07 (dd, *J* = 3.2, 16.5 Hz, 1 H, CH_2Ph), 3.46-3.49 (m, 2 H, CH_2), 3.65-3.82 (m, 2 H), 3.94-4.03 (m, 4 H, OCH_2CH_2O), 5.03 (dd, *J* = 2.6, 10.5 Hz, 1 H, NCH), 7.09 (br s, 1 H, NH), 7.46-7.58 (m, 3 H, 3 H_{arom}), 7.87 (d, *J* = 7.5 Hz, 1 H, H_{arom}); ^{13}C NMR (75 MHz, $CDCl_3$): **C** 170.3 (CO), 168.9 (CO), 146.5, 132.0, **CH** 131.9, 128.5, 124.2, 122.4, 53.4, **CH_2** 66.7, 66.3, 45.7, 42,1, 38.6. HRMS (ESI+) m/z calcd for $C_{17}H_{21}O_4N_2$ $[MH]^+$ 317.14958, found 317.15009.

A mixture of isoindolinone **26** (20 mg, 0.063 mmol), 2-iodopyridine (7.4 μL, 0.069 mmol), potassium phosphate tribasic (20 mg, 0.095 mmol), CuI (3.6 mg, 30 mol %) and *N,N*-dimethylethylenediamine (3.5 μL, 50 mol %, distilled) was stirred for 40 h in dioxane (1 mL, distilled) at 100 °C under nitrogen. Afterwards, the resulting reaction mixture was cooled at room temperature and subsequently filtered through a pad of celite using dioxane (4 mL). After evaporation of solvent under vacuum, the crude product was purified by preparative TLC on silica gel by eluting with acetone/hexanes (4:6). The target product was then

recovered and extracted with acetone; evaporation of solvent and drying under vacuum leading finally to product **27.**

(S)-3-(2-oxo-2-(1,4-dioxa-8-azaspiro[4.5]decan-8-yl)ethyl)-2-(pyridin-2-yl)isoindolin-1-one ((*S*)-**27**)

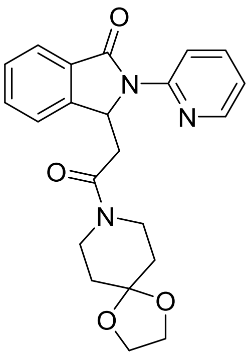

Yield: 66% (17 mg, 96% E.e by HPLC using AD CSP, at 25°C, with (70/30) n-hexane/EtOH, 1 mL/min, λ 202 nm. No racemization was observed by HPLC after heating a sample in DMF at 150°C for 48 hours (Sealed tube). $[\alpha]_D^{20}$ +132.4 (*c* 0.33, CH_2Cl_2). ^{1}H NMR (300MHz, $CDCl_3$): δ = 1.57 (m, 2 H), 1.72 (m, 2 H), 2.42 (dd, *J* = 9.6-10.2 Hz, 1 H), 3.47 (t, *J* = 5.7 Hz, 1 H), 3.56 (dd, *J* = 15.6-3.0 Hz, 1 H), 3.79 (t, *J* = 6.0 Hz, 2 H), 3.96 (d, *J* = 3.0 Hz, 4 H), 6.11 (dd, *J* = 9.6-3.0 Hz, 1 H, H_{arom}), 7.07 (bt, *J* = 6.3 Hz, 1 H, H_{arom}), 7.49 (t, *J* = 7.4 Hz, 1 H, H_{arom}), 7.58 (t, *J* = 7.5 Hz, 1 H, H_{arom}), 7.70 (d, *J* = 7.8 Hz, 1 H, H_{arom}), 7.77 (t, *J* = 7.9 Hz, 1 H, H_{arom}), 7.91 (d, *J* = 7.2 Hz, 1 H, H_{arom}), 8.41 (bs, 1H, H_{arom}), 8.61 (d, *J* = 8.2 Hz, 1 H, H_{arom}). ^{13}C NMR (75 MHz, $CDCl_3$): **C** 168.6 (CO), 167.9 (CO), 151.2, 146.1, 131.7, 106.9, **CH** 147.9, 138.1, 133.1, 128.7, 124.2, 124.0, 119.6, 115.7, 57.4, **CH_2** 64.6 (2 x CH_2), 43.7, 40.1, 37.6, 35.7, 34.9. HRMS (ESI+) m/z calcd for $C_{22}H_{24}O_4N_3$ $[MH]^+$ 394.17613, found 394.17184.

3. **References**

1) R. L. Wiseman, M. S. Kelker, T. Foss, I. A. Wilson, J. W. Kelly, *J. Am. Chem. Soc.* **2005**, *127*, 5540.

2) T. L. Gilchrist, R. Mendonca, *Arkivoc* **2000** (V), 769.

4. ^{1}H and ^{13}C spectra for compounds

2-Bromo-4,5-dimethoxybenzoic acid tert-butyl ester (**10**)

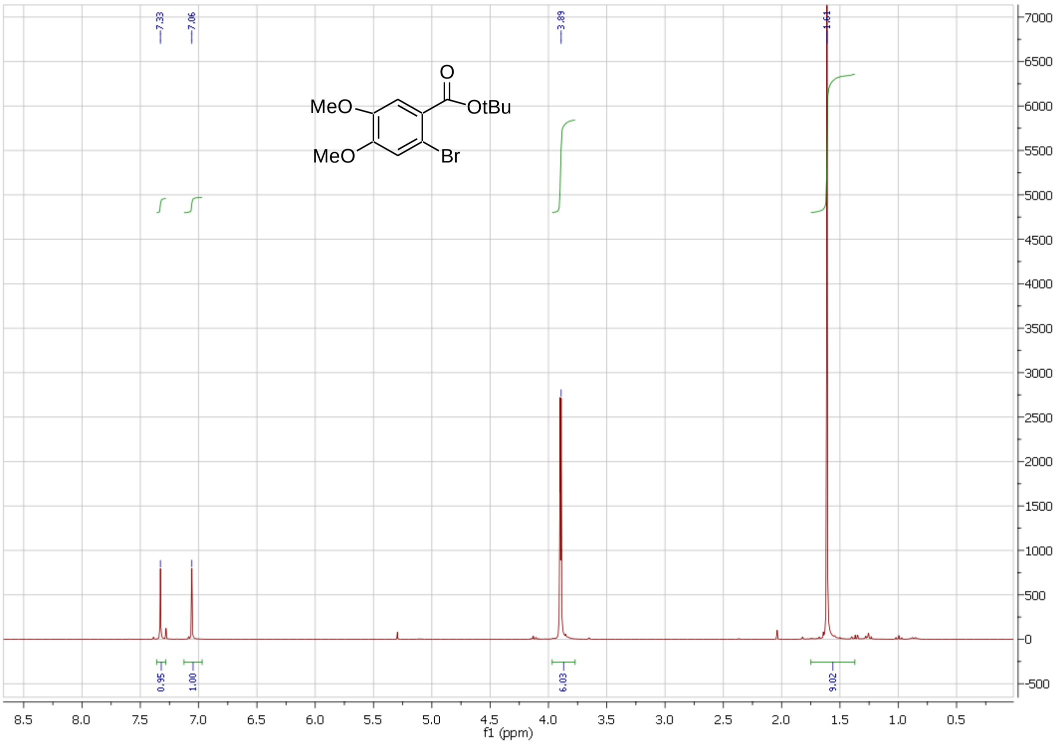

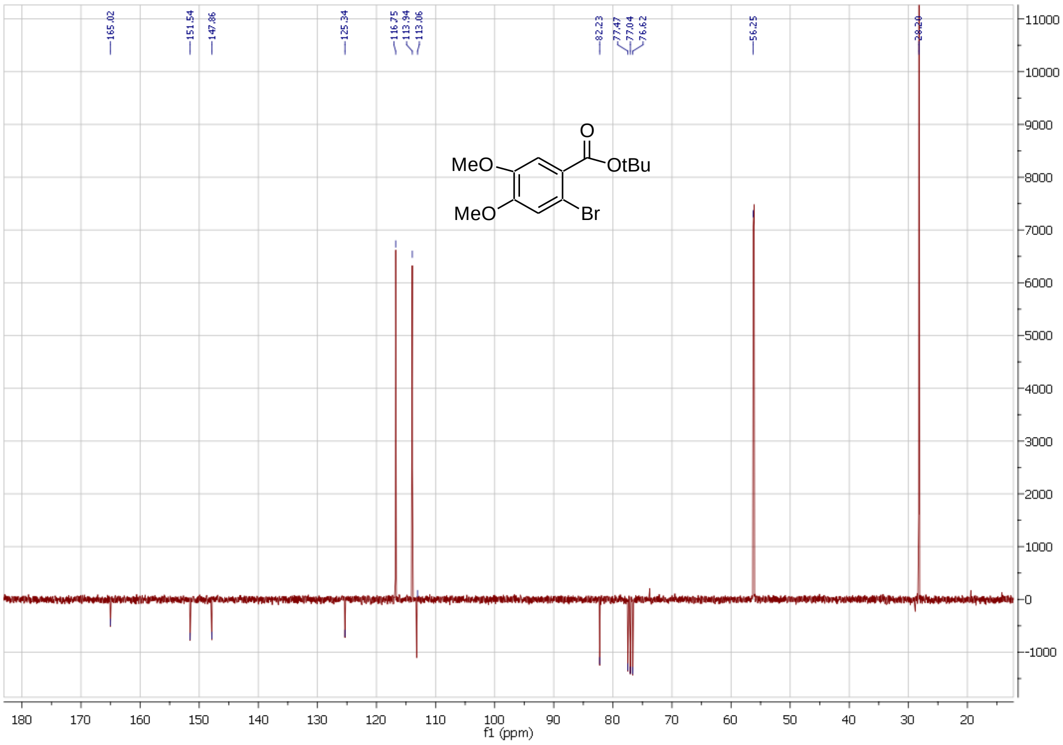

4,5-Dimethoxy-2-((E)-3-oxo-3-(pyrrolidin-1-y)lpropenyl)benzoic acid tert-butyl ester (**13**)

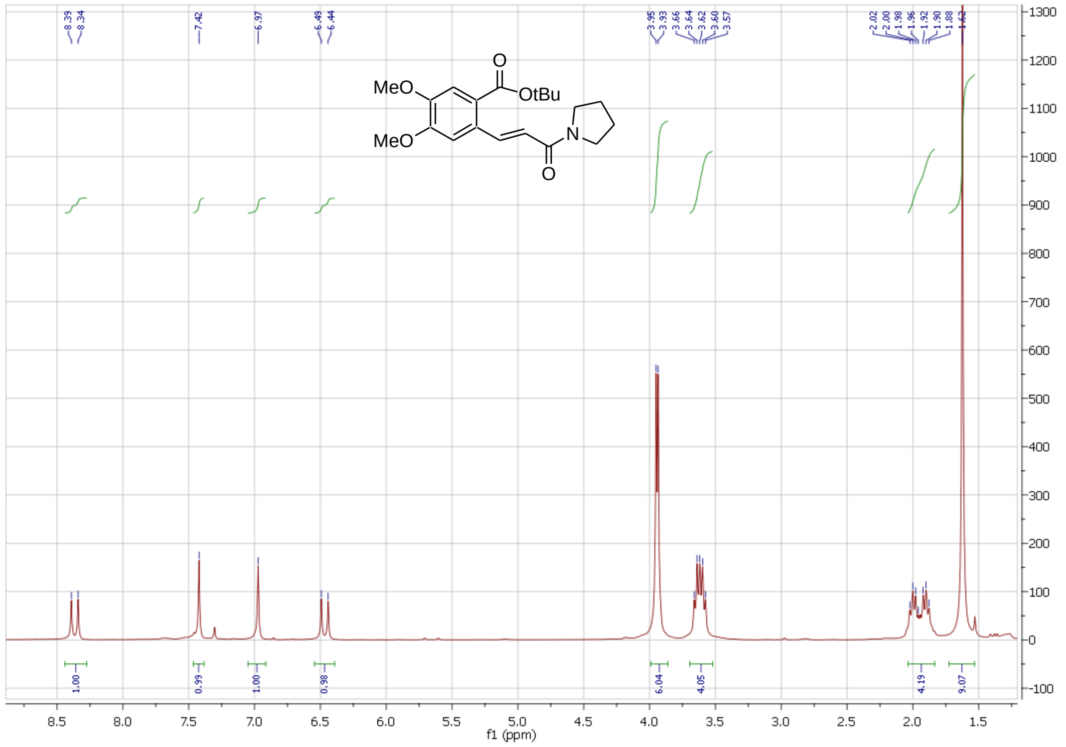

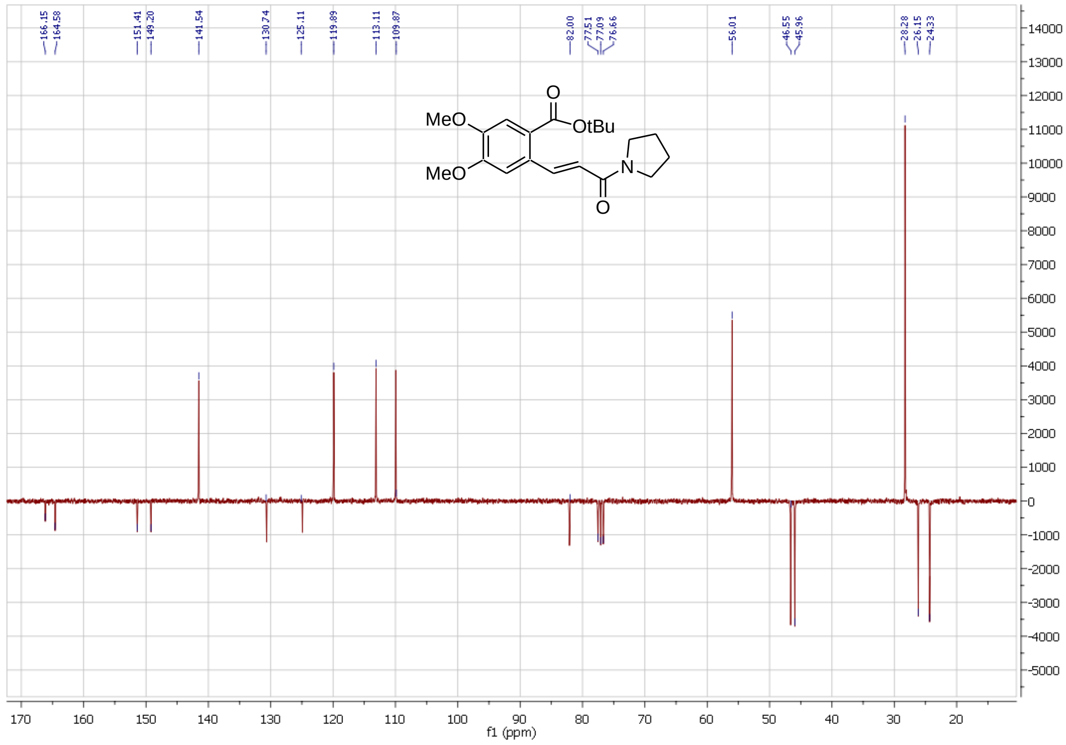

2-((E)-3-Oxo-3-(pyrrolidin-1-yl)propenyl)-N-((R)-1-phenylethyl)benzamide ((*R*)-**6a**)

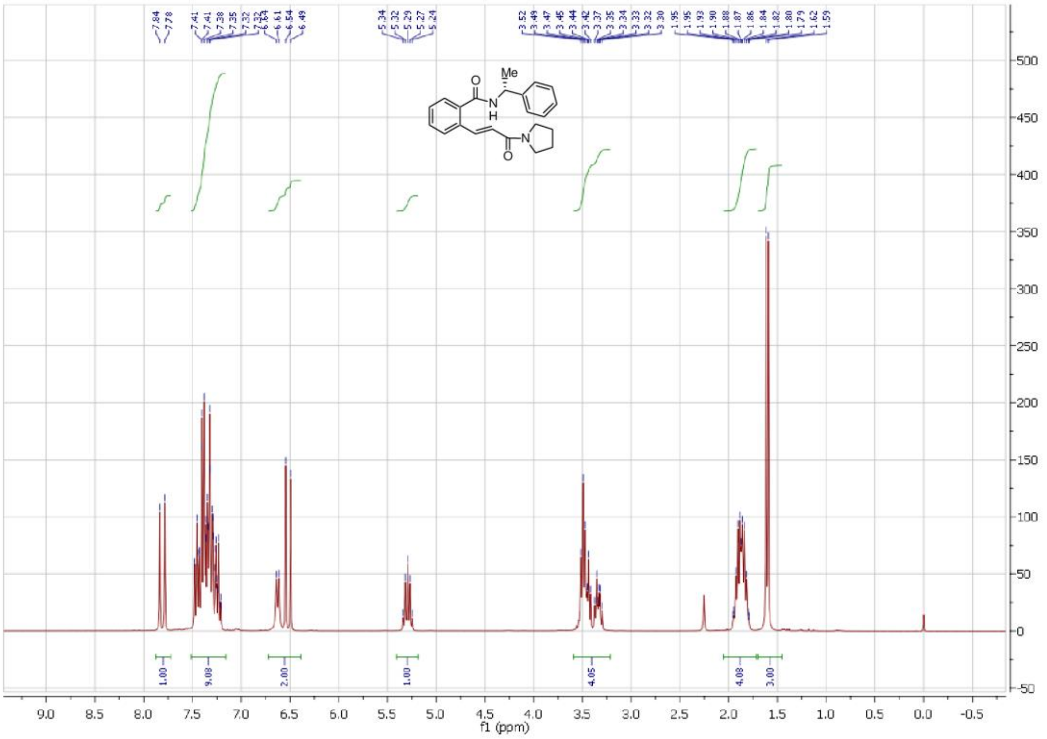

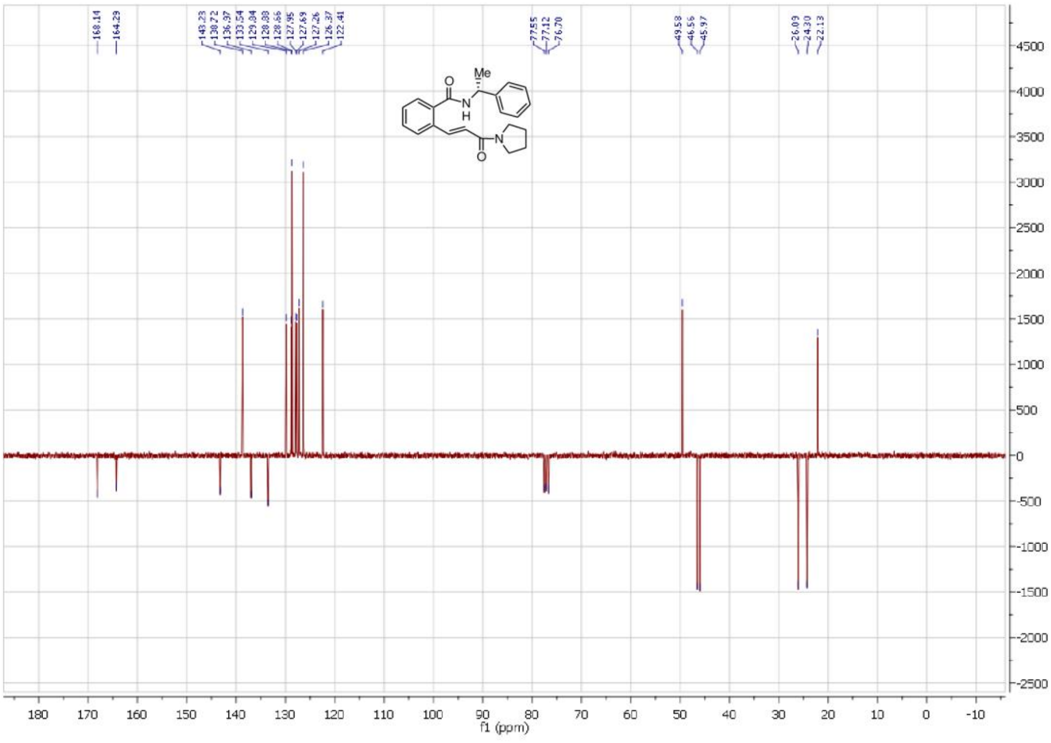

2-((E)-3-(Morpholin-4-yl)-3-oxopropenyl)-N-((R)-1-phenylethyl)benzamide ((*R*)-**6b**)

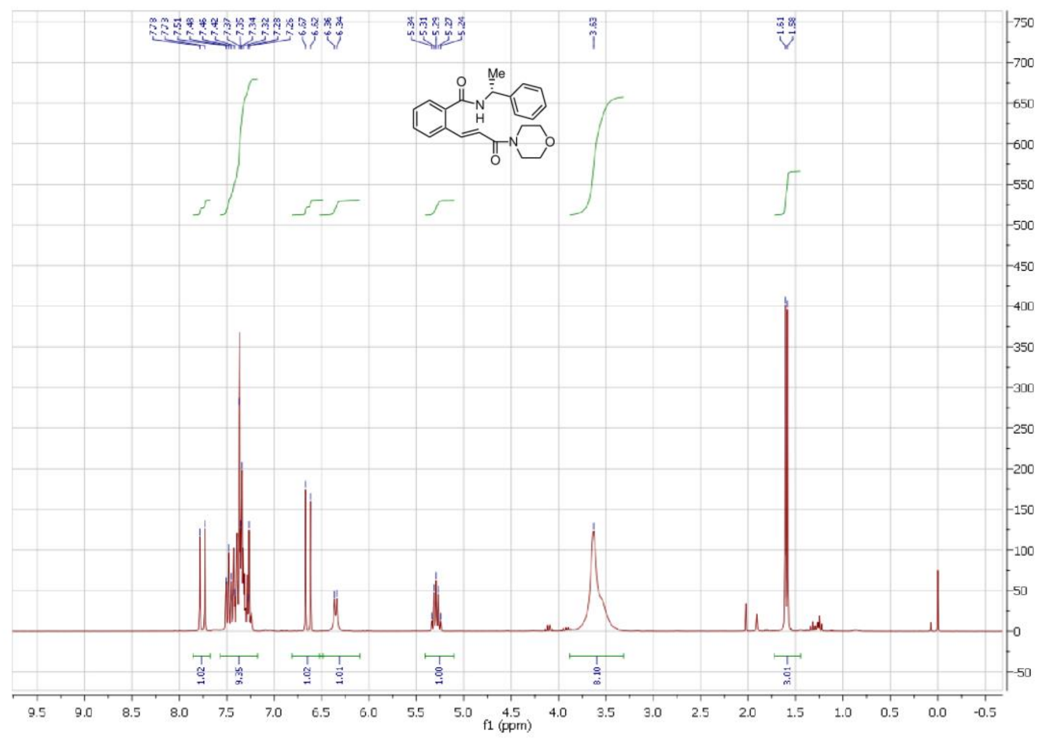

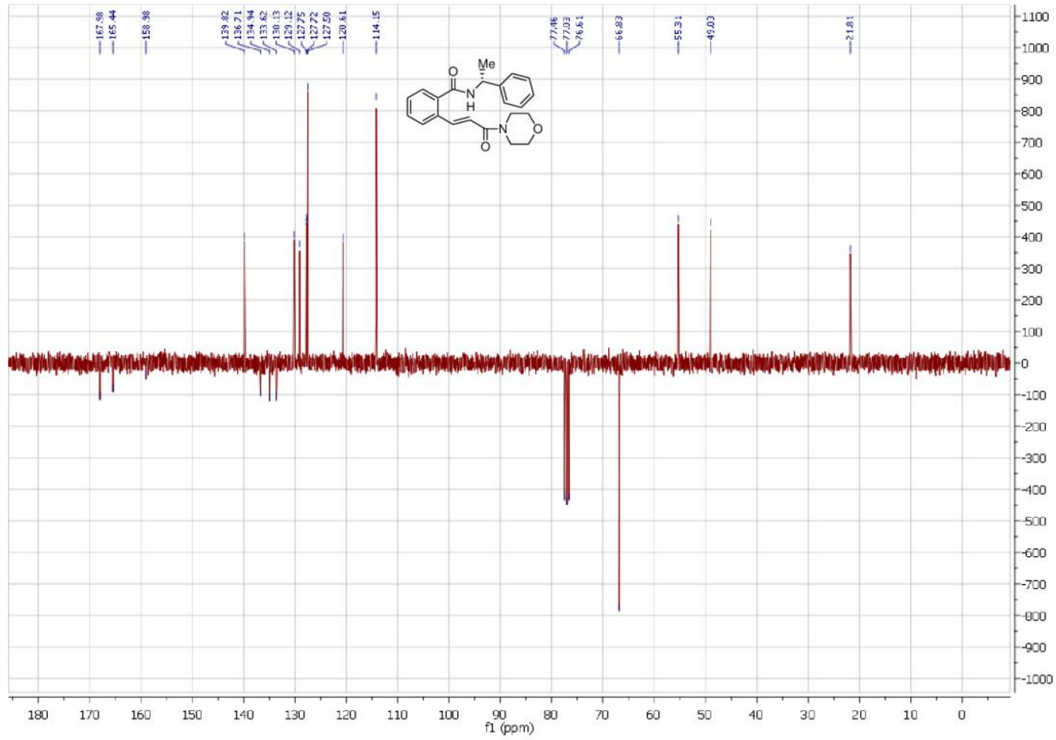

2-((E)-2-(Diisopropylcarbamoyl)vinyl)-N-((R)-1-phenylethyl)benzamide ((*R*)-**6c**)

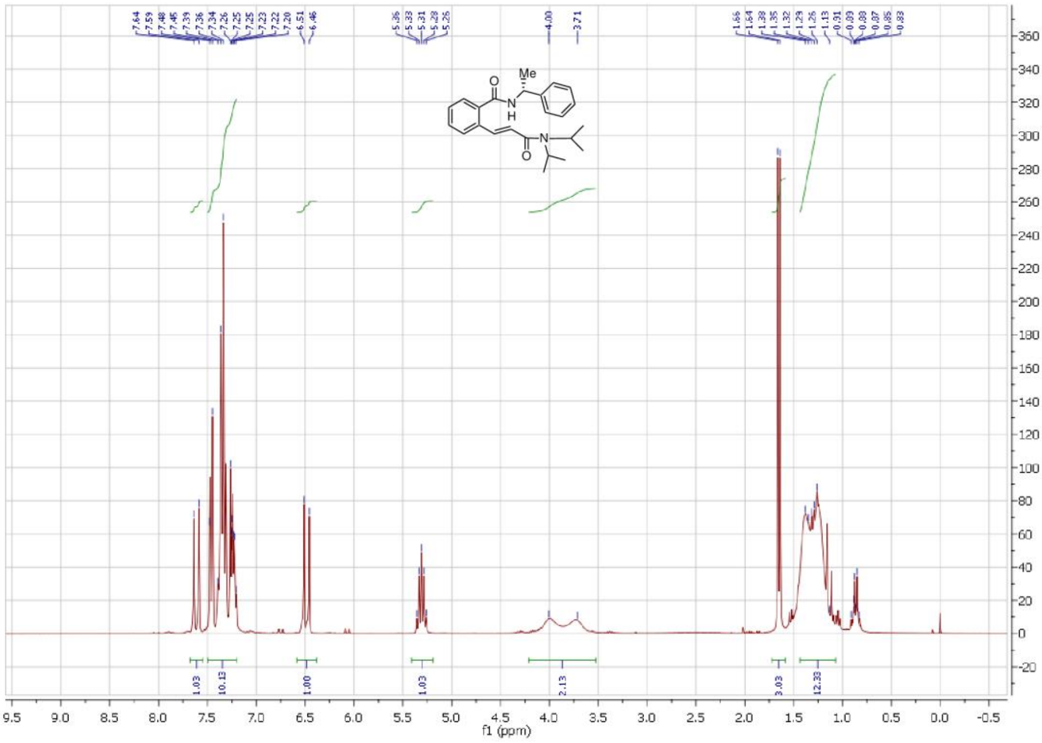

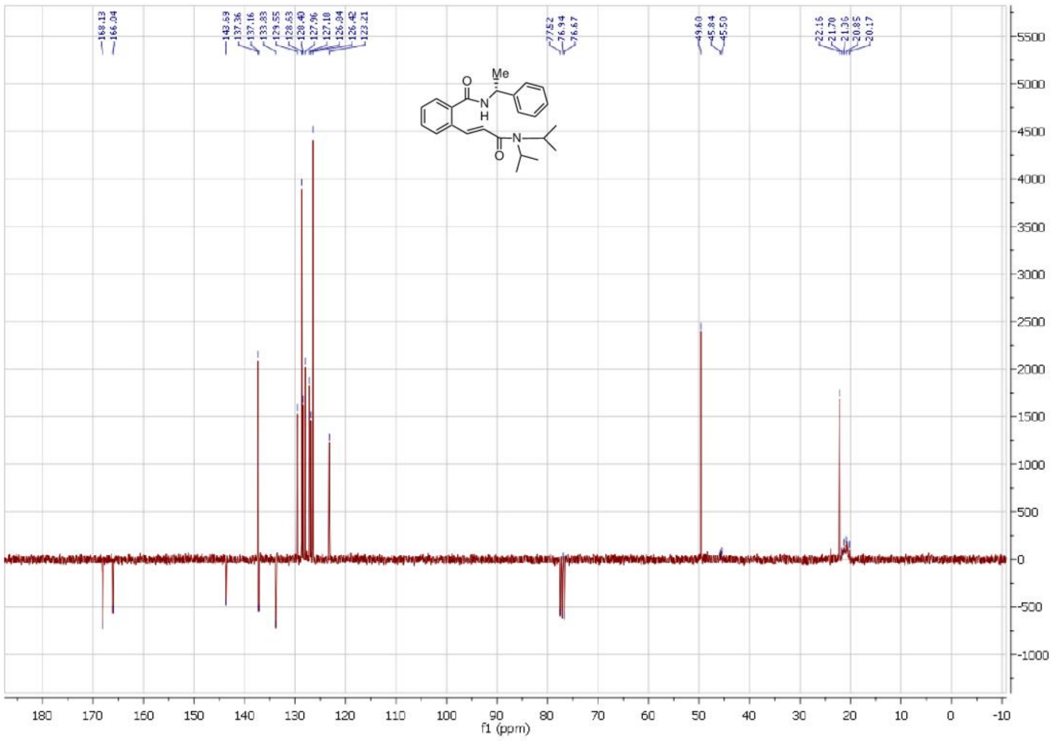

2-((E)-2-(Benzylcarbamoyl)vinyl)-N-((R)-1-phenylethyl)benzamide ((*R*)-**6d**)

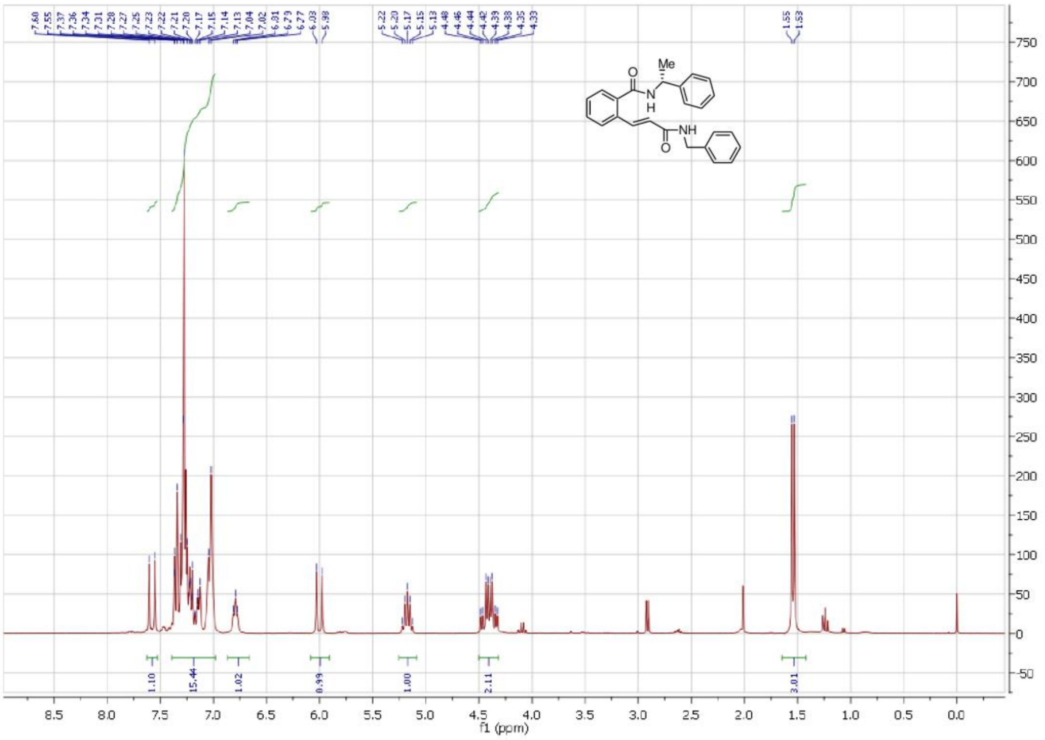

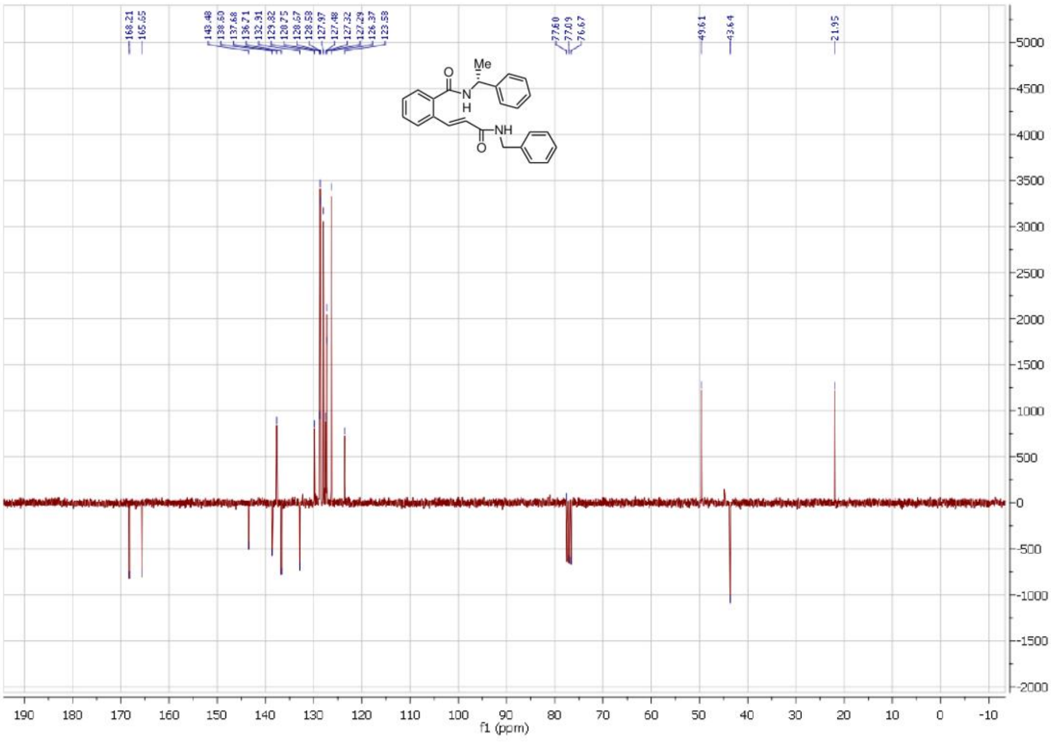

N-((R)-1-(4-Methoxyphenyl)ethyl)-2-((E)-3-oxo-3(-pyrrolidin-1-yl)propenyl)benzamide ((*R*)-**7a**)

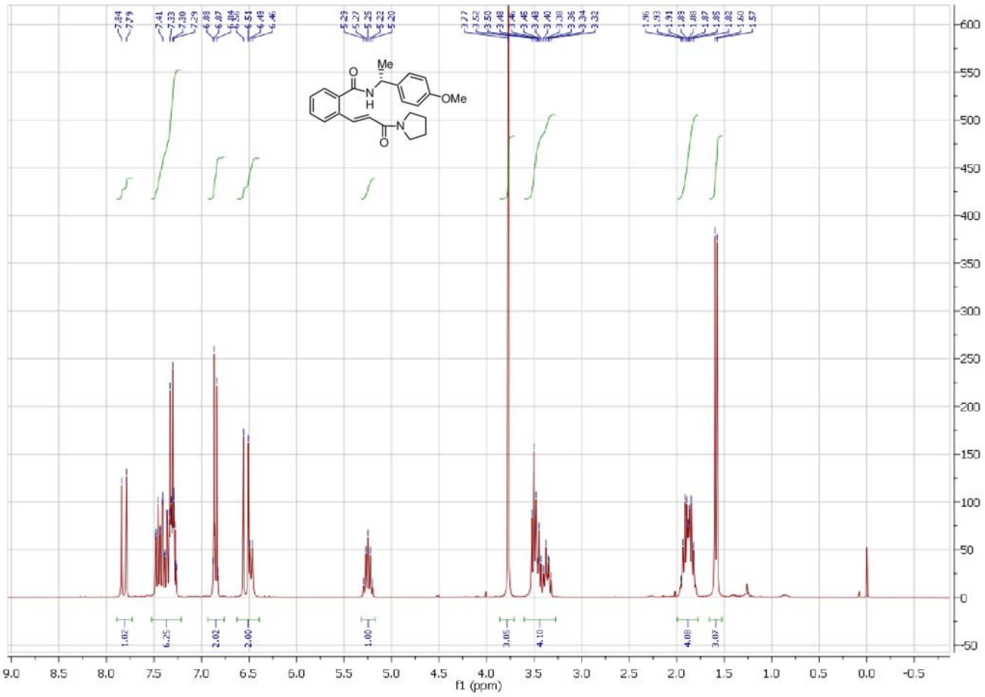

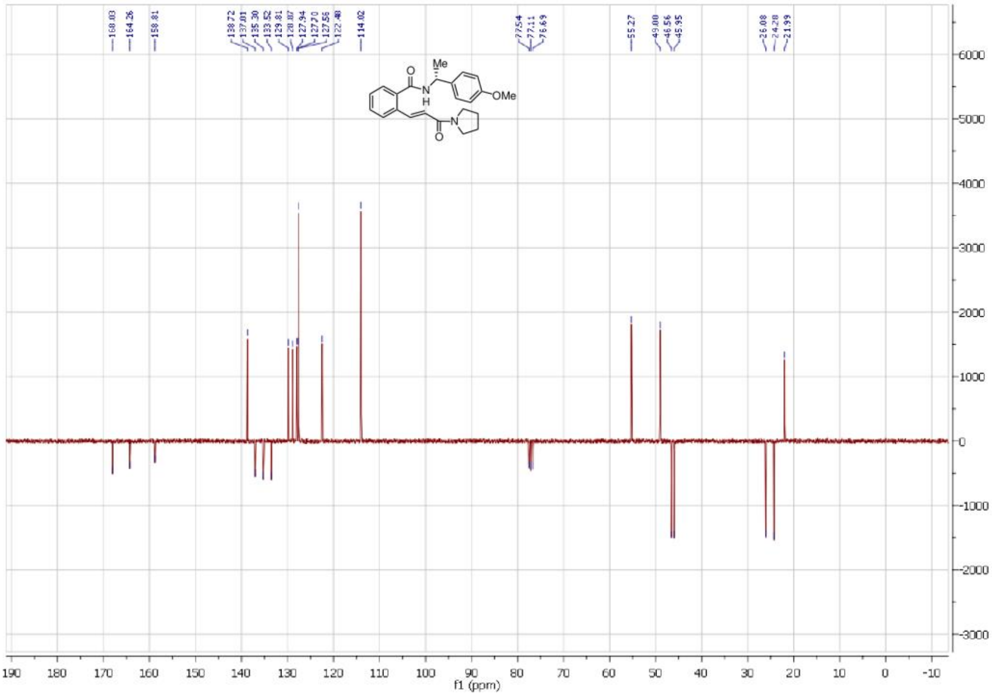

N-((R)-1-(4-Methoxyphenyl)ethyl)-2-((E)-3-morpholin-4-yl-3-oxo-propenyl)-benzamide ((*R*)-**7b**)

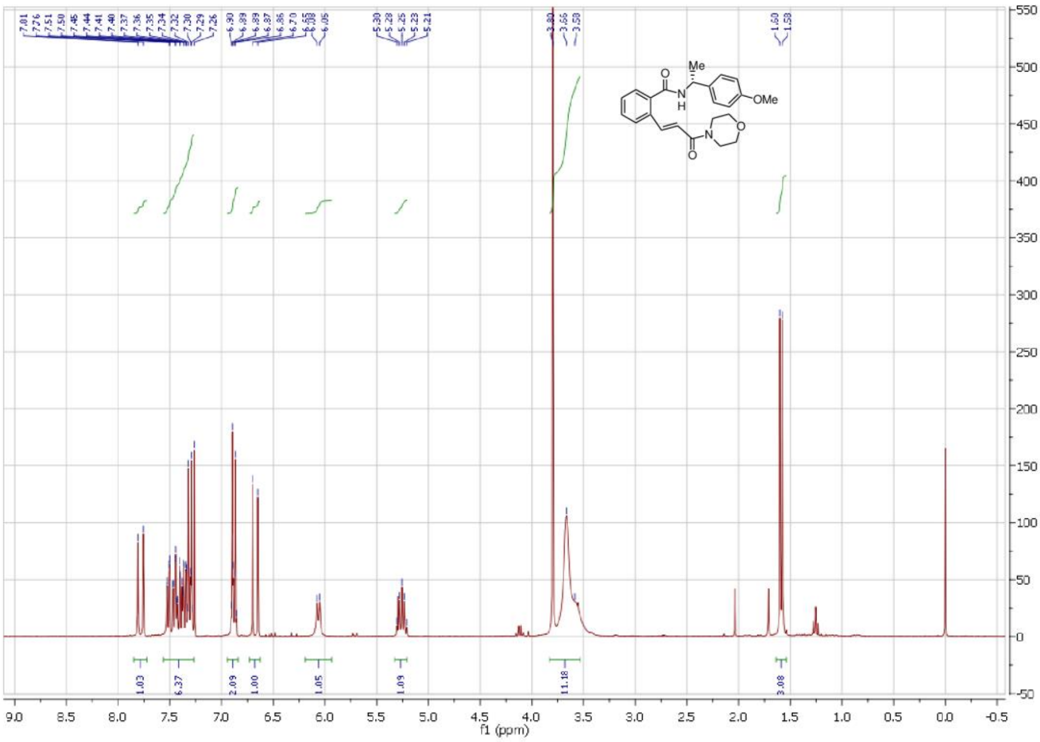

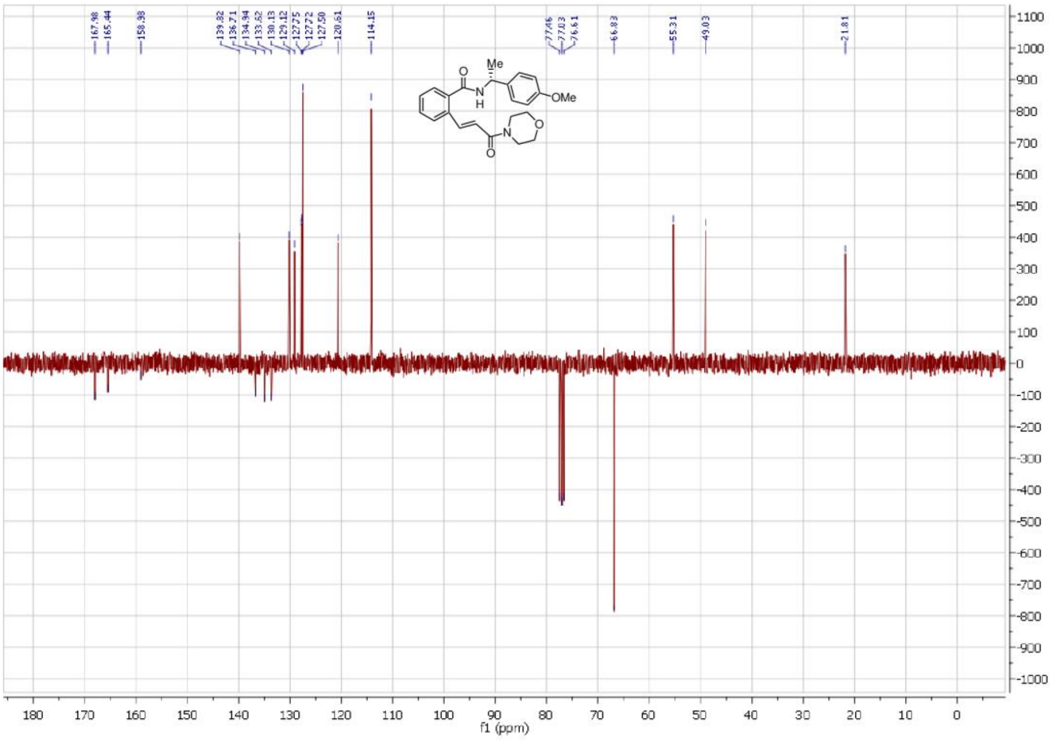

2-((E)-2-(Diisopropylcarbamoyl)vinyl)-N-((R)-1-4-(methoxyphenyl)ethyl)benzamide ((*R*)-**7c**)

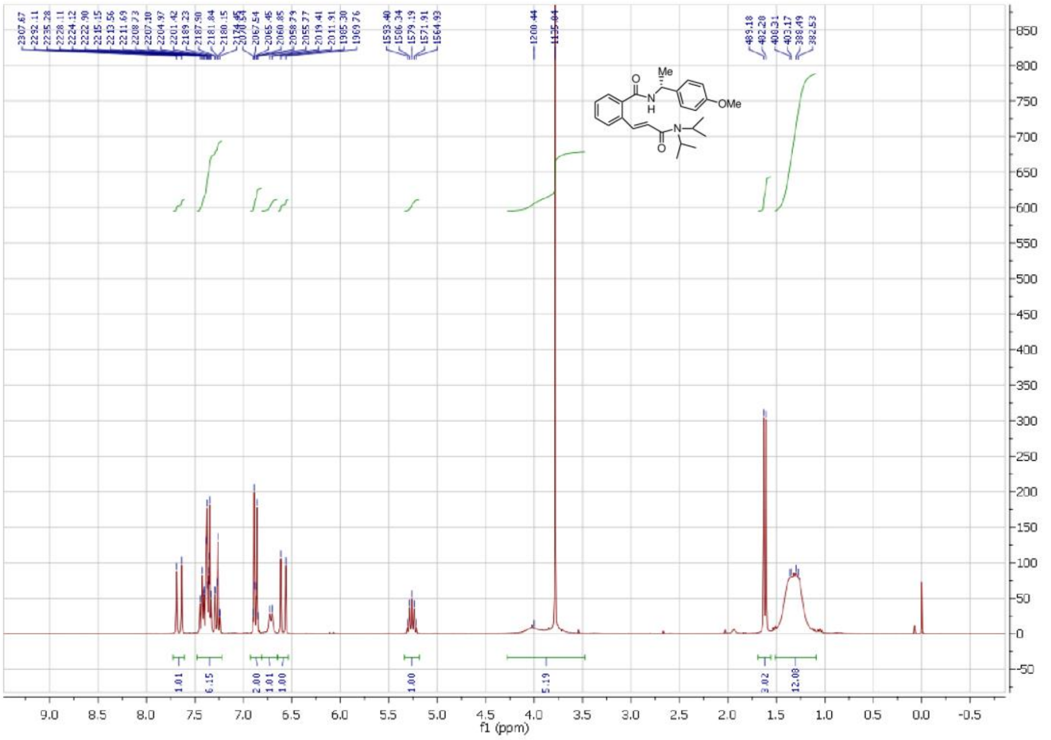

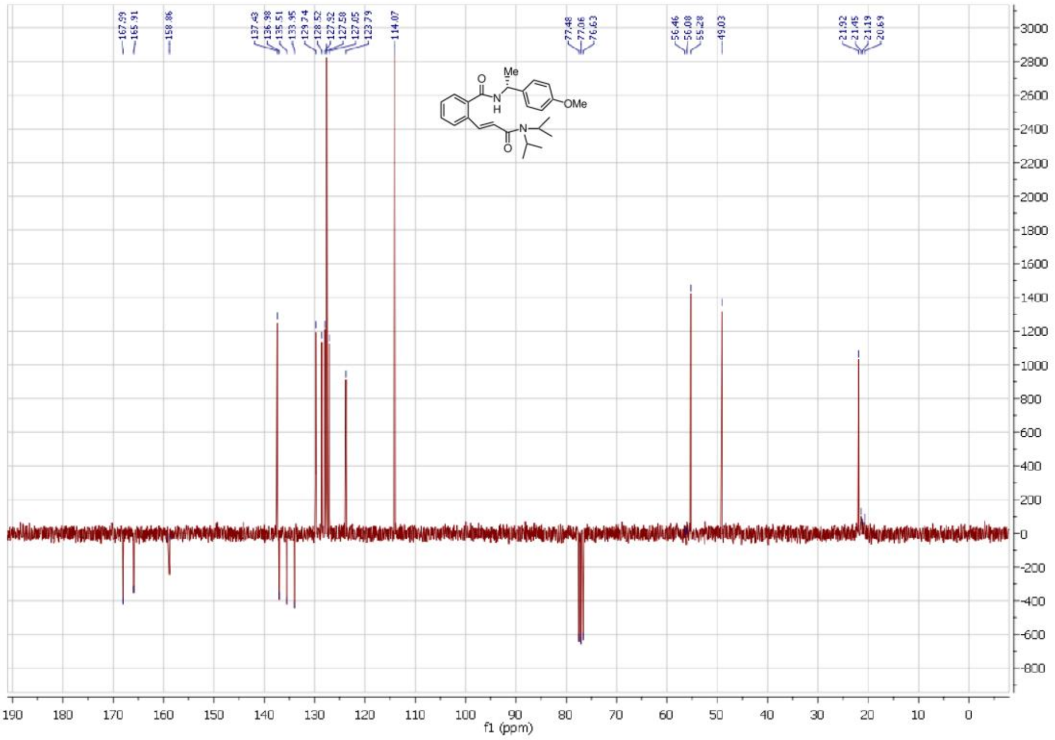

2-((E)-2-(Benzylcarbamoyl)vinyl)-N-((R)-1-(4-methoxyphenyl)ethyl)benzamide ((*R*)-**7d**)

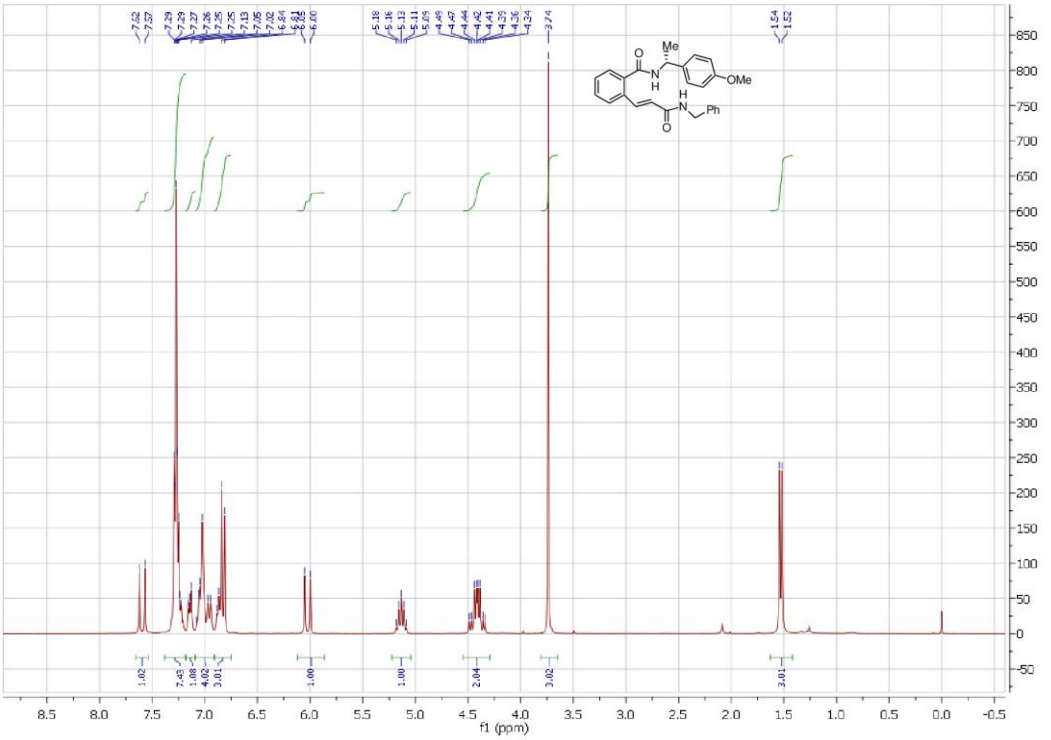

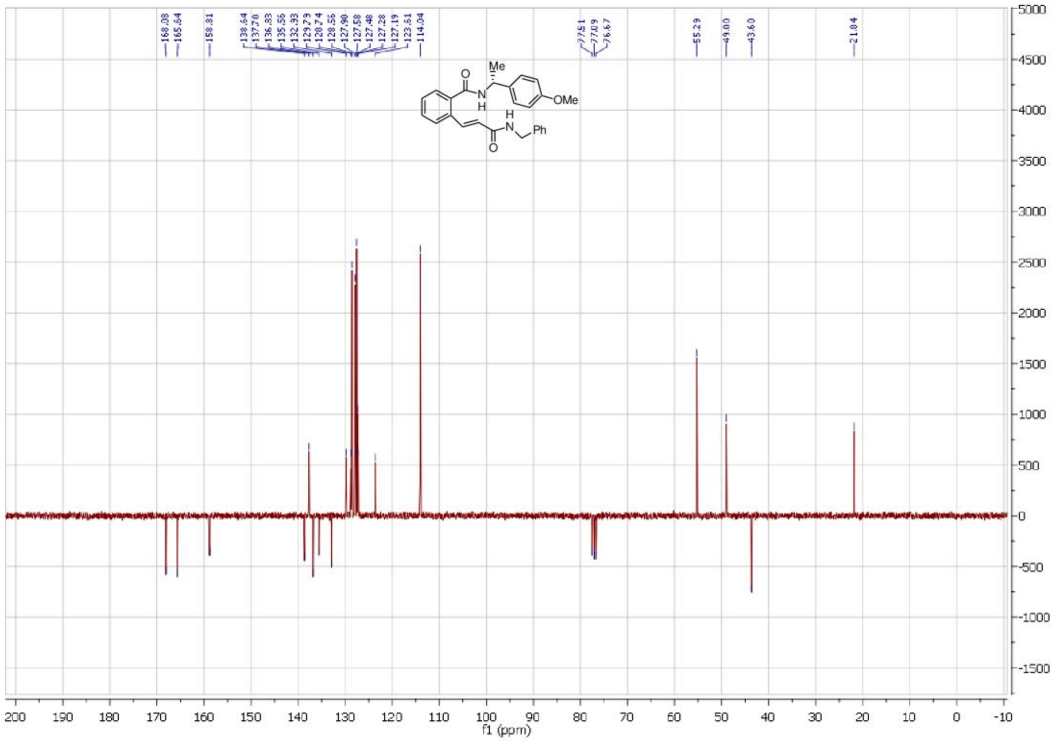

2-((E)-3-(Dicyclohexylamino)-3-oxo-propen-1-yl)-N-((R)-1-(4-methoxyphenyl)ethyl)-benzamide ((*R*)-**7e**)

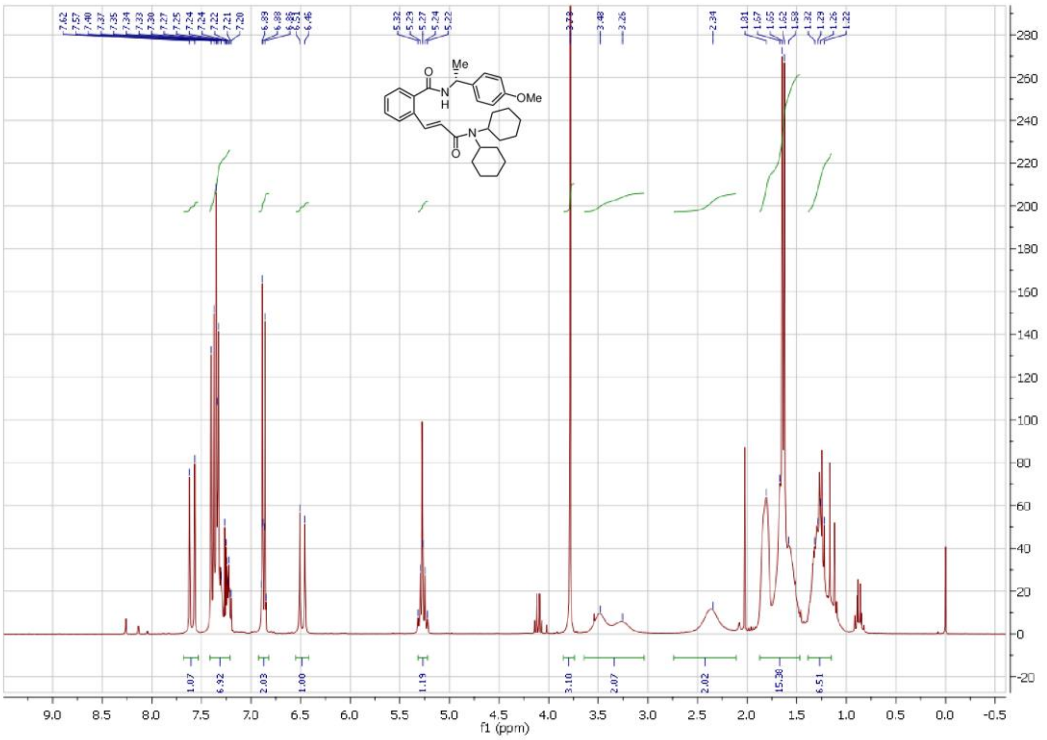

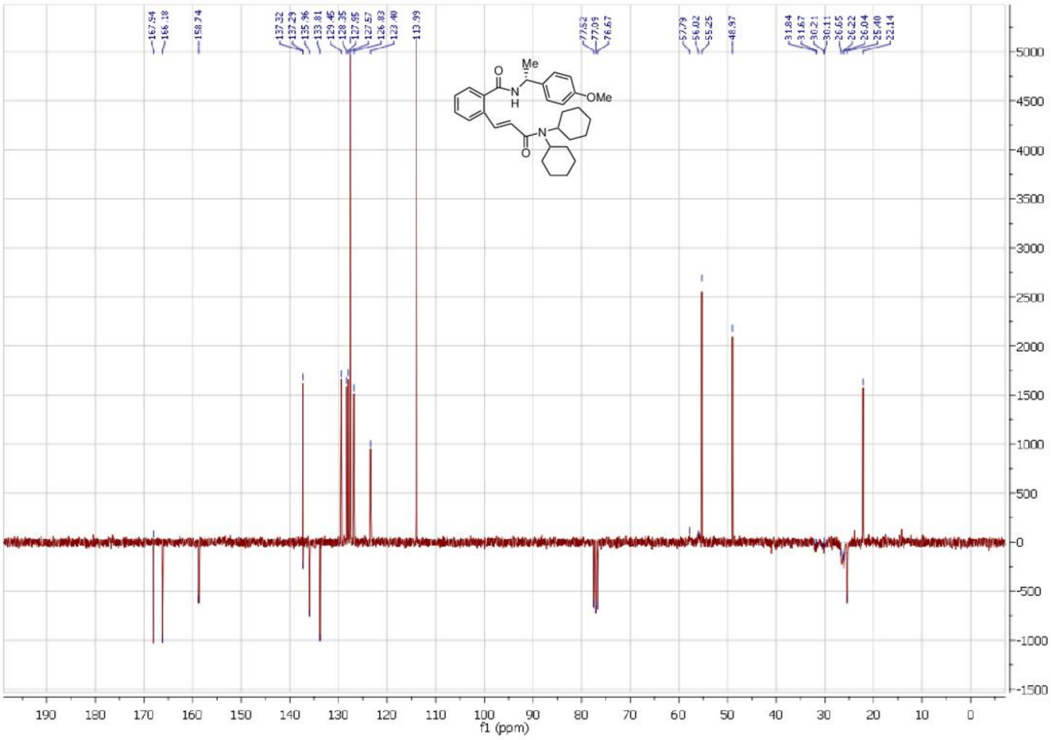

(E)-3,4-Dimethoxy-N-((R)-1-(4-methoxyphenyl)ethyl)-2-((E)-3-oxo-(3-pyrrolidin-1-yl)propen-1-yl)benzamide ((R)-**8**)

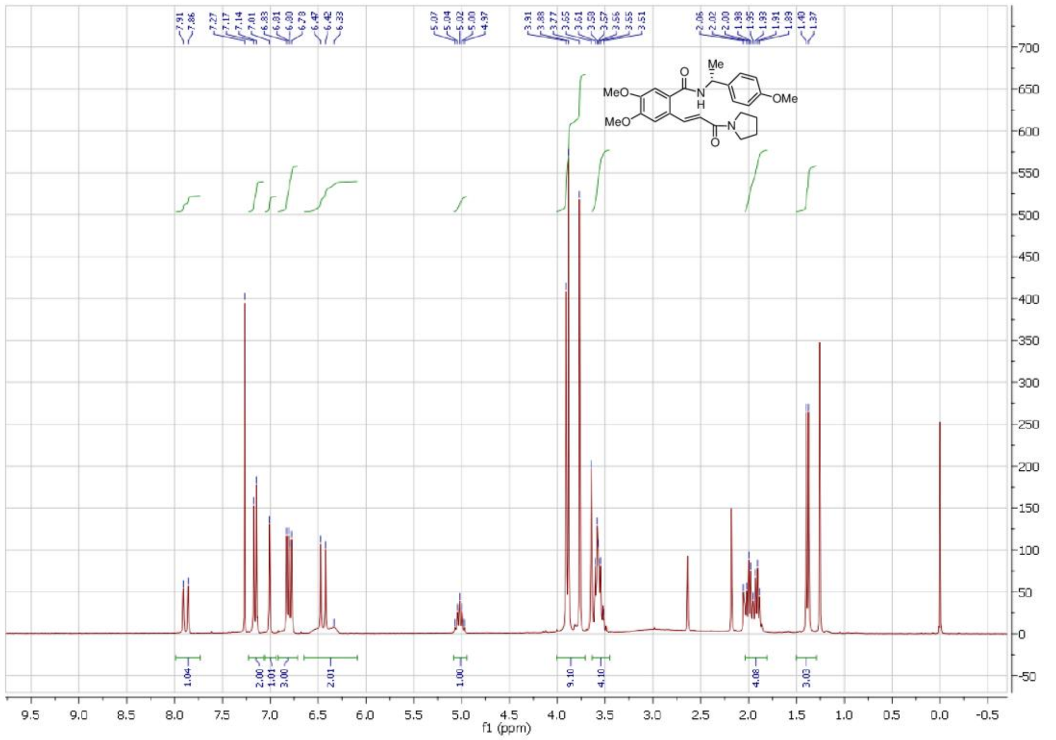

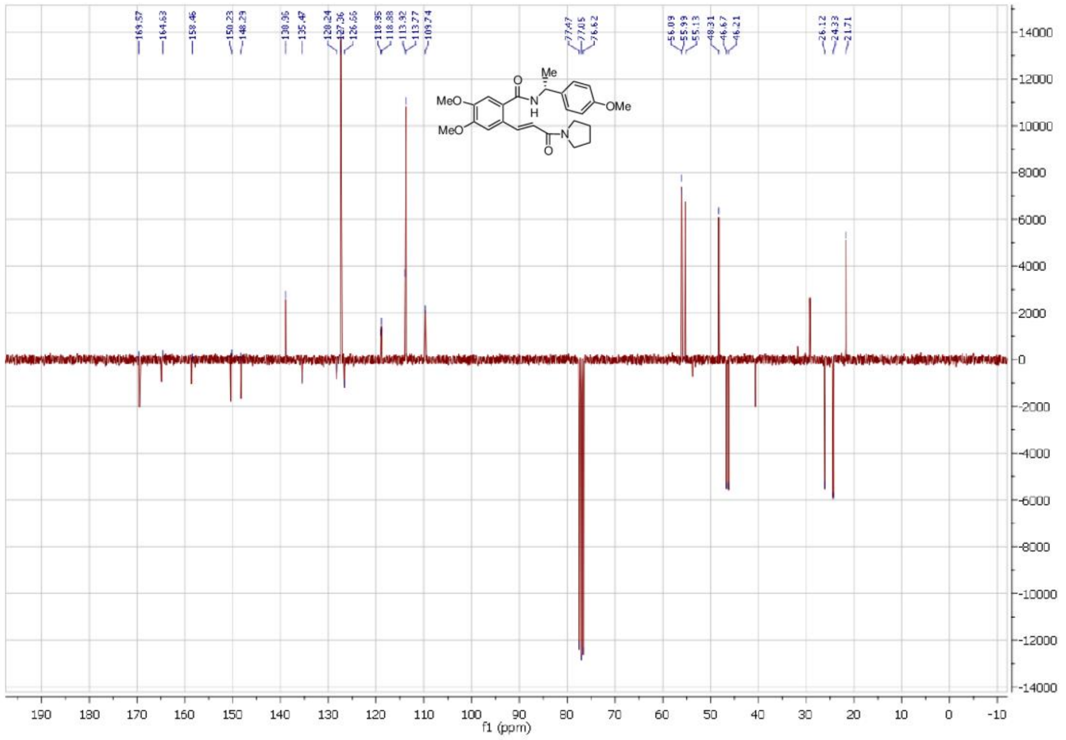

(2S,3S)-3-(2-Oxo-2-(pyrrolidin-1-yl)ethyl)-2-(1-phenylethyl)-2,3-dihydro-isoindol-1-one ((*S*)-**3a**)

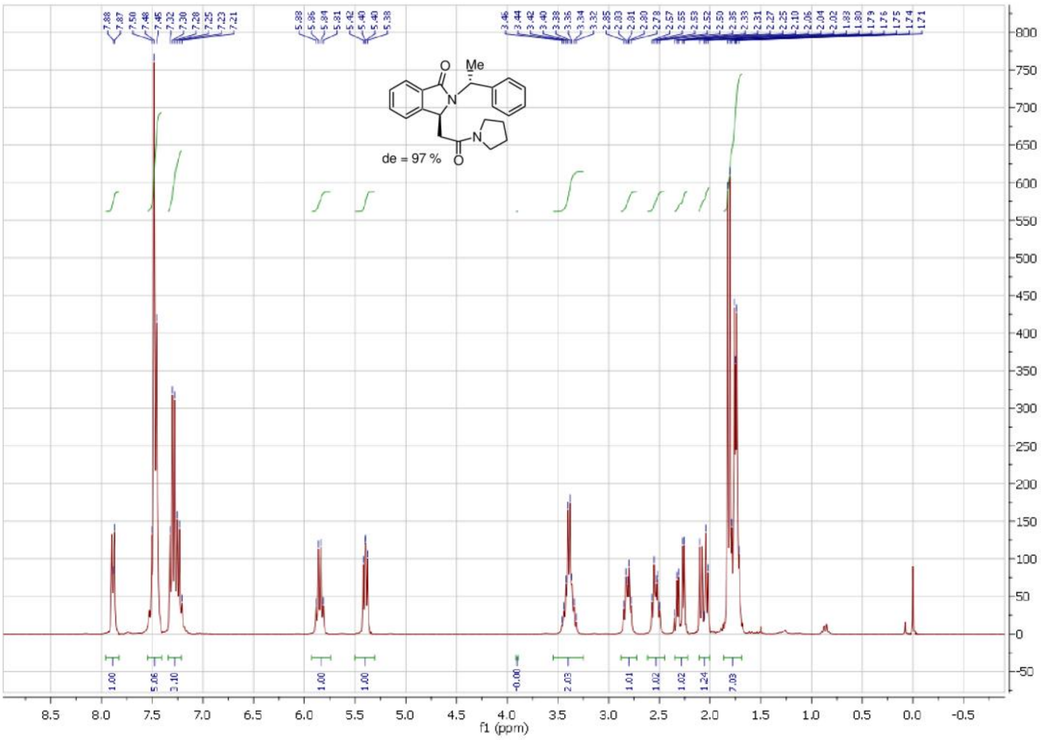

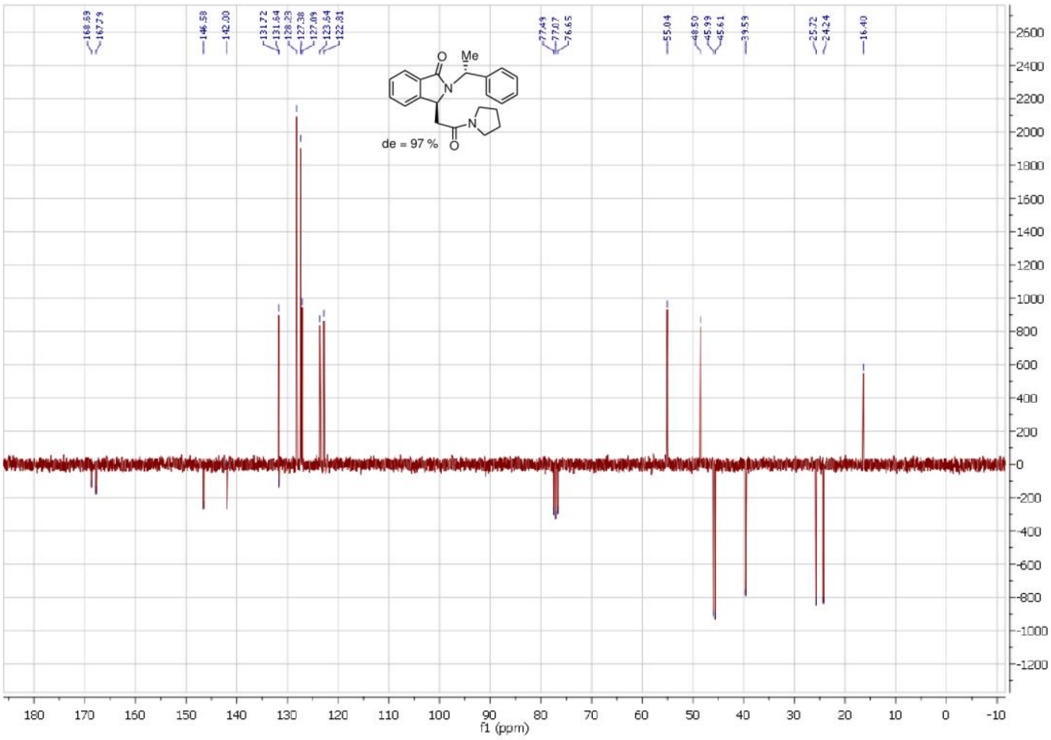

(2R,3S)-3-(2-(Morpholin-4-yl)-2-oxoethyl)-2-(1-phenylethyl)-2,3-dihydro-isoindol-1-one ((*R*)-**3b**)

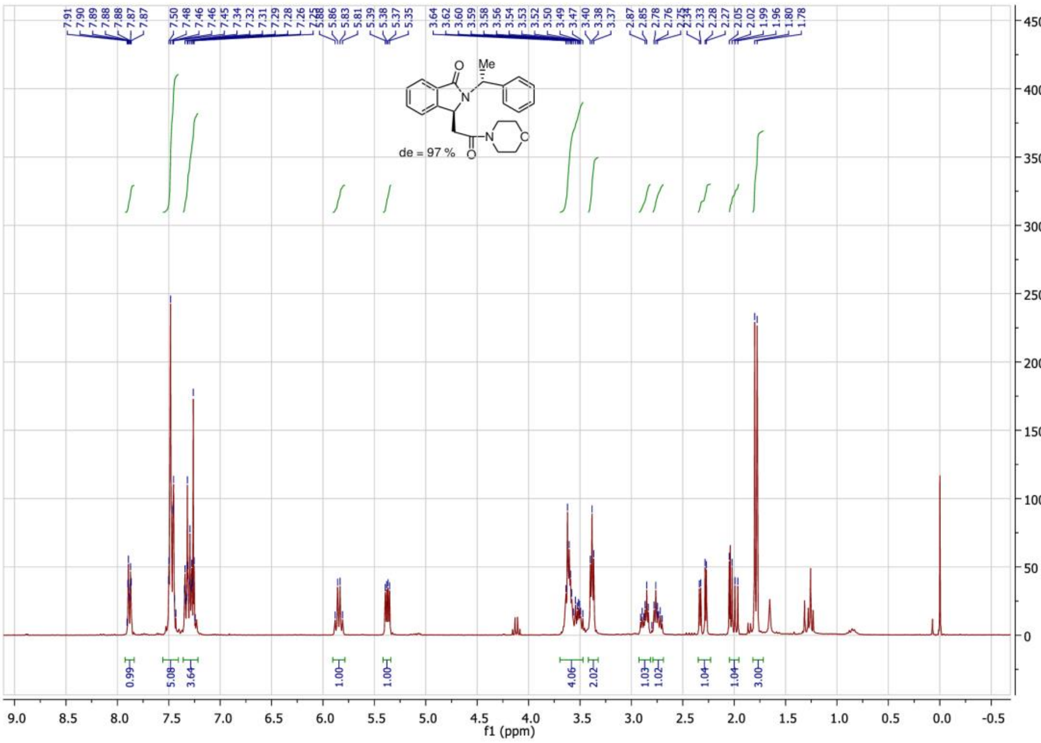

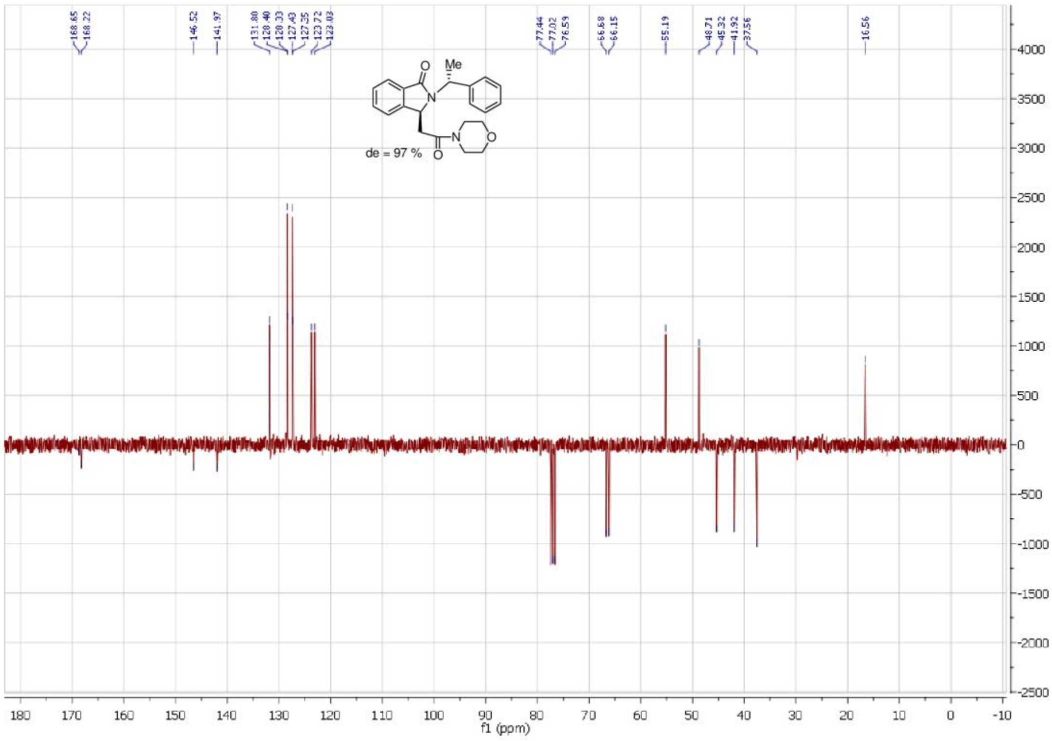

(2R,3S)-N,N-Diisopropyl-2-[3-oxo-2-(1-phenylethyl)-2,3-dihydro-1H-isoindol-1-yl]acetamide ((*R*)-**3c**)

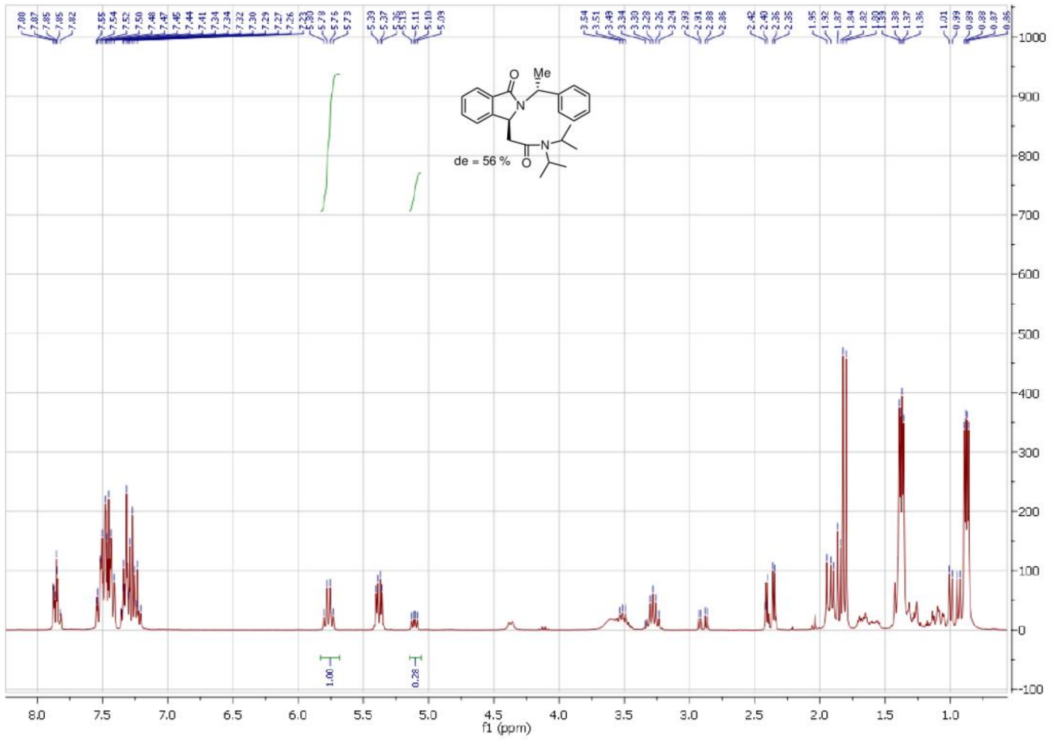

(2R,3S)-N-Benzyl-2-(3-Oxo-2-(1-phenyl-ethyl)-2,3-dihydro-1H-isoindol-1-yl]-acetamide ((*R*)-**3d**)

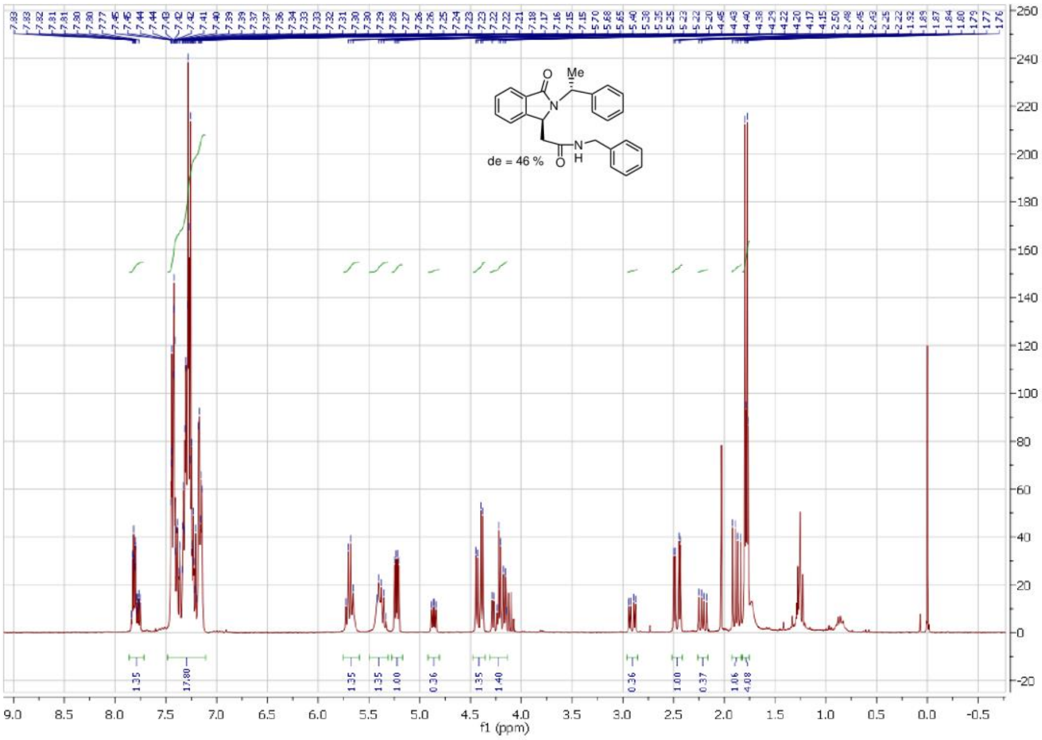

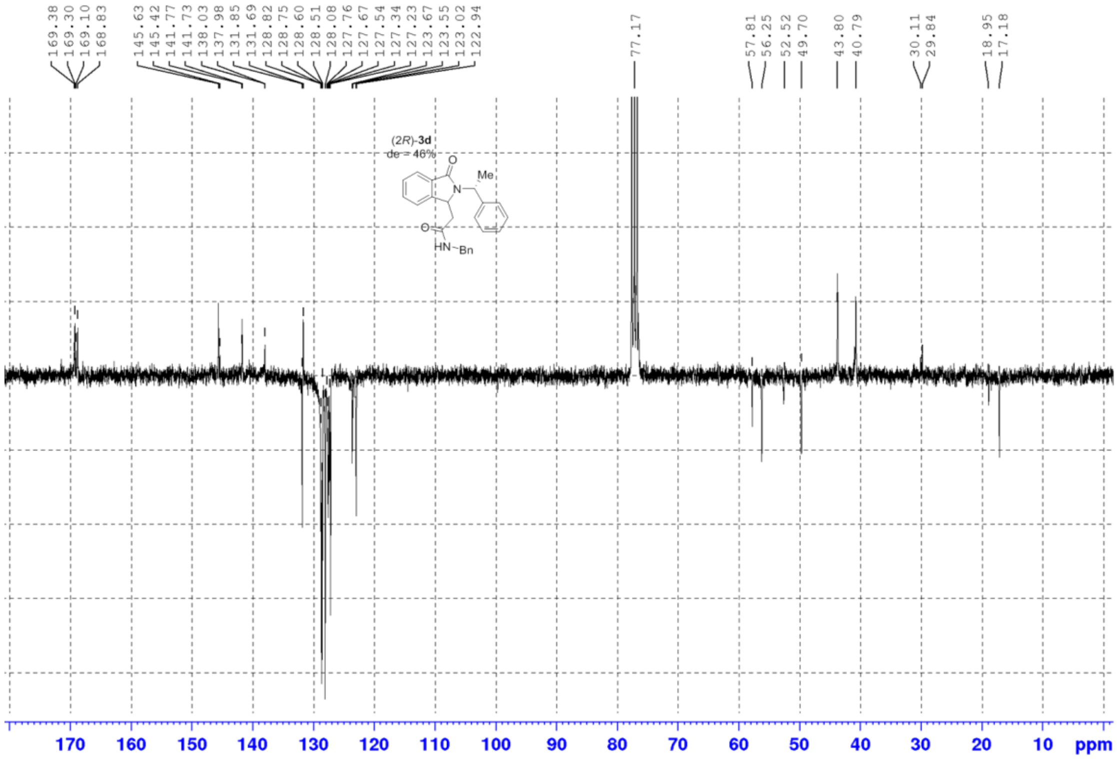

(2R,3S)-2-(1-(4-Methoxyphenyl)ethyl)-3-(2-oxo-2-(pyrrolidin-1-yl)ethyl))-2,3-dihydroisoindol-1-one ((*R*)-**4a**)

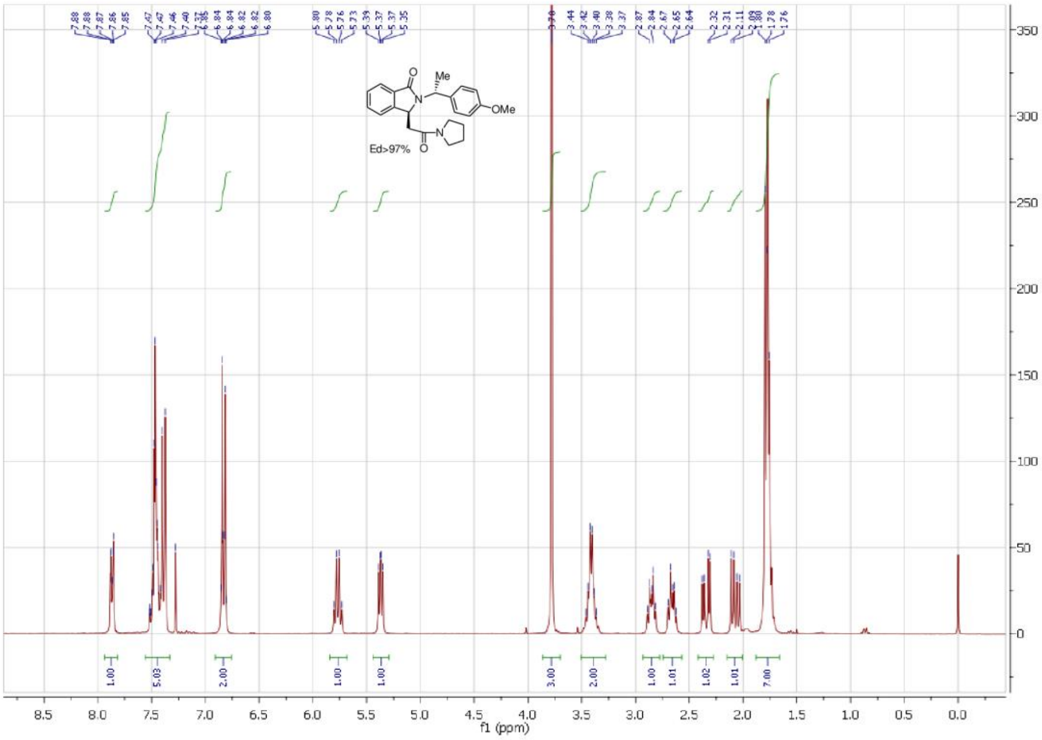

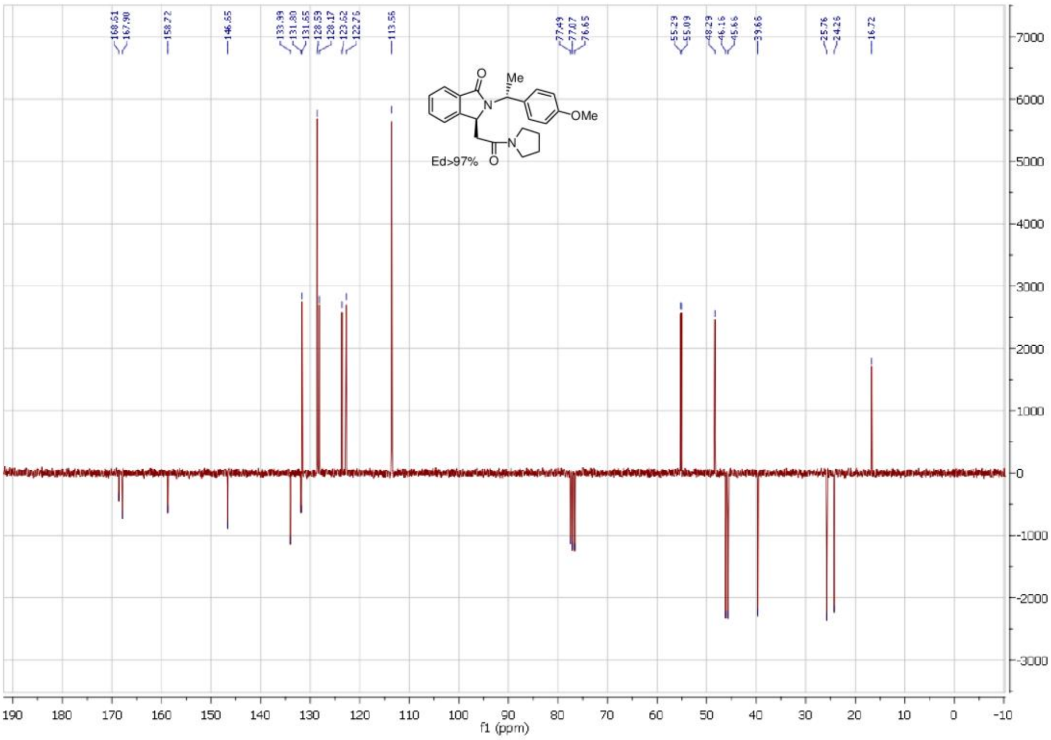

(2R,3S)-2-(1-(4-Methoxyphenyl)ethyl)-3-(2-(morpholin-4-y)l-2-oxo-ethyl))-2,3-dihydroisoindol-1-one ((*R*)-**4b**)

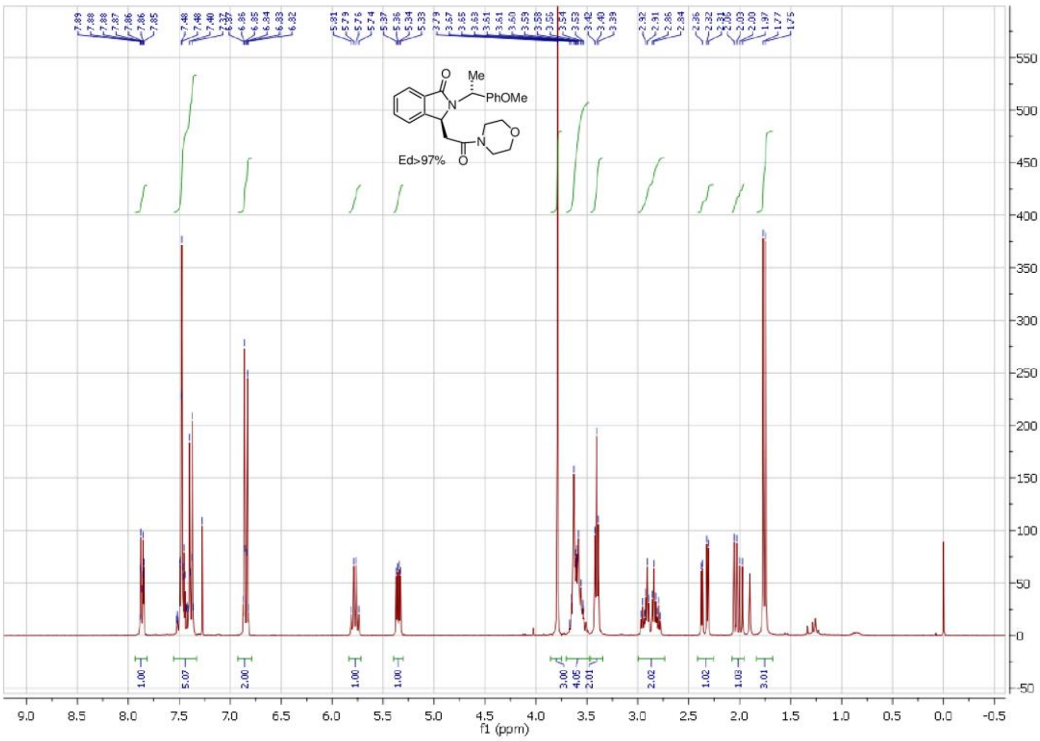

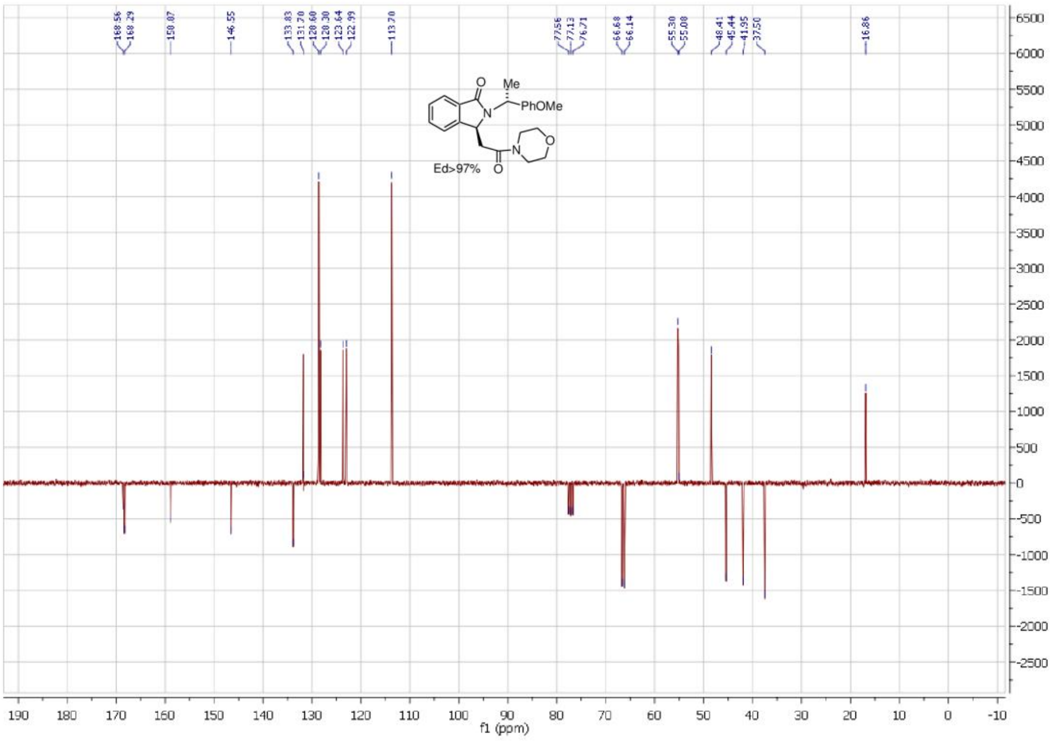

(2R,3S)-N,N-Diisopropyl-2-[2-(1-(4-methoxyphenyl)ethyl)-3-oxo-2,3-dihydro-1H-isoindol-1-yl]acetamide ((*R*)-**4c**)

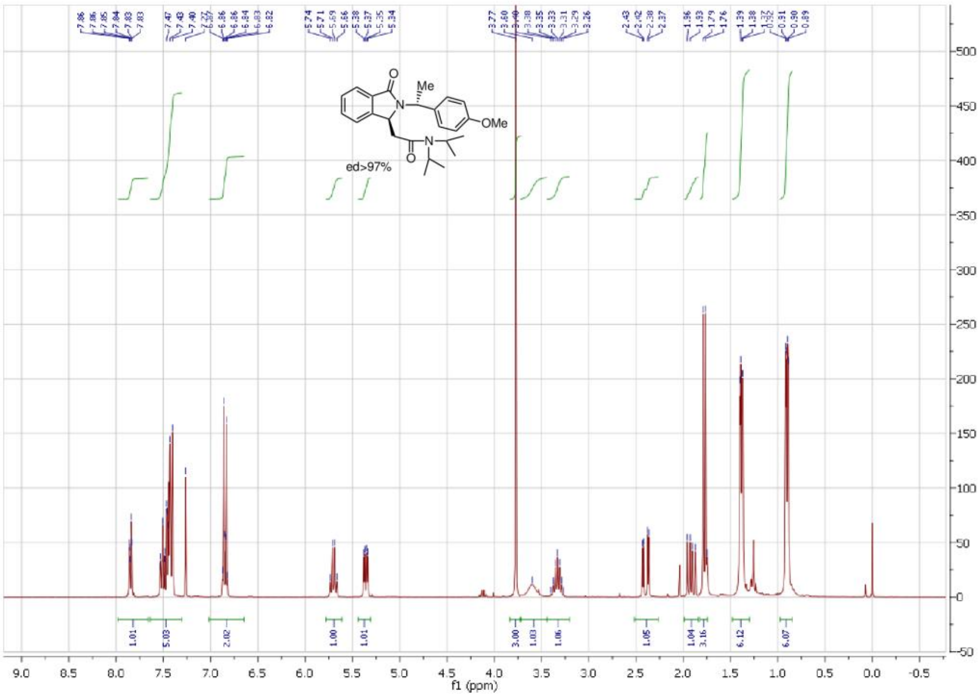

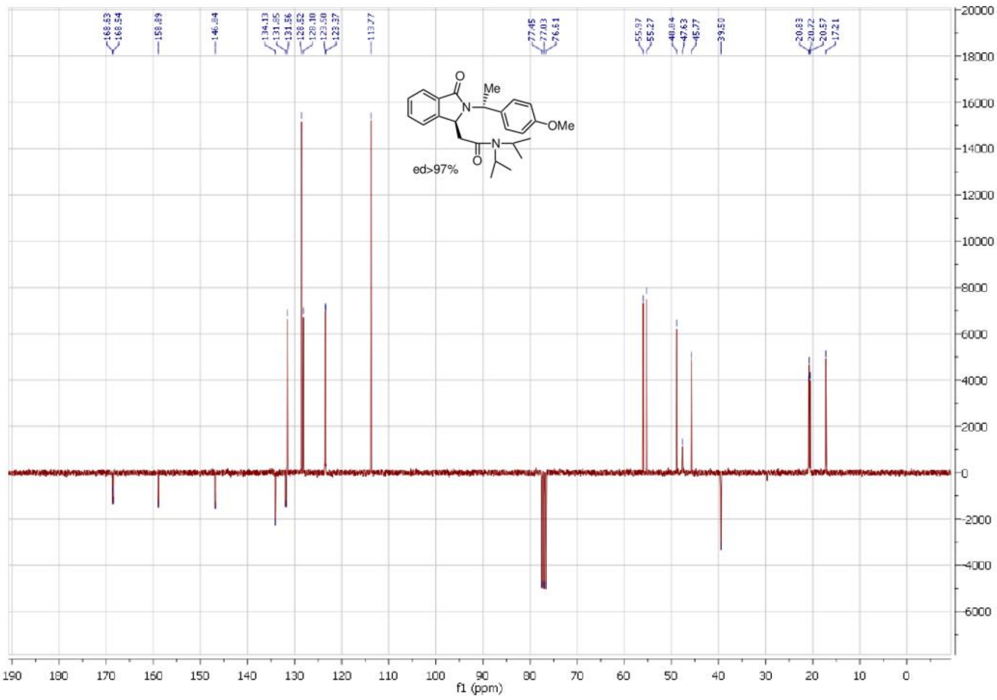

(2R,3S)-N-Benzyl-2-(2-(1-(4-methoxyphenyl)ethyl)-3-oxoisoindolin-1-yl)acetamide ((*R*)-**4d**)

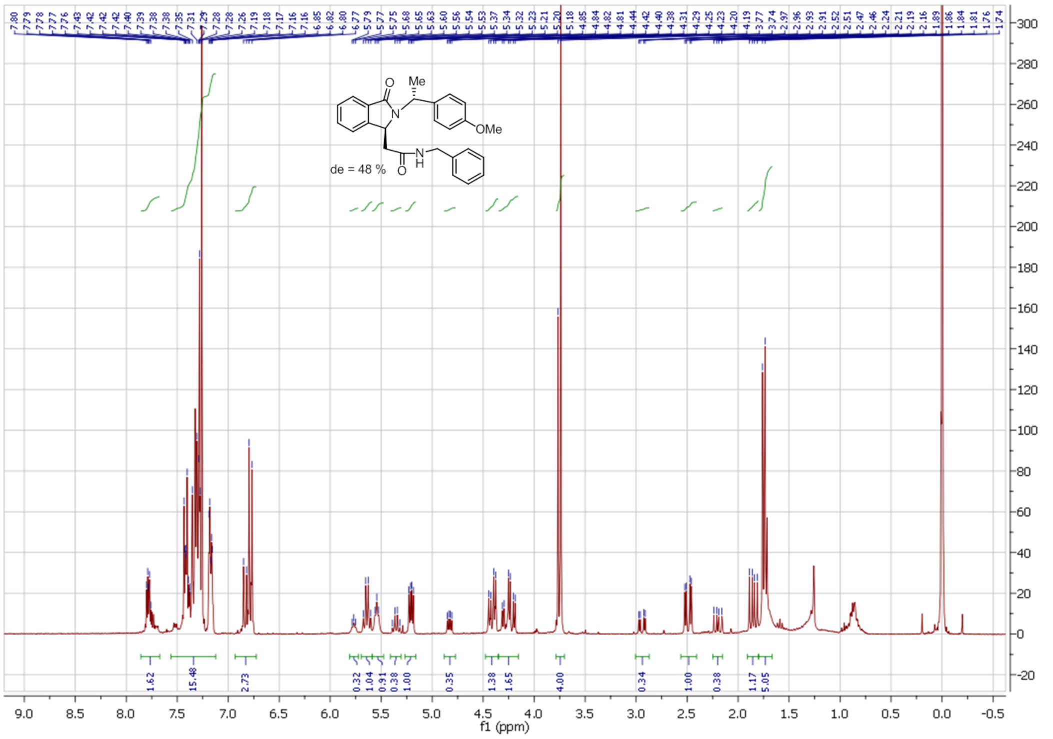

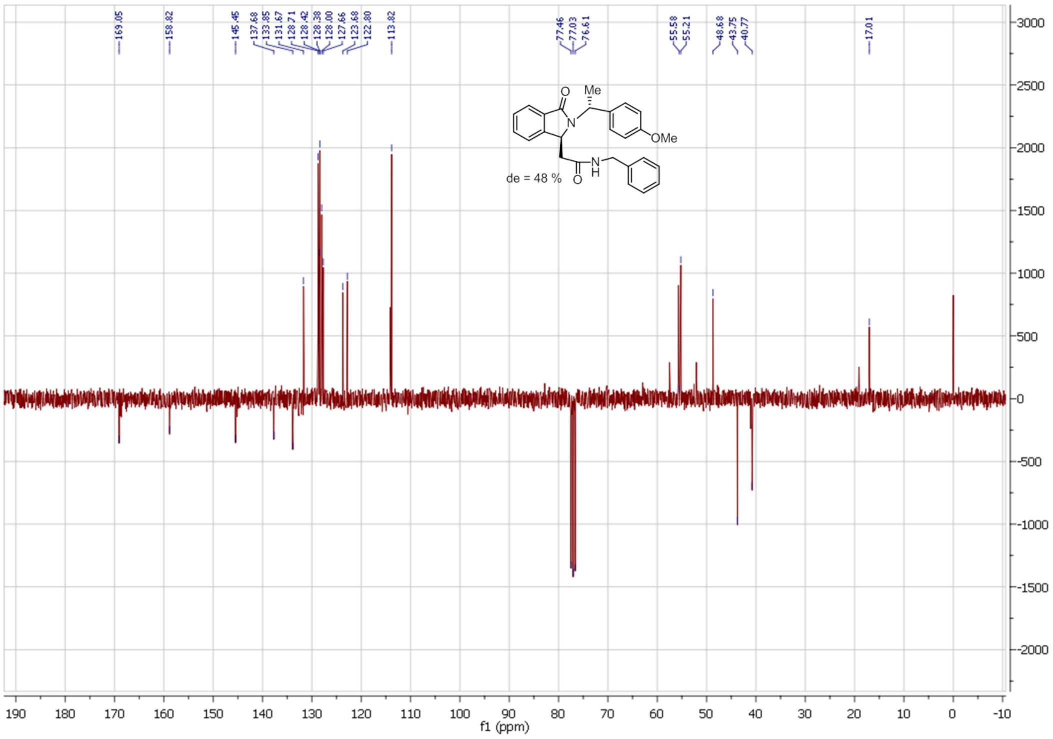

(2R,3S)-N,N-Dicyclohexyl-2-[2-(1-(4-methoxyphenyl)ethyl)-3-oxo-2,3-dihydro-1H-isoindol-1-yl]acetamide ((*R*)-**4e**)

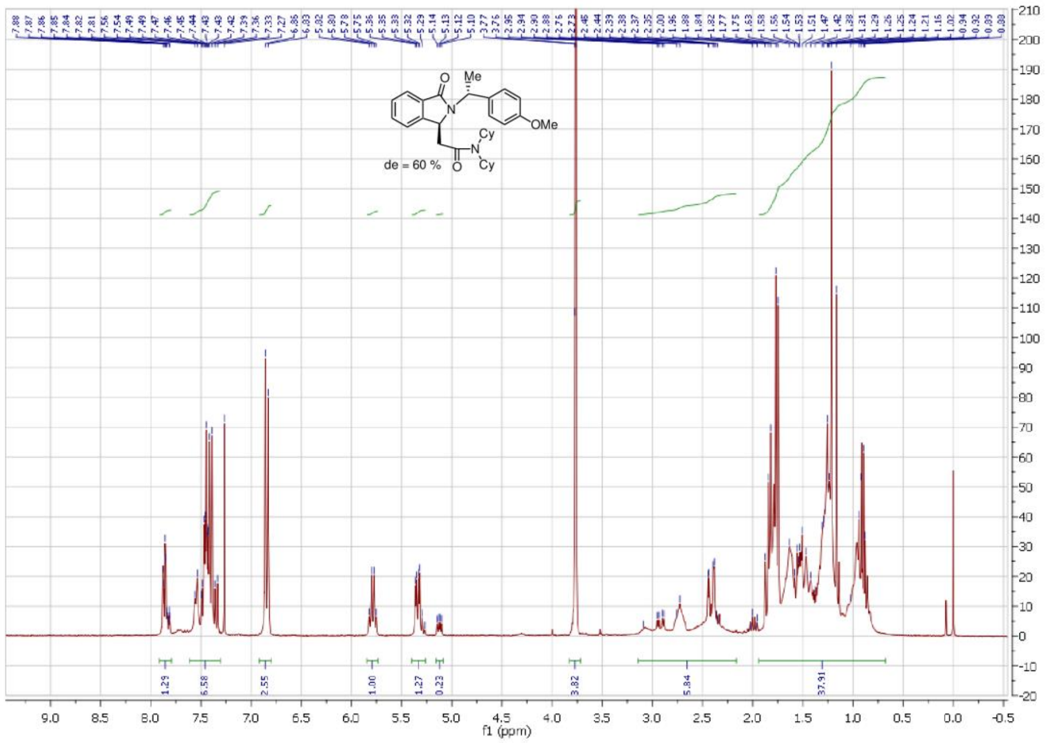

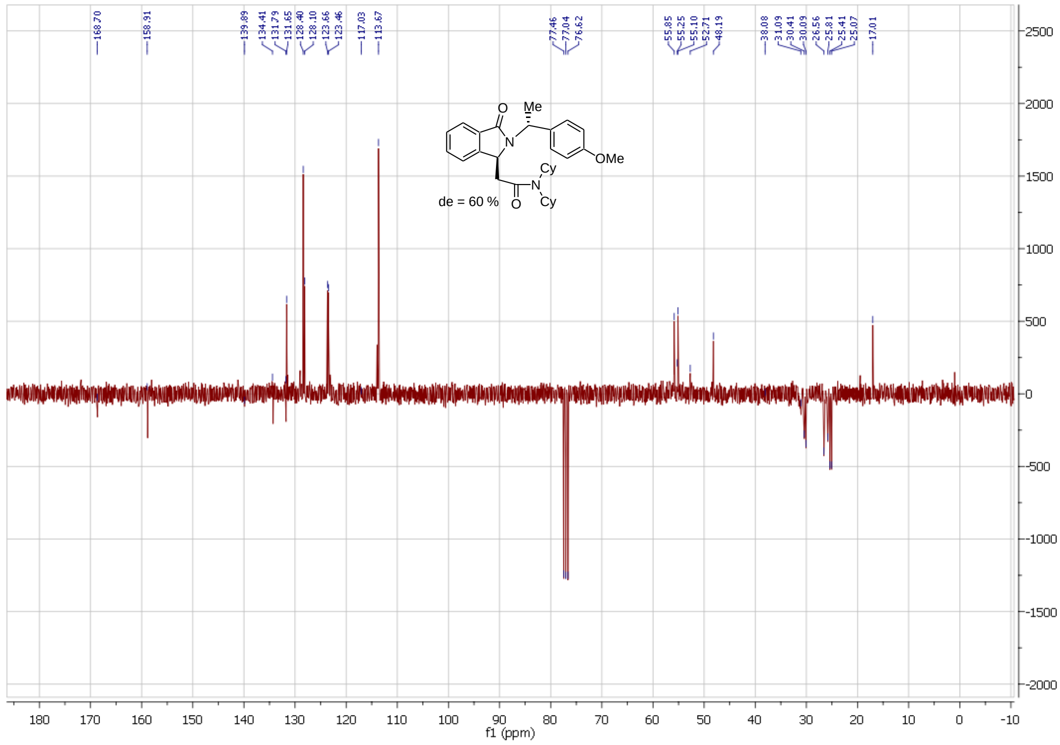

(2R,3S)-5,6-Dimethoxy-2-(1-(4-methoxyphenyl)ethyl)-3-(2-oxo-2-(pyrrolidin-1-yl)ethyl))-2,3-dihydroisoindol-1-one ((*R*)-**5**)

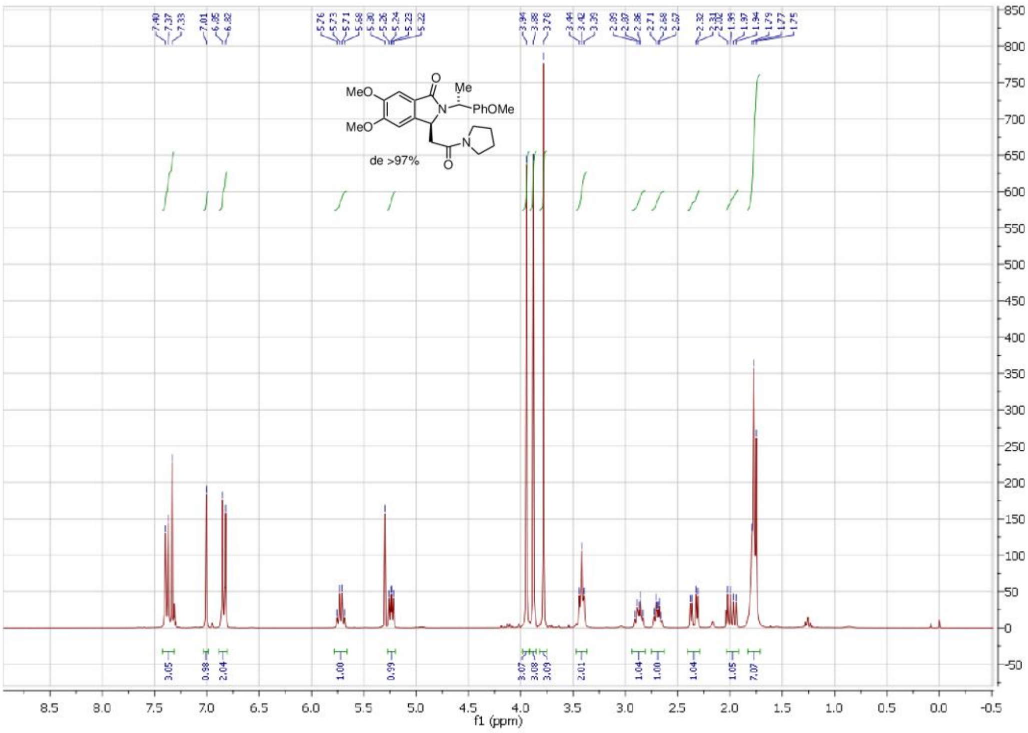

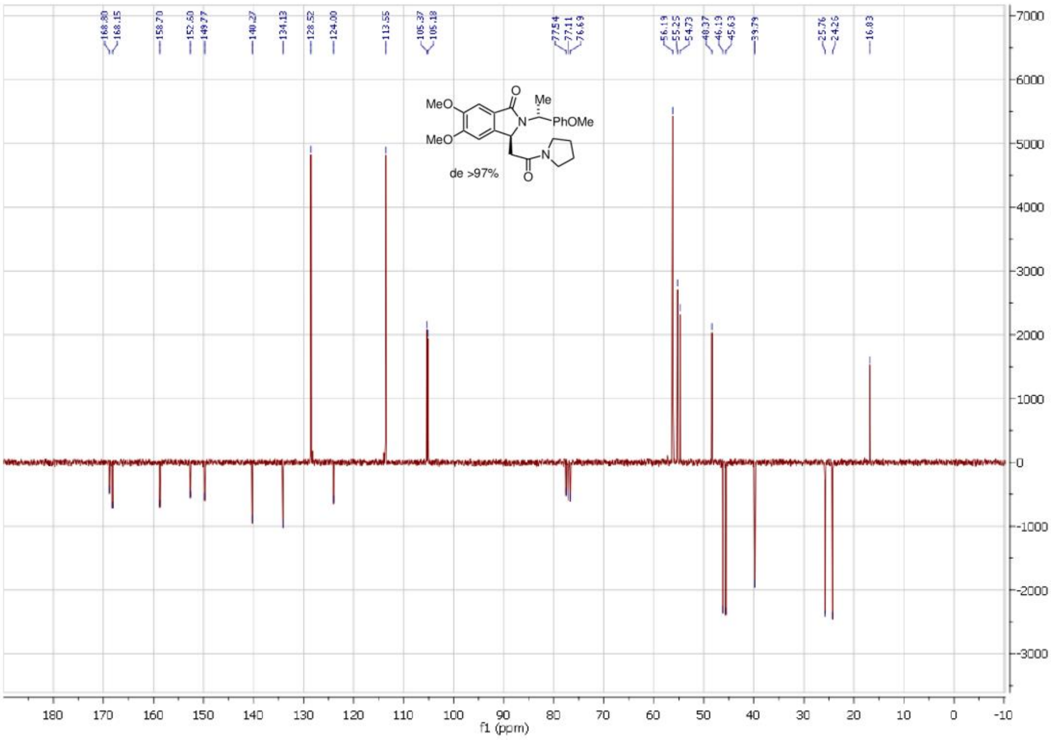

3-(2-Oxo-2-(pyrrolidin-1-yl)ethyl)-2,3-dihydroisoindol-1-one ((*S*)-**1a**)

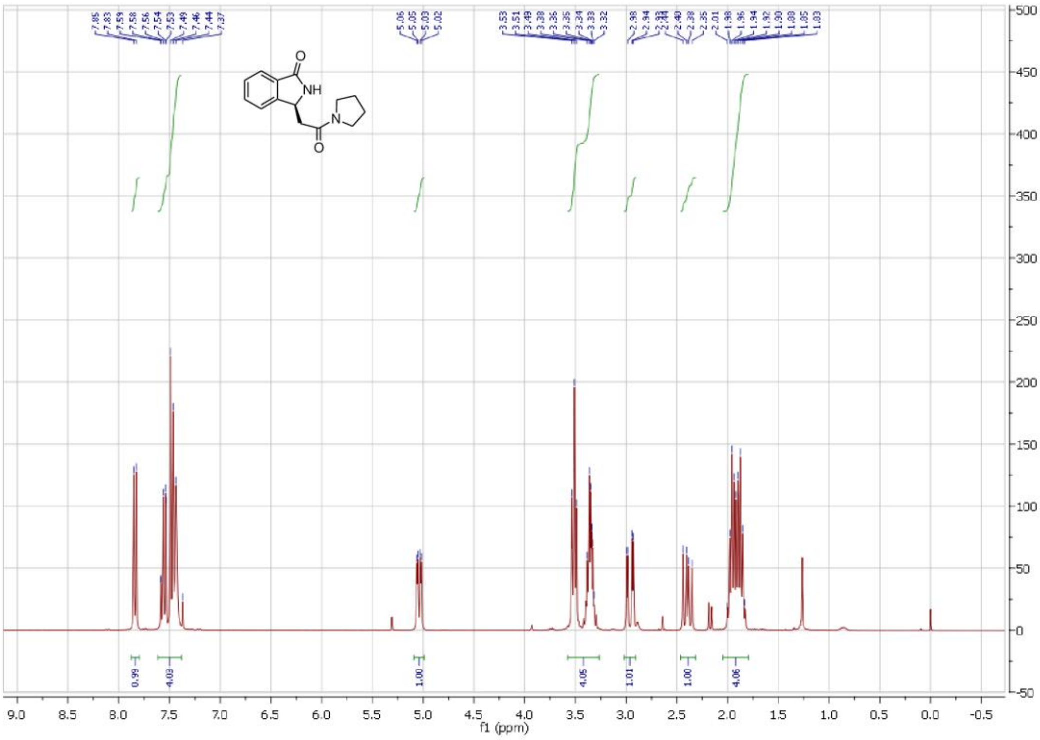

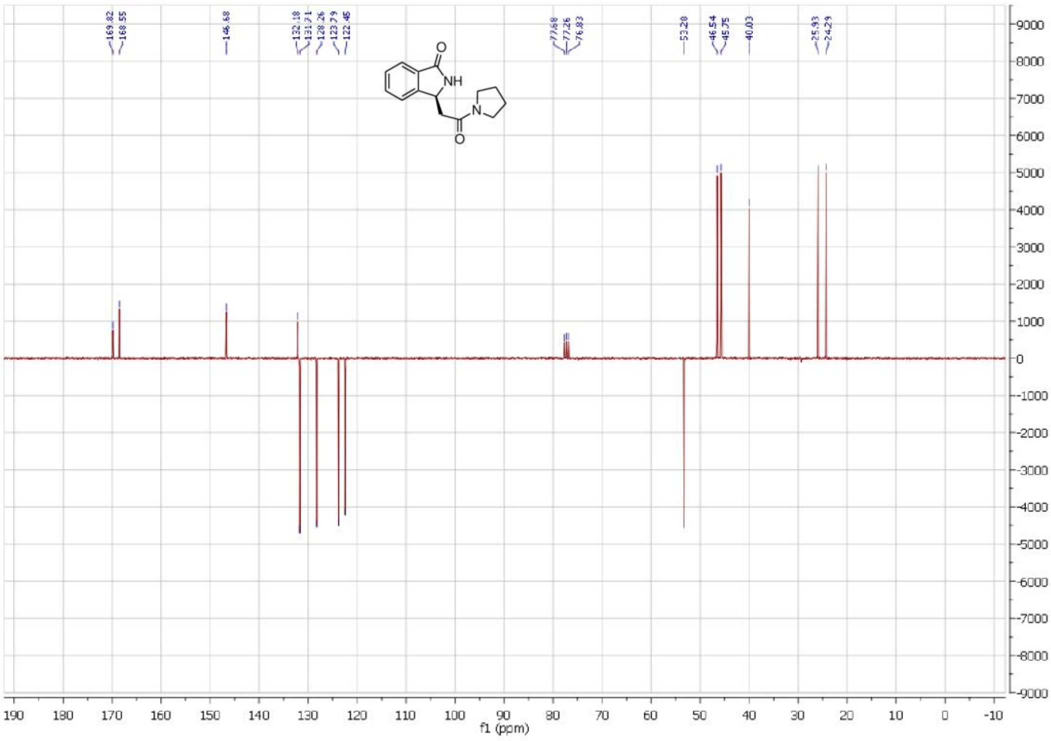

3-(2-Morpholino-2-oxoethyl)-2,3-dihydroisoindol-1-one ((*S*)-**1b**)

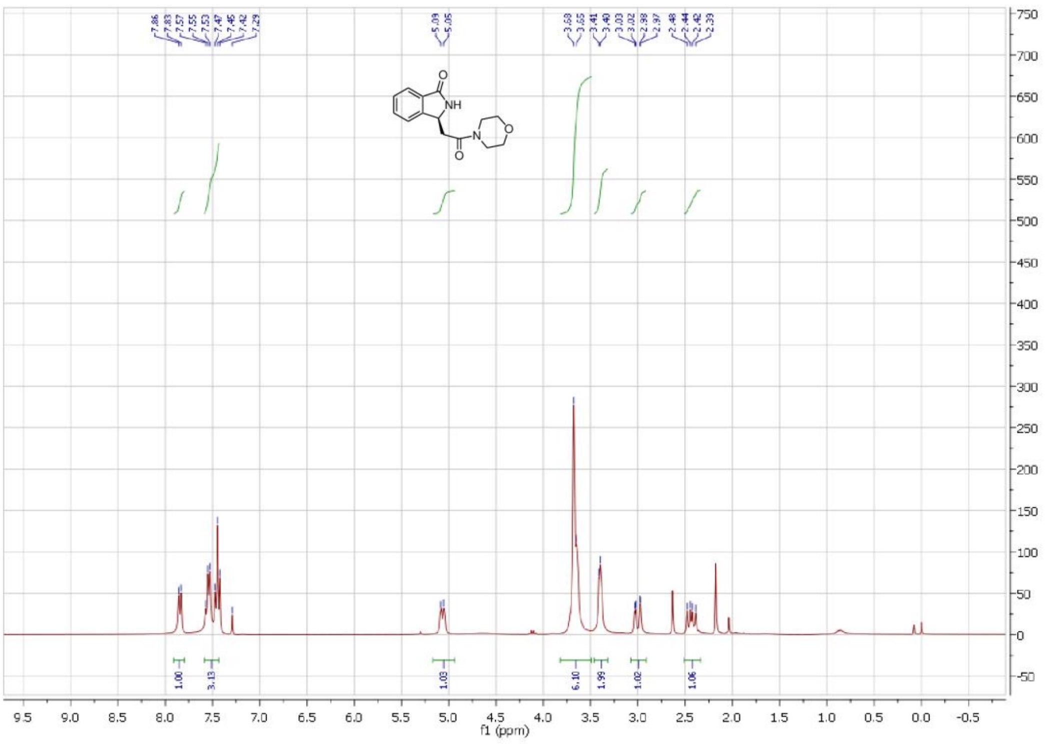

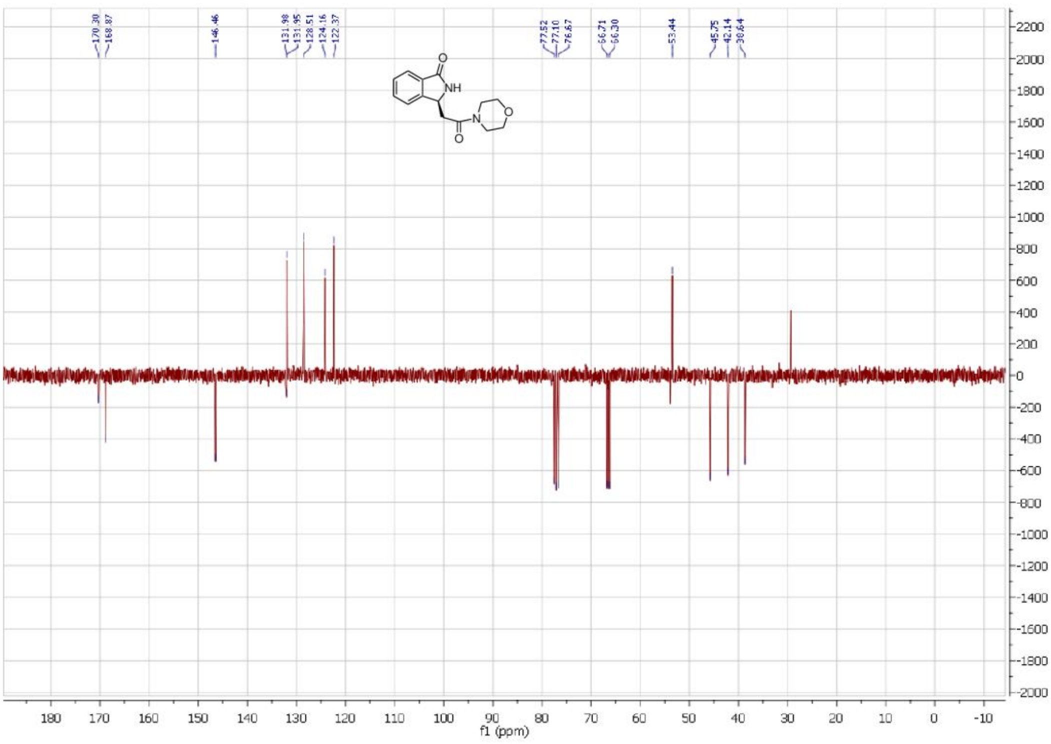

N,N-Diisopropyl-2-(3-oxoisoindolin-1-yl)acetamide ((*S*)-**1c**)

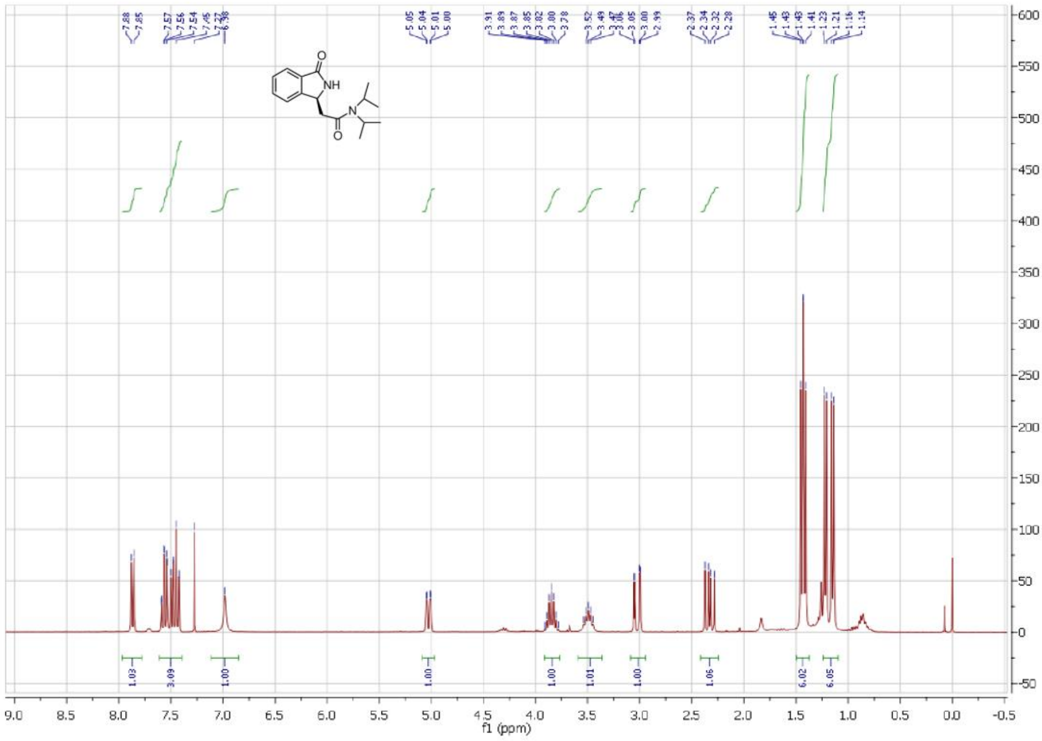

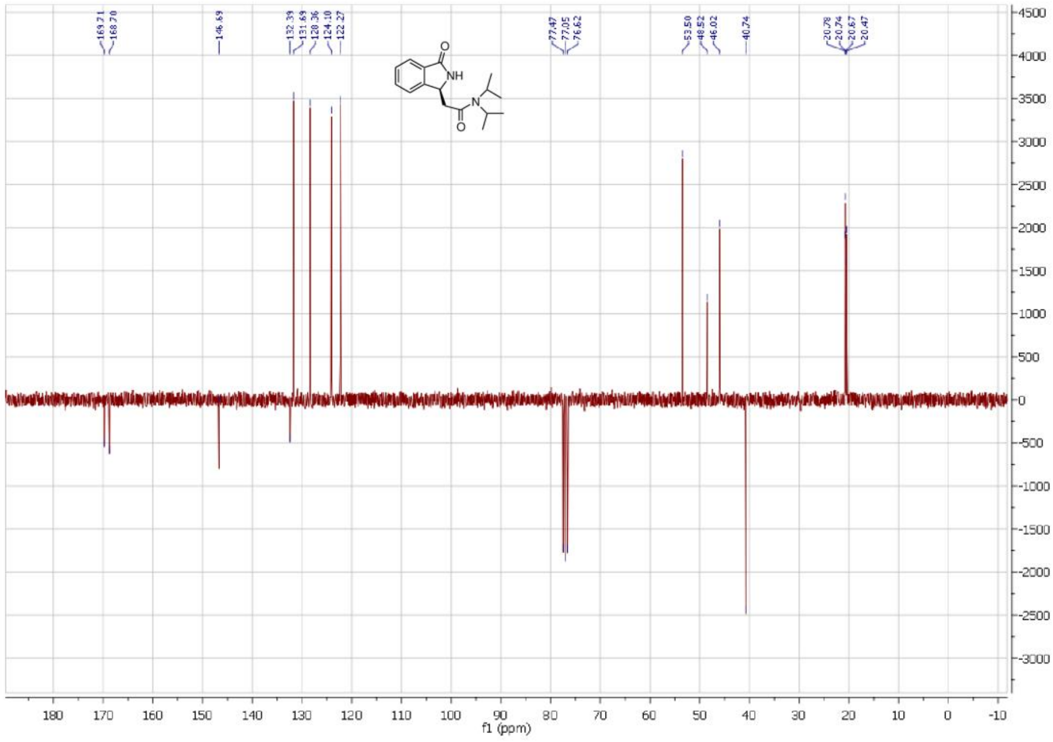

N,N-Dicyclohexyl-2-(3-oxoisoindolin-1-yl)acetamide ((*S*)-**1e**)

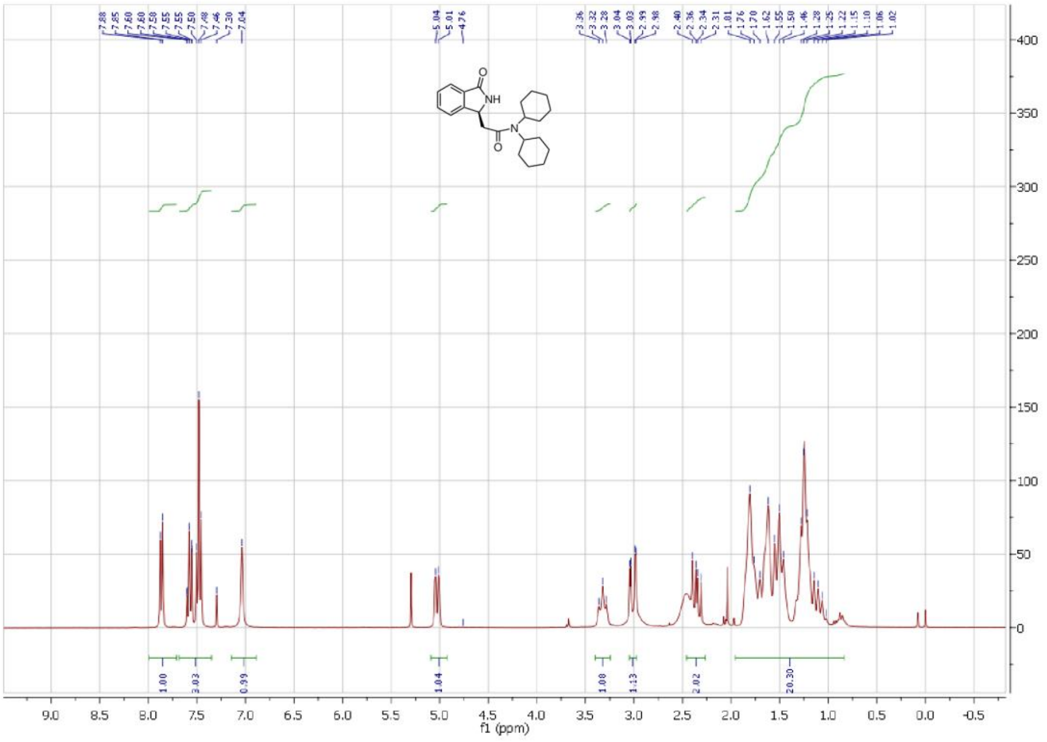

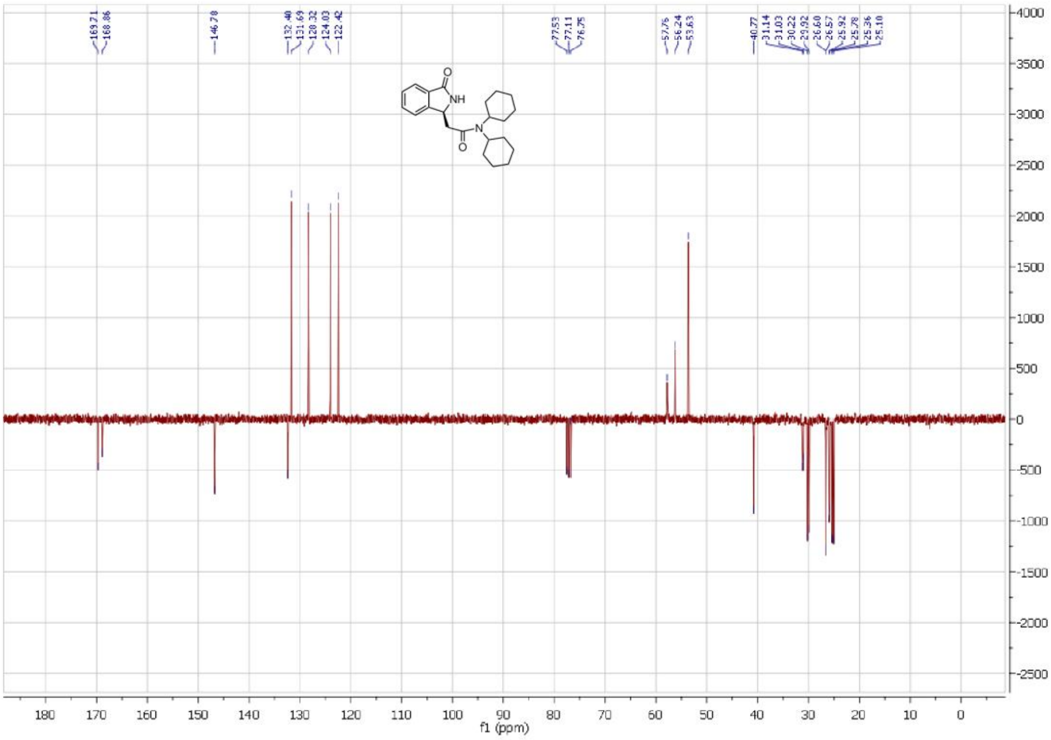

5,6-Dimethoxy-3-(2-oxo-2-(pyrrolidin-1-yl)ethyl)isoindolinone ((*S*)-**2**)

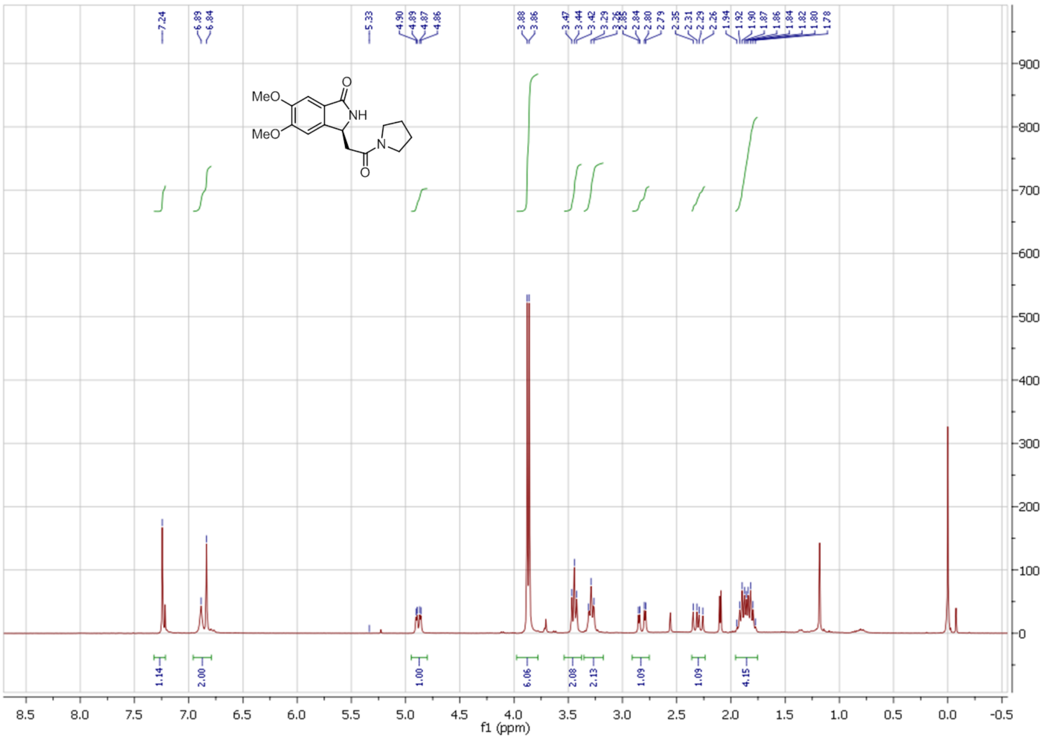

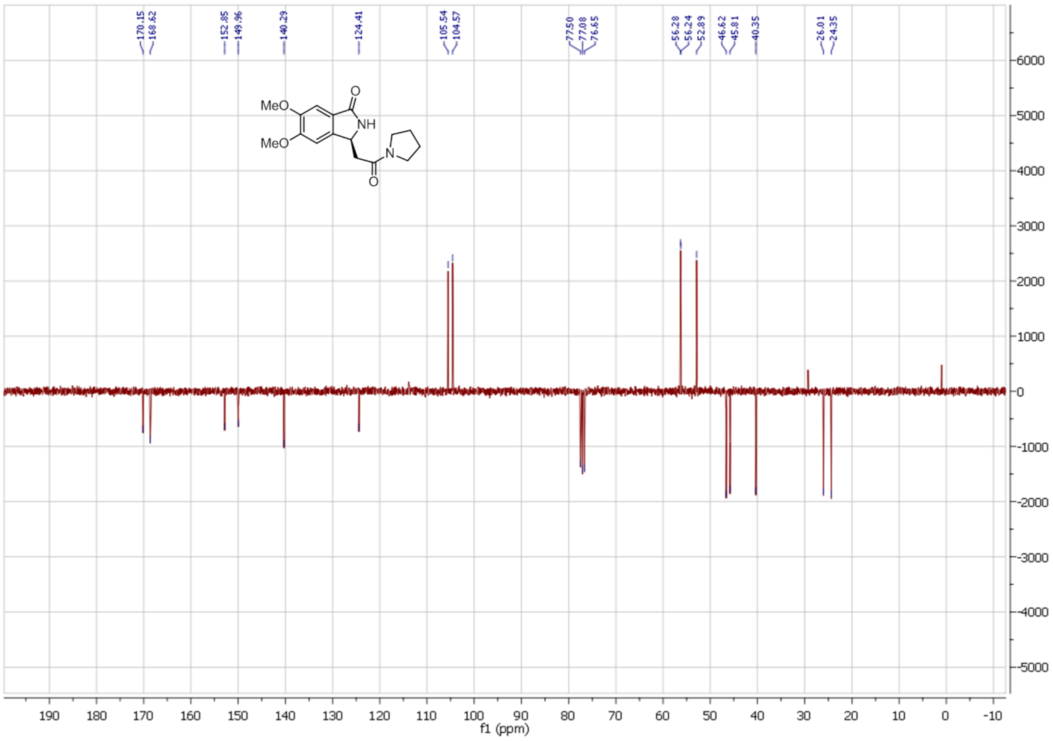

2-[(E)-3-(1,4-Dioxa-8-azaspiro[4.5]dec-8-yl)-3-oxopropenyl]benzoic acid tert-butyl ester (**28**)

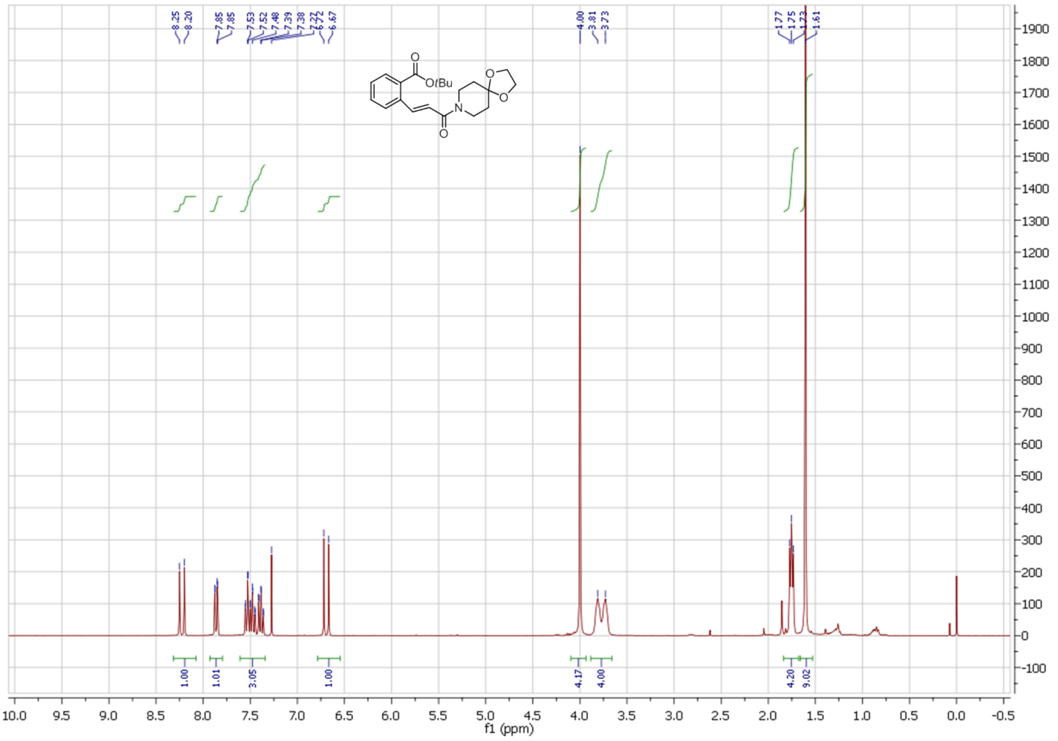

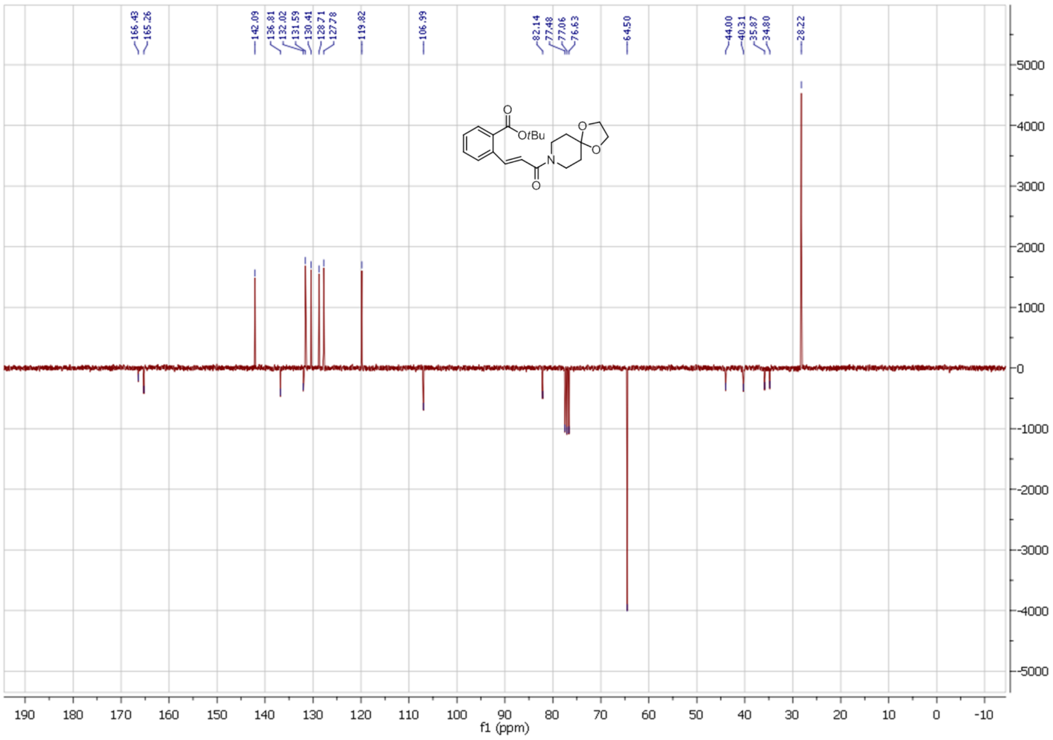

2-[(E)-3-(1,4-Dioxa-8-azaspiro[4.5]dec-8-yl)-3-oxopropenyl]benzaldehyde (**23**)

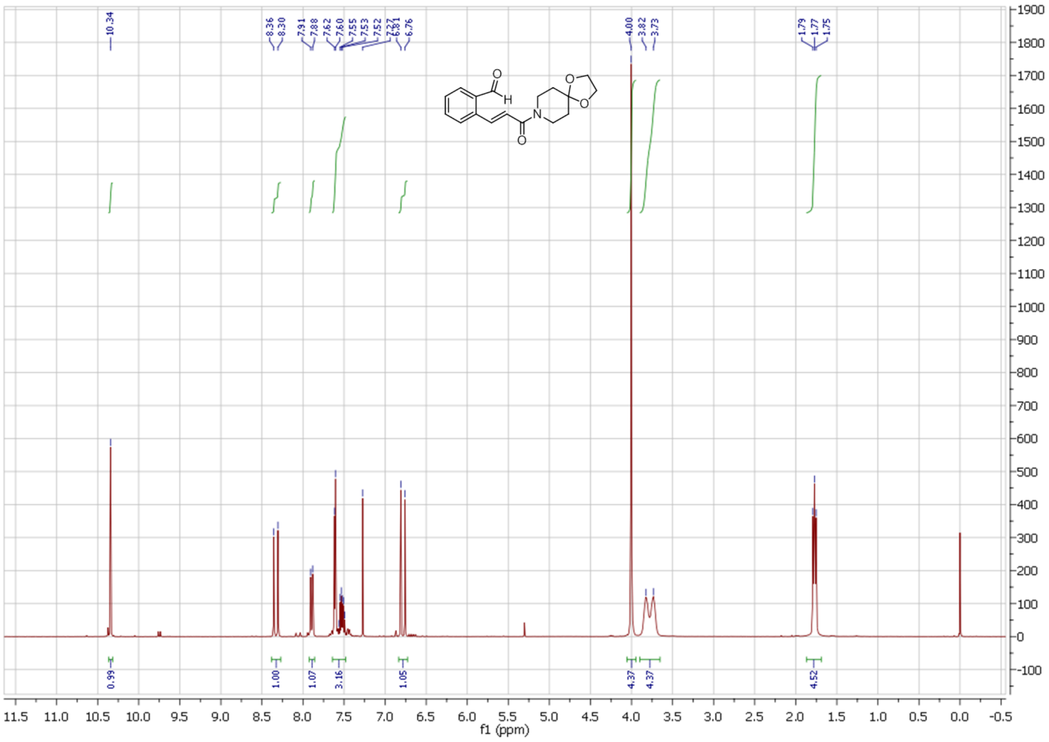

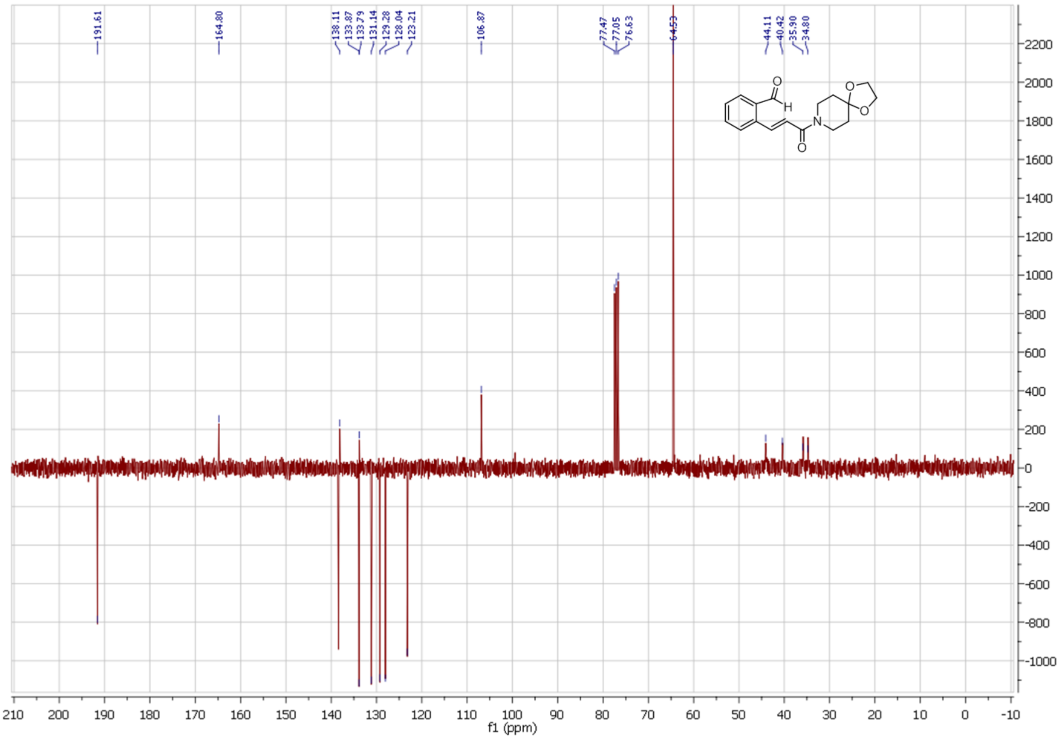

N-((R)-1-(4-Methoxyphenyl)ethyl)-2-((E)-3-oxo-(1,4-dioxa-8-azaspiro[4.5]decan-8-yl)propen-1-yl)benzamide ((*R*)-**24**)

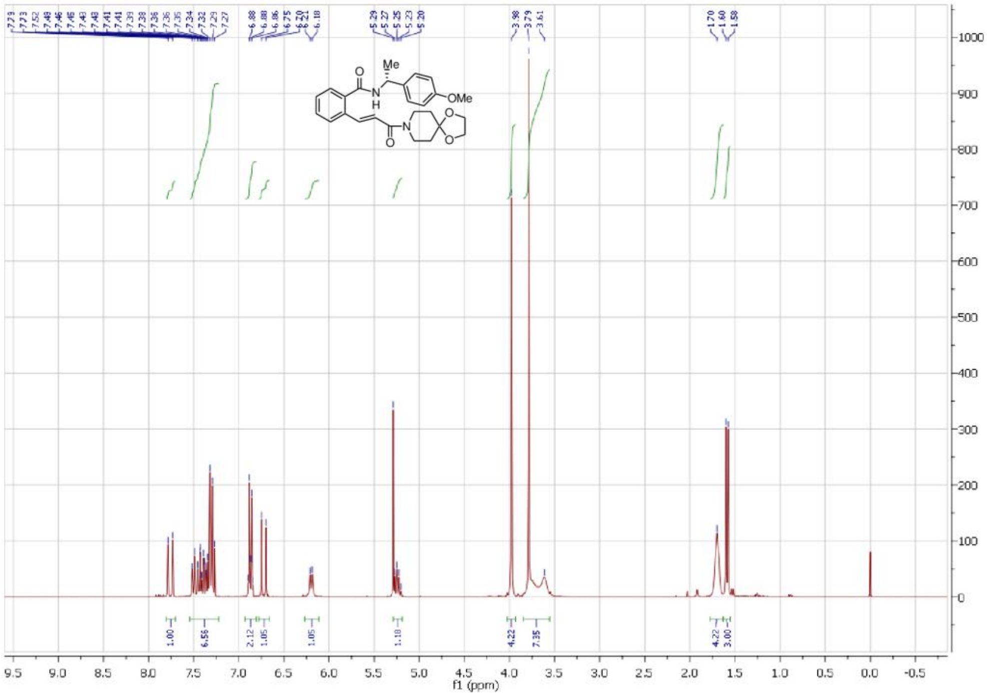

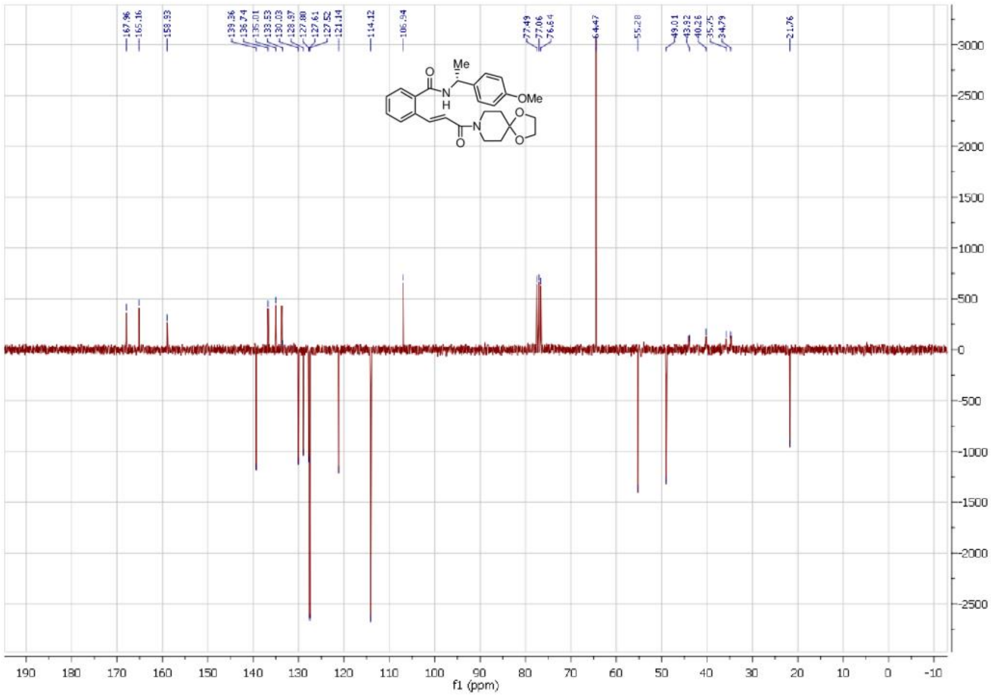

N-[(R)-1-(4-Methoxyphenyl)ethyl]-2-[(E)-3-oxo-3-(4-oxopiperidin-1-yl)propenyl]benzamide ((*R*)-**29**)

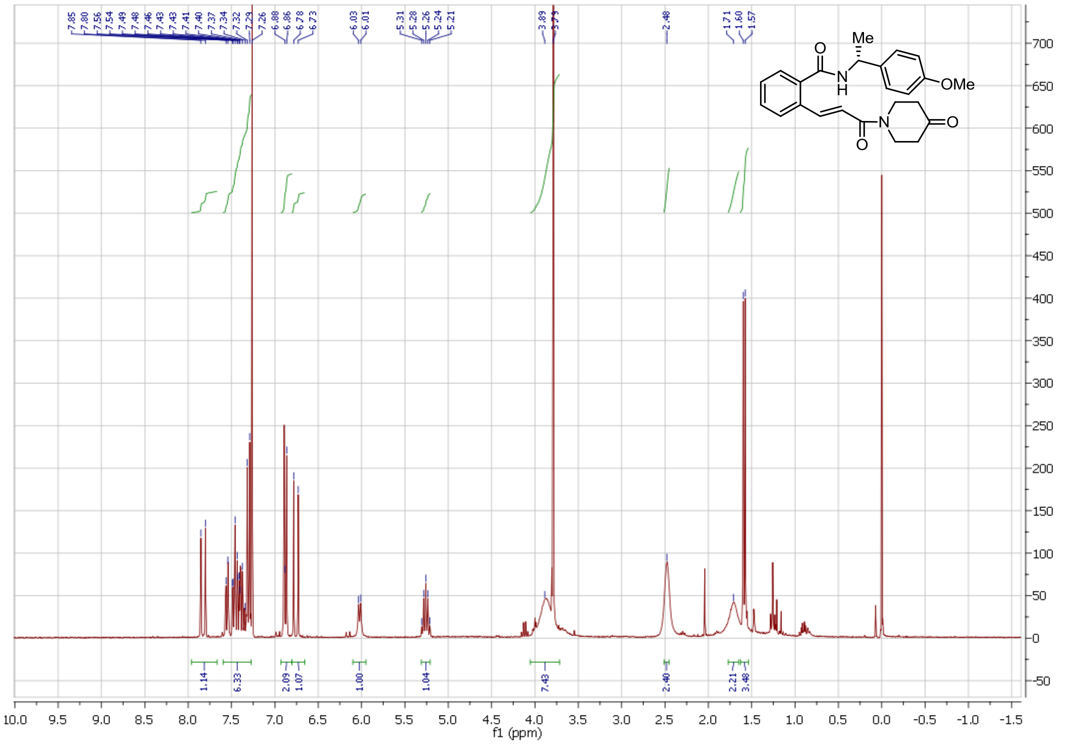

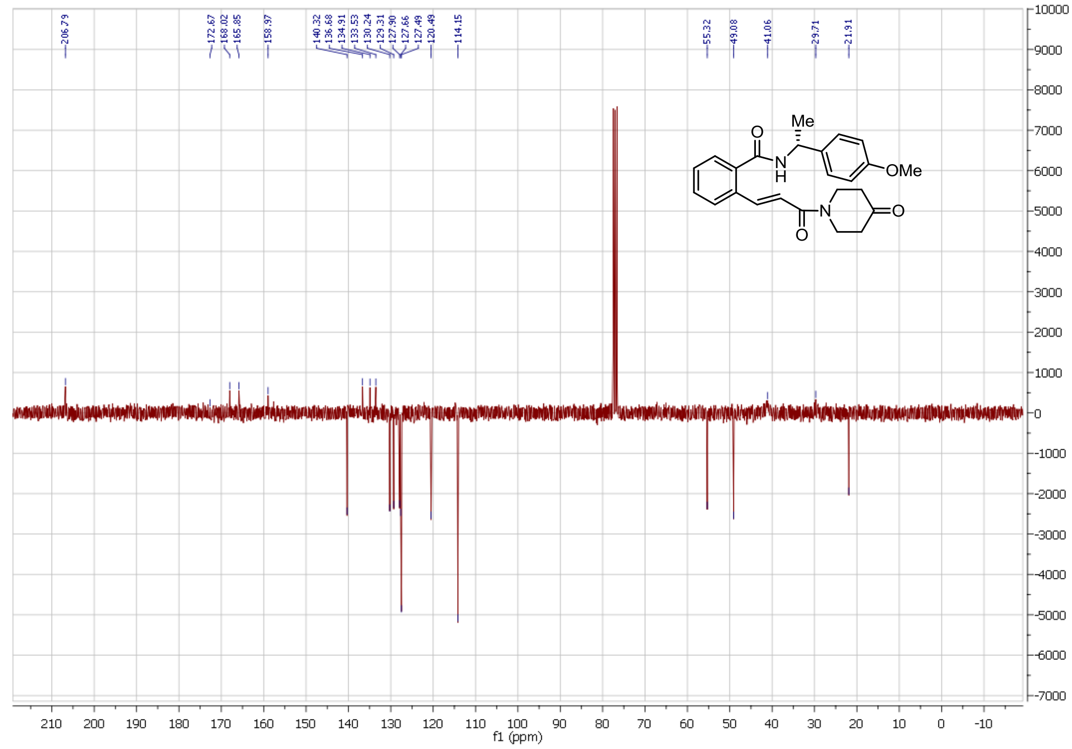

(2R,3S)-2-(1-4-Methoxyphenyl)ethyl)-3-(2-oxo-2-(1,4-dioxa-8-azaspiro[4.5]decan-8-yl)ethyl-2,3-dihydroisoindol-1-one ((*R*)-**25**)

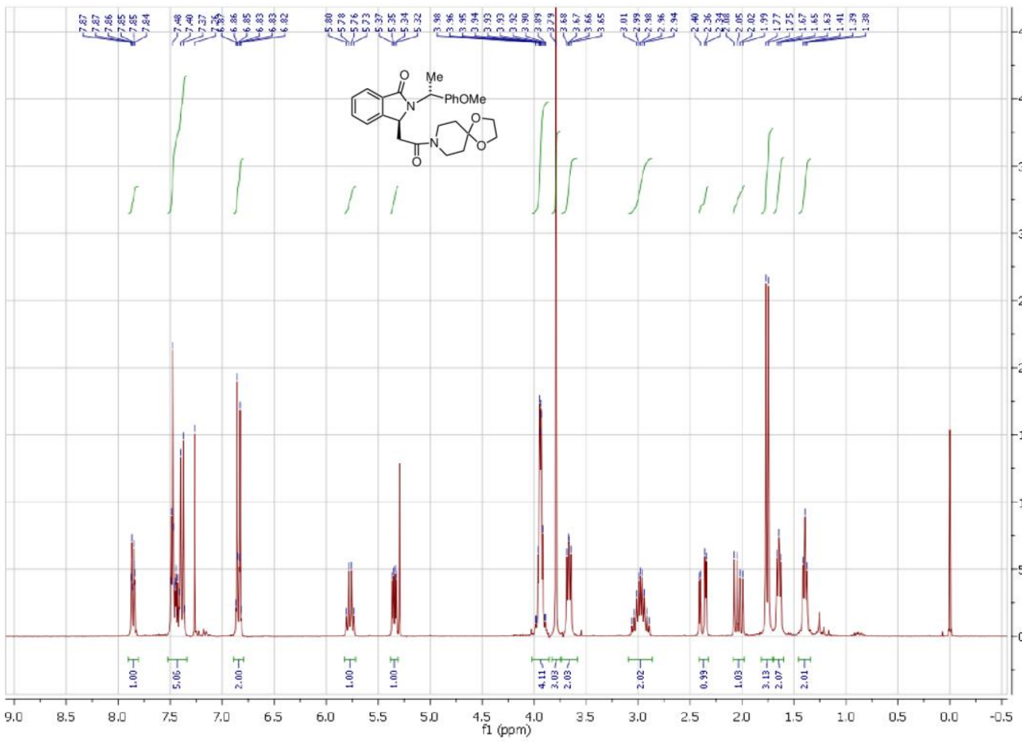

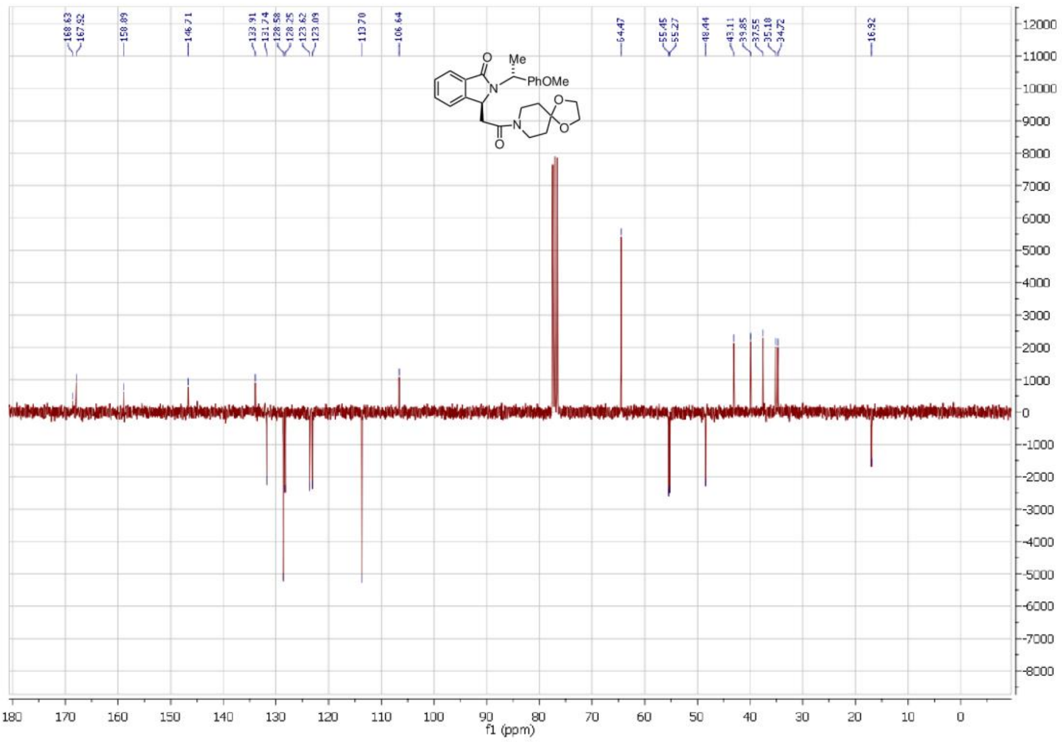

(3S)-3-(2-oxo-2-(1,4-dioxa-8-azaspiro[4.5]decan-8-yl)ethyl)-2,3-dihydro-isoindol-1-one ((*S*)-**26**)

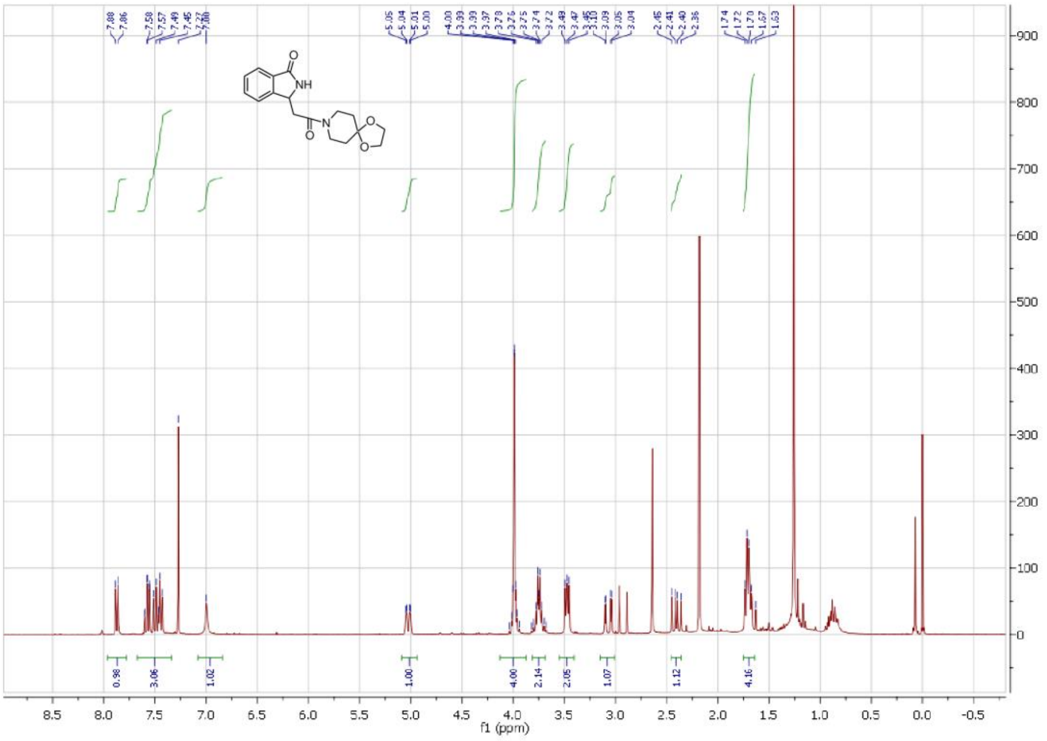

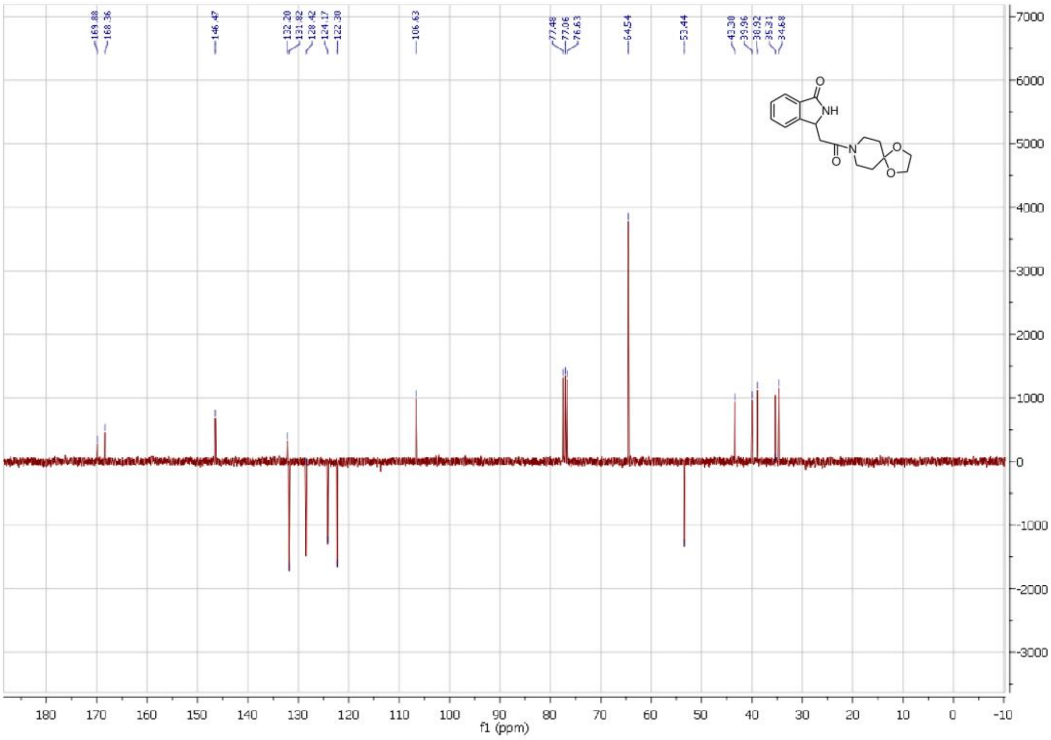

(S)-3-(2-oxo-2-(1,4-dioxa-8-azaspiro[4.5]decan-8-yl)ethyl)-2-(pyridin-2-yl)isoindolin-1-one ((*S*)-**27**)

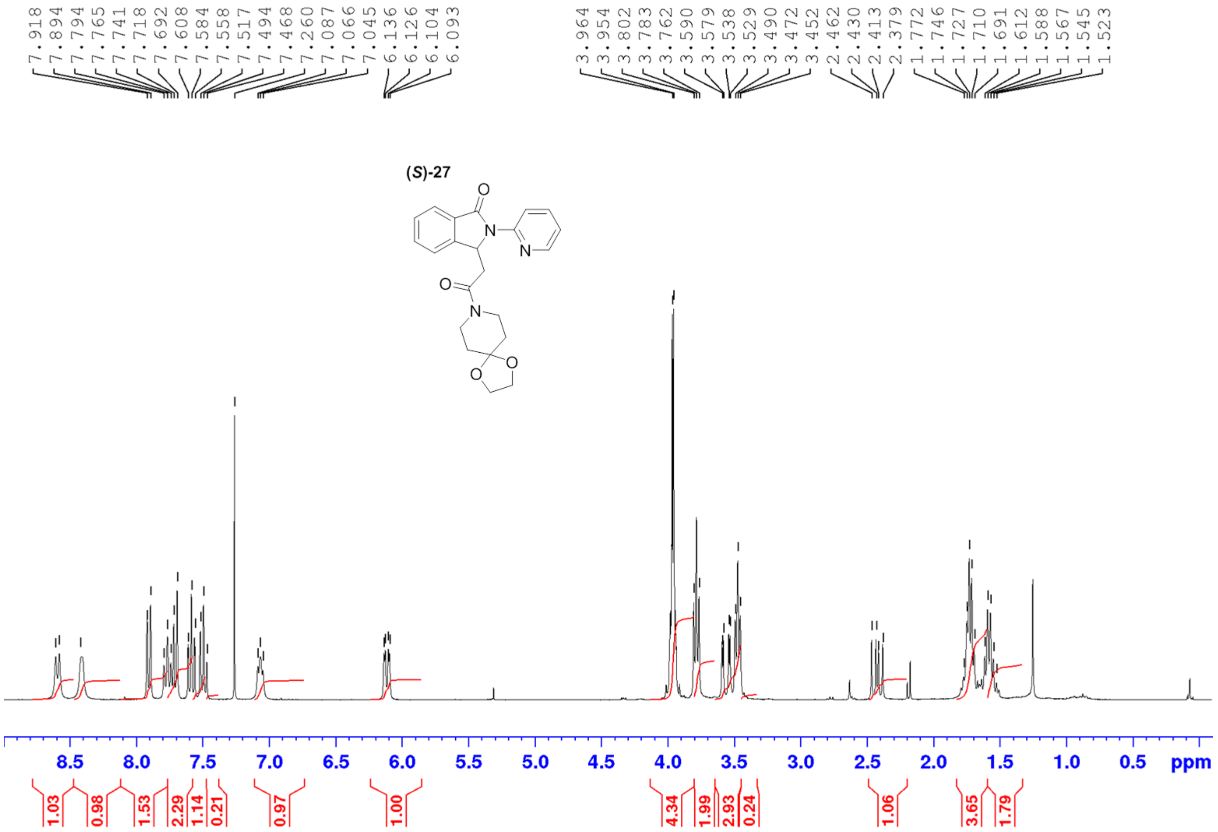

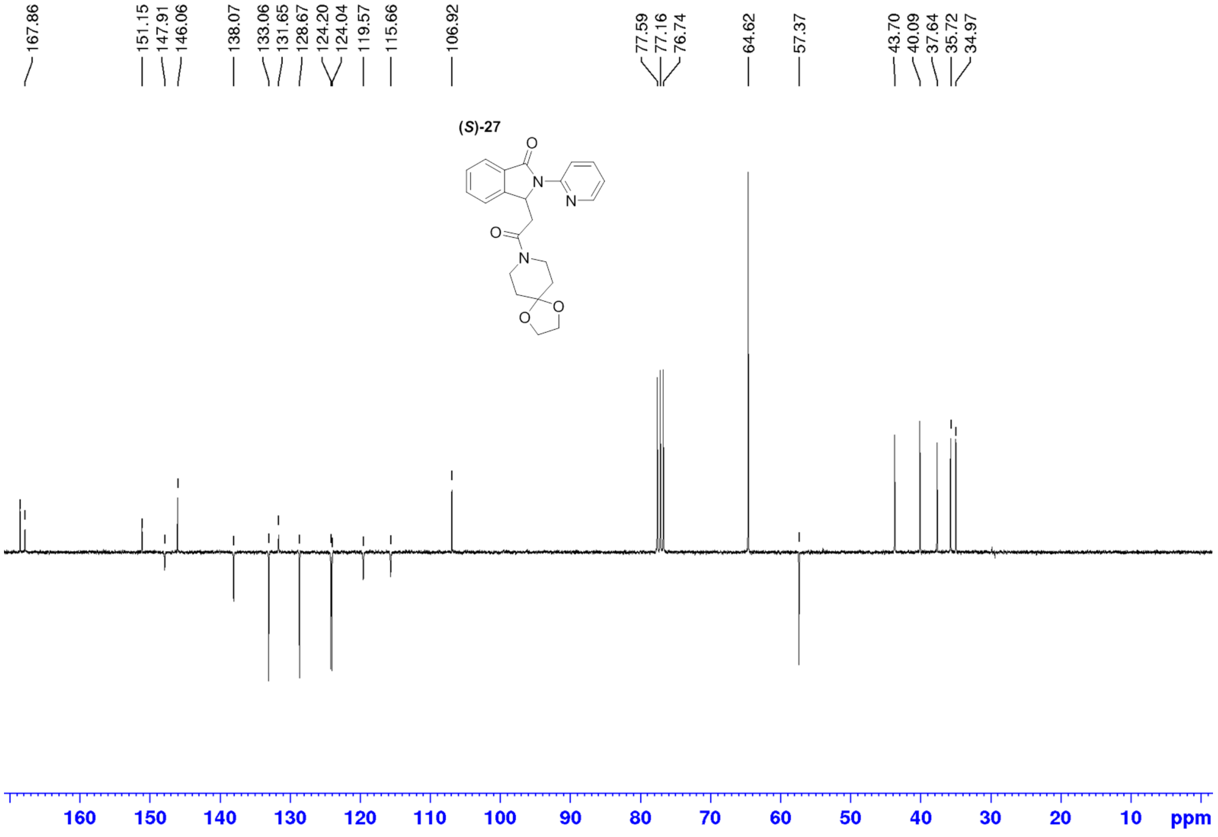

5. HPLC for compounds

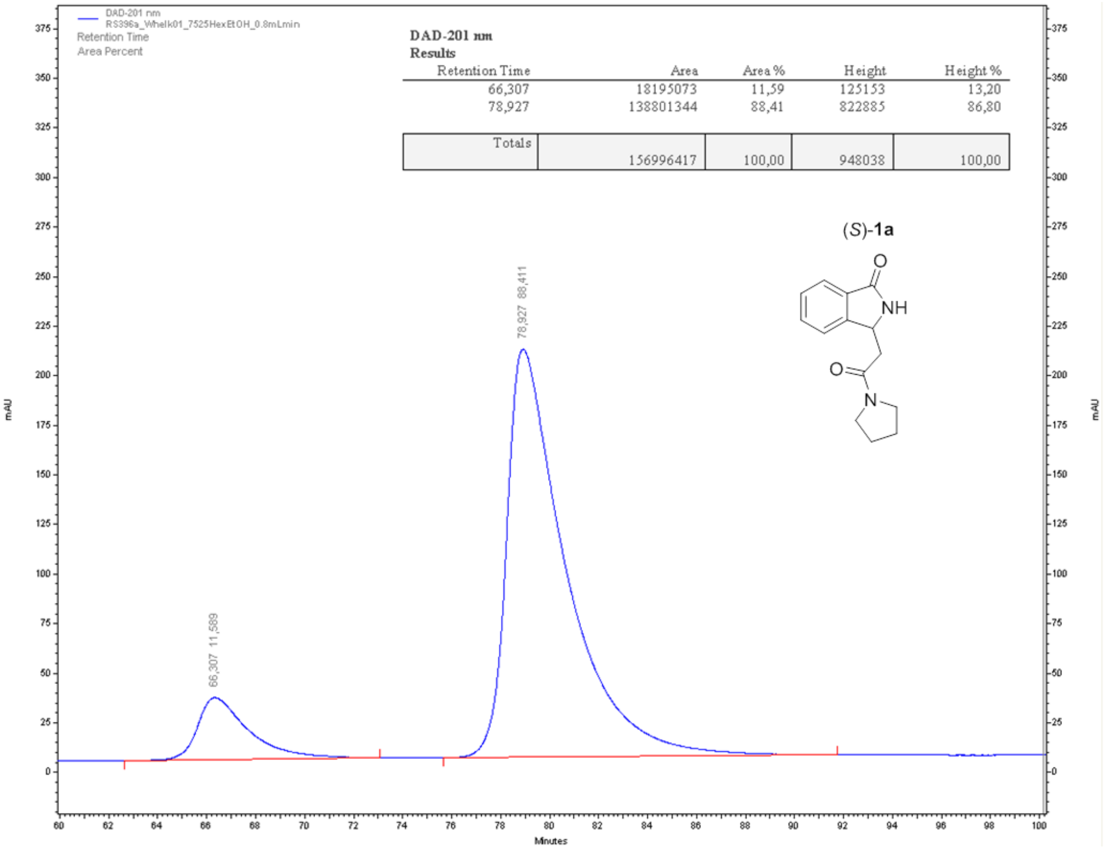

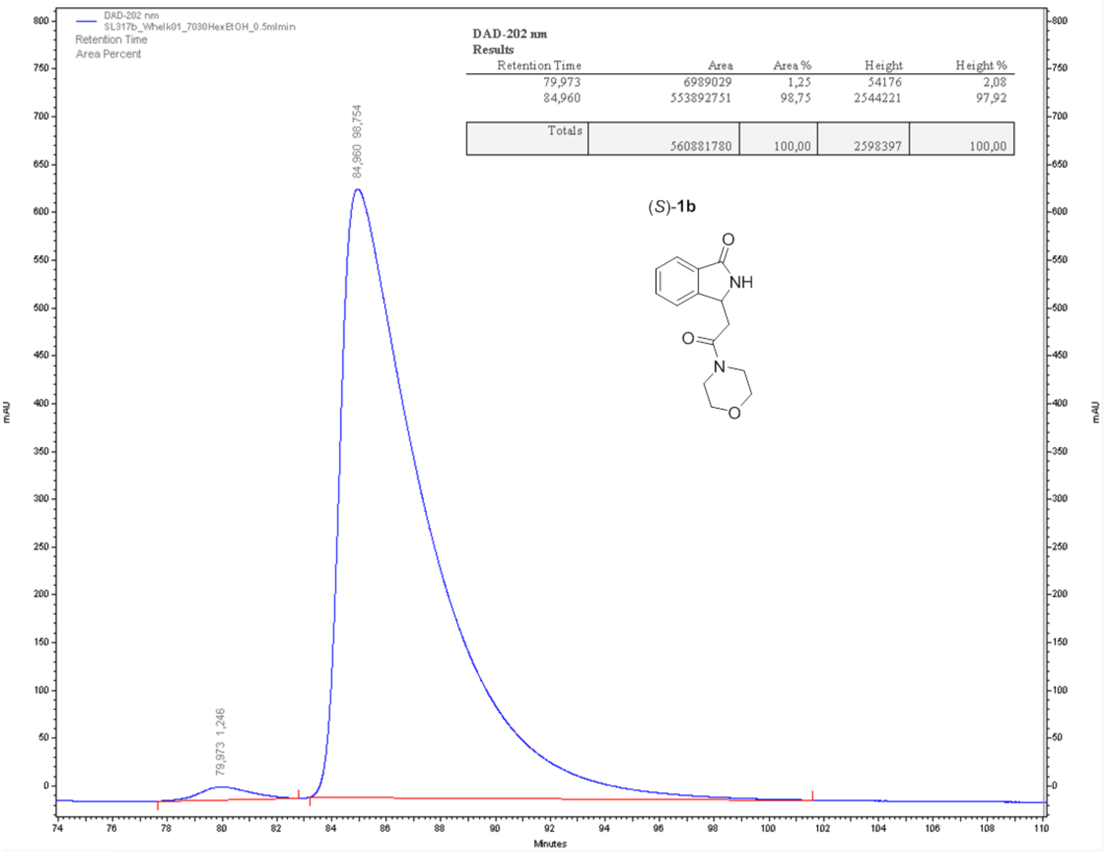

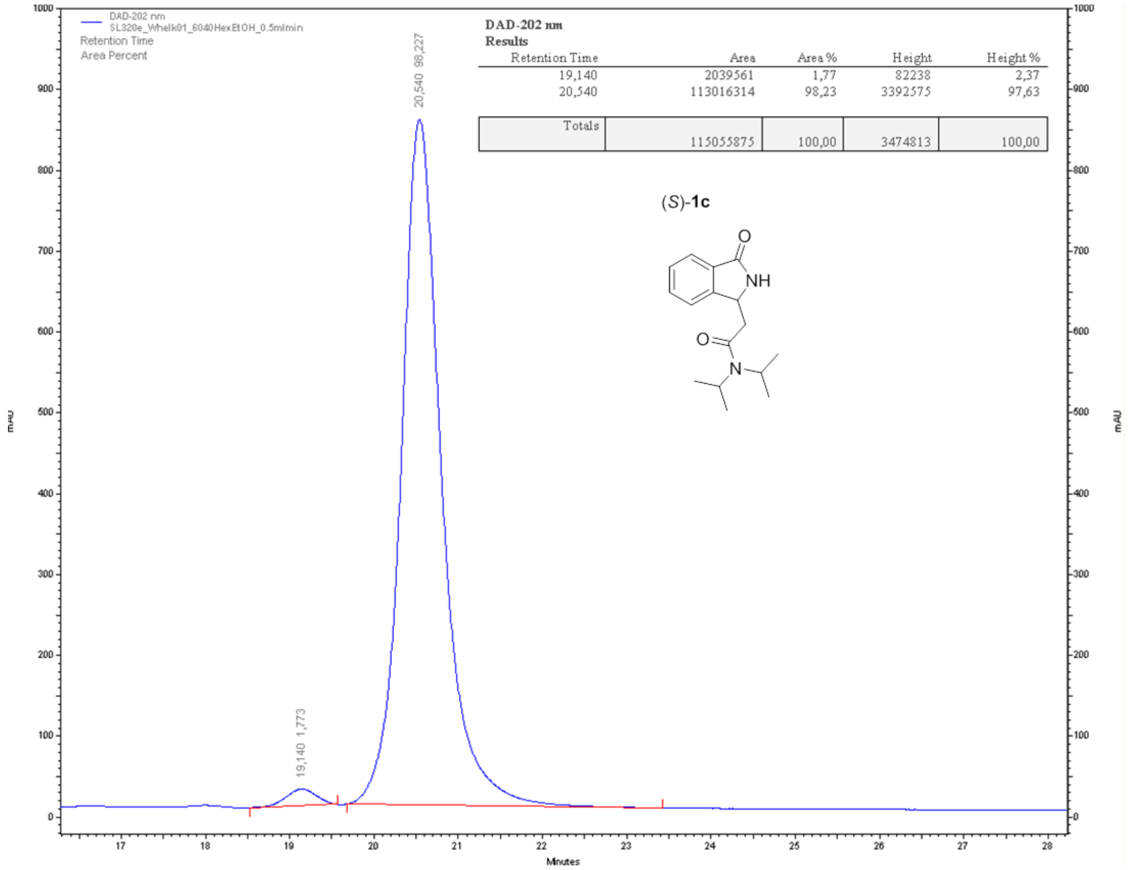
DAD-202 nm
SL320e_Whelk01_6040HexEtOH_0.5mlmin
Retention Time
Area Percent
20,540 98,227
19,140 1,773
DAD-202 nm
Results
Retention Time | Area | Area % | Height | Height %
19,140 | 2039561 | 1,77 | 82238 | 2,37
20,540 | 113016314 | 98,23 | 3392575 | 97,63
Totals | 115055875 | 100,00 | 3474813 | 100,00
(S)-1c
O
NH
O
N
mAU
Minutes

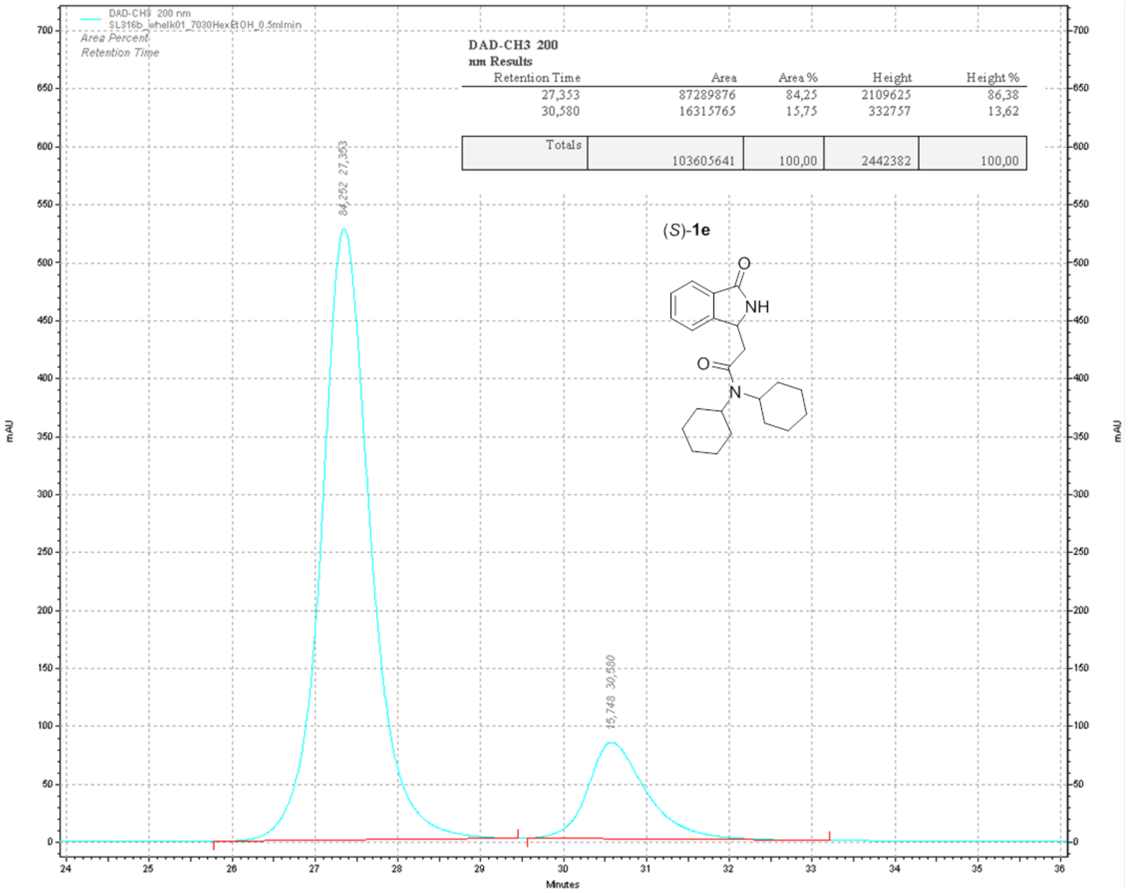
DAD-CH3 200 nm
SL316b_whelk01_7030HexEtOH_0.5mlmin
Area Percent
Retention Time
84,252 27,353
15,748 30,580
DAD-CH3 200 nm Results
Retention Time | Area | Area % | Height | Height %
27,353 | 87289876 | 84,25 | 2109625 | 86,38
30,580 | 16315765 | 15,75 | 332757 | 13,62
Totals | 103605641 | 100,00 | 2442382 | 100,00
(S)-1e
O
NH
O
N
mAU
Minutes

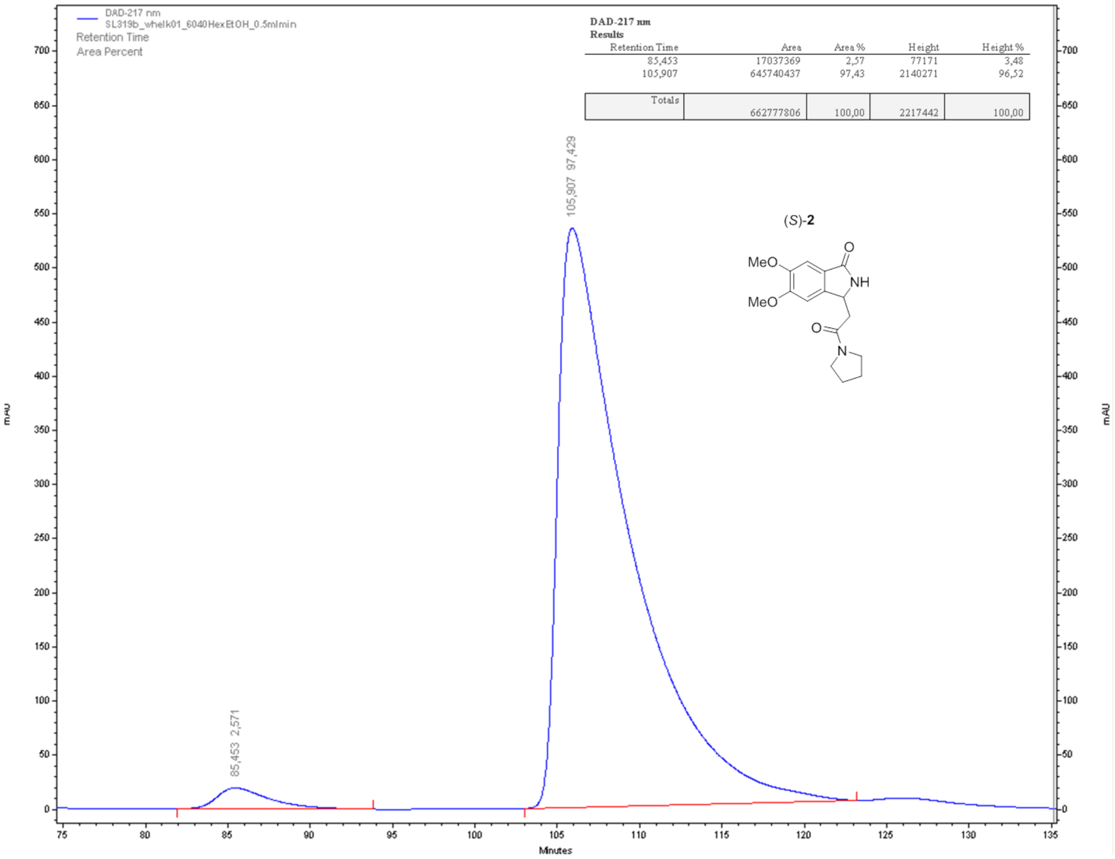

DAD-217 nm
Results

Retention Time	Area	Area %	Height	Height %
85,453	17037369	2,57	77171	3,48
105,907	645740437	97,43	2140271	96,52
Totals	662777806	100,00	2217442	100,00

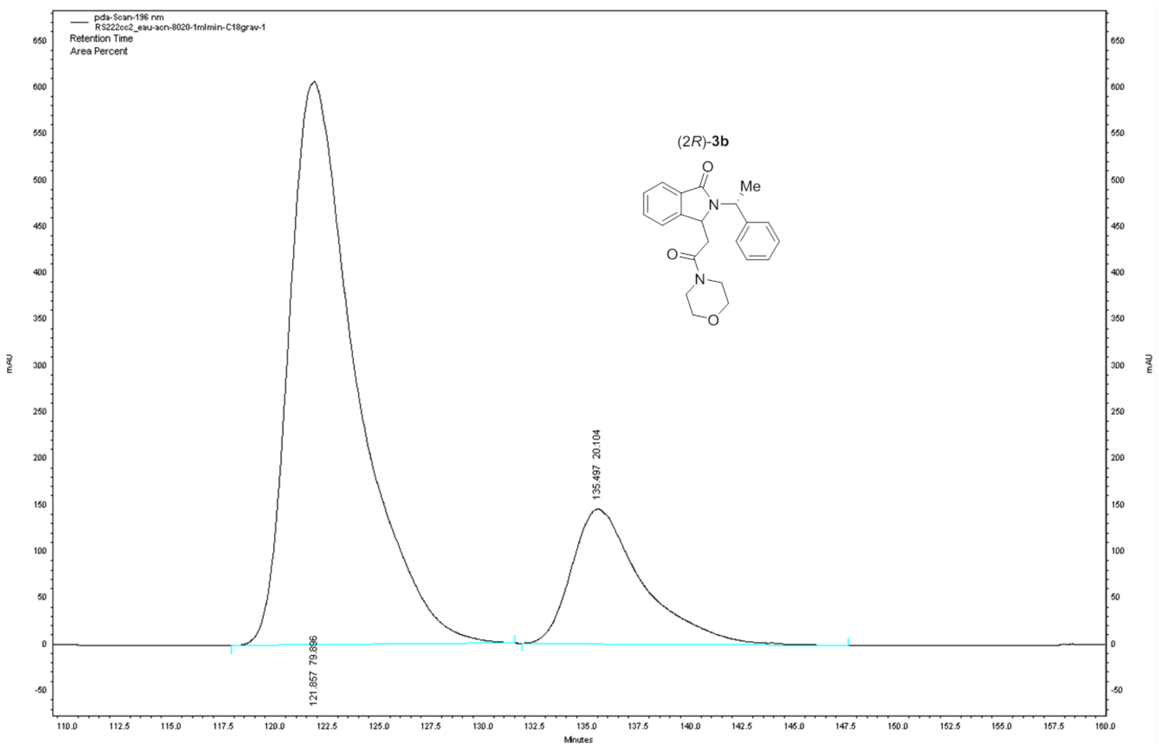
pda-Scan-196 nm
RS222cc2_eau-acn-8020-1mlmin-C18grav-1
Retention Time
Area Percent
(2R)-3b
135.497 20.104
121.857 79.896
mAU
Minutes

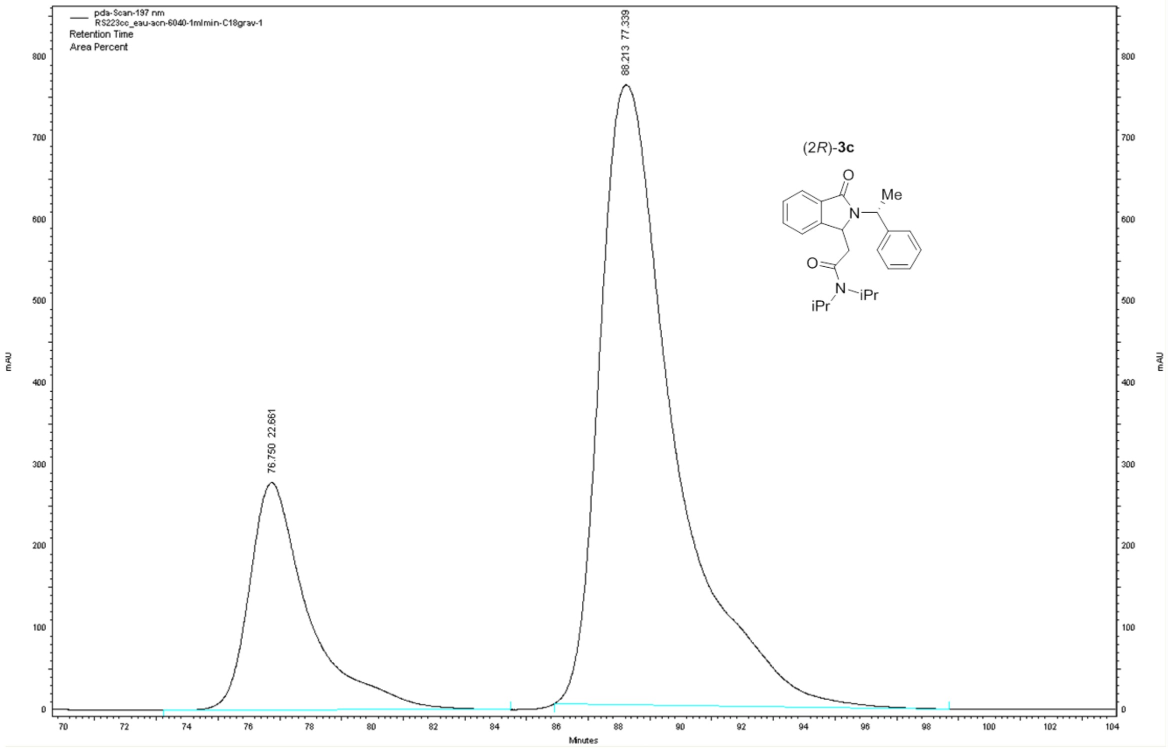
pda-Scan-197 nm
RS223cc_eau-acn-6040-1mlmin-C18grav-1
Retention Time
Area Percent
88.213 77.339
(2R)-3c
76.750 22.661
mAU
Minutes

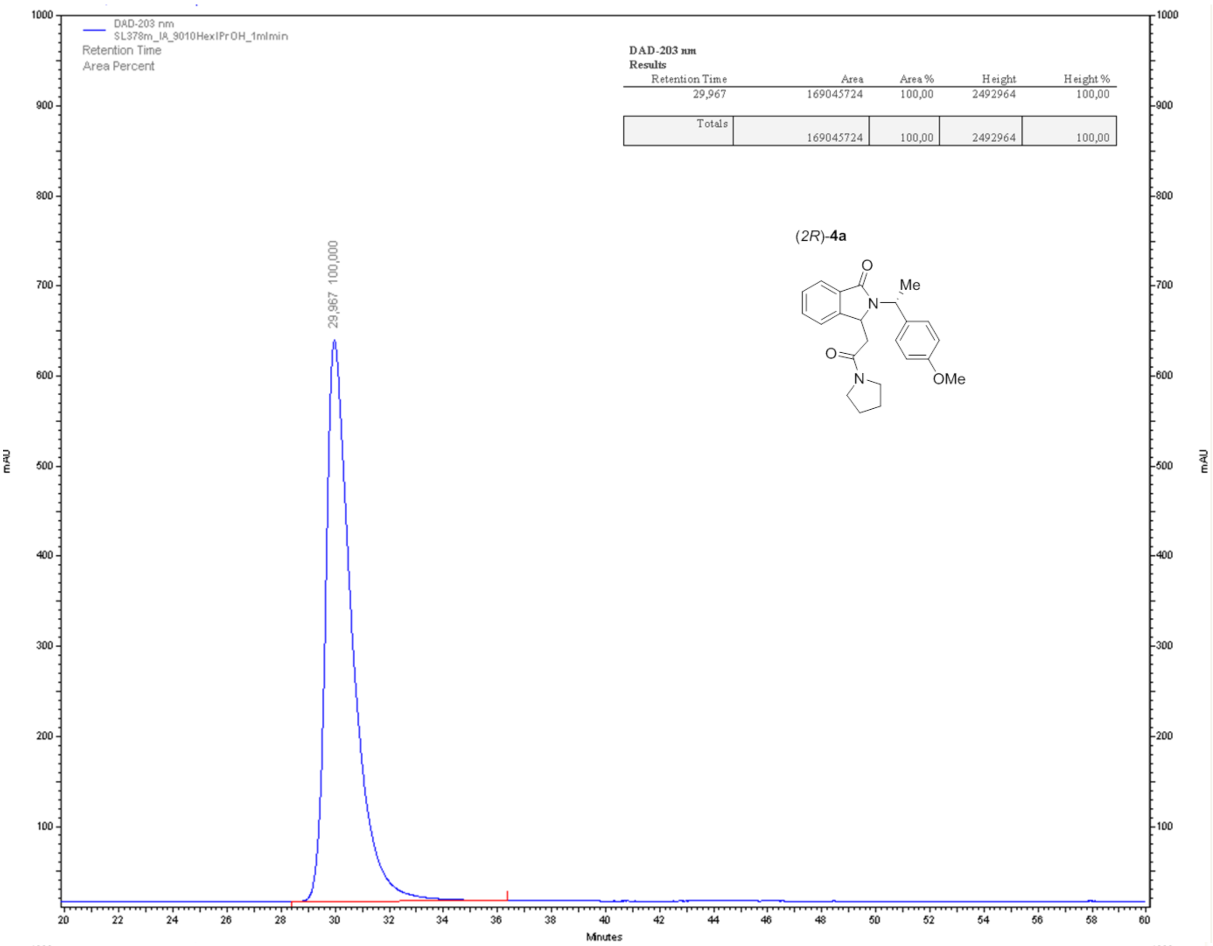

DAD-203 nm
Results

Retention Time	Area	Area %	Height	Height %
29,967	169045724	100,00	2492964	100,00
Totals	169045724	100,00	2492964	100,00

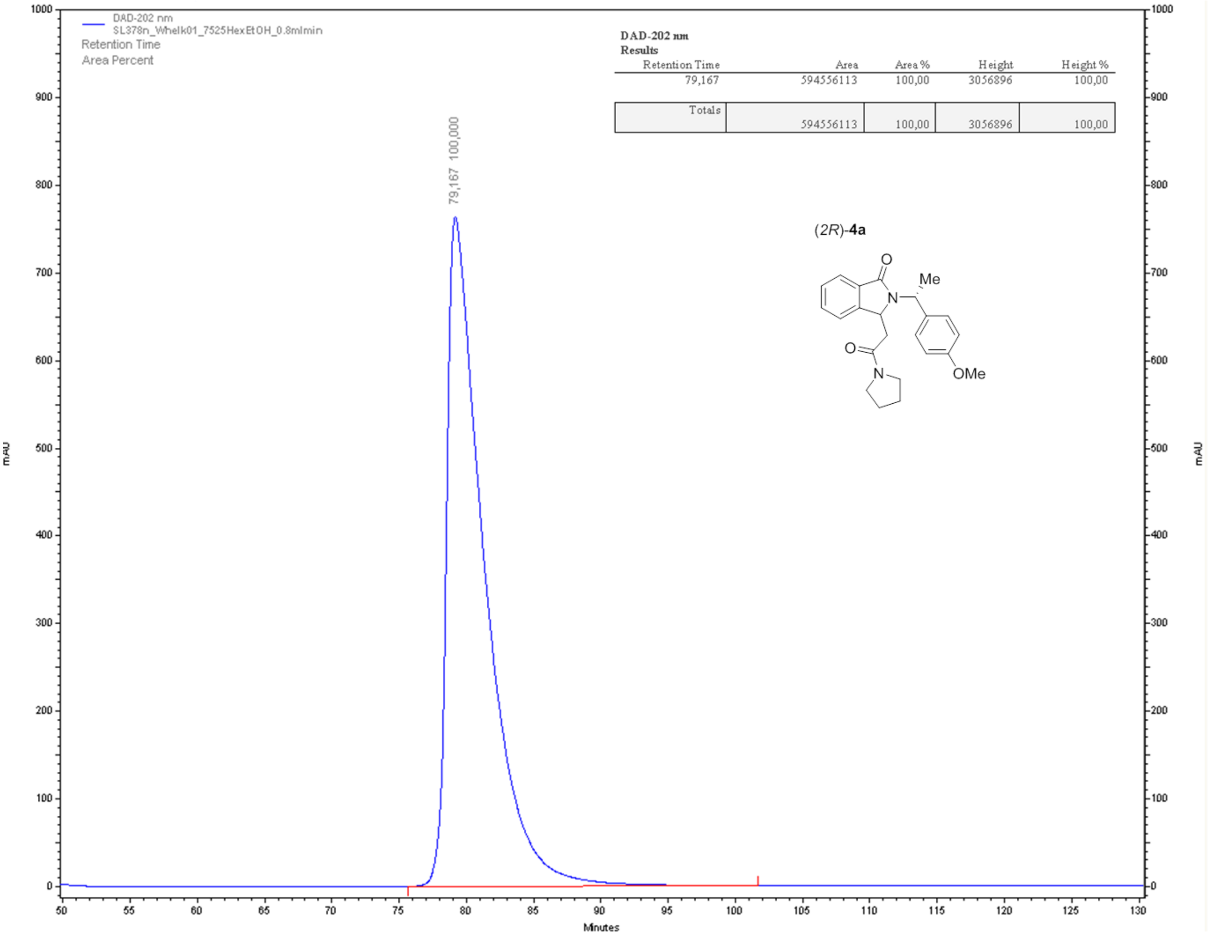

DAD-202 nm
Results

Retention Time	Area	Area %	Height	Height %
79,167	594556113	100,00	3056896	100,00
Totals	594556113	100,00	3056896	100,00

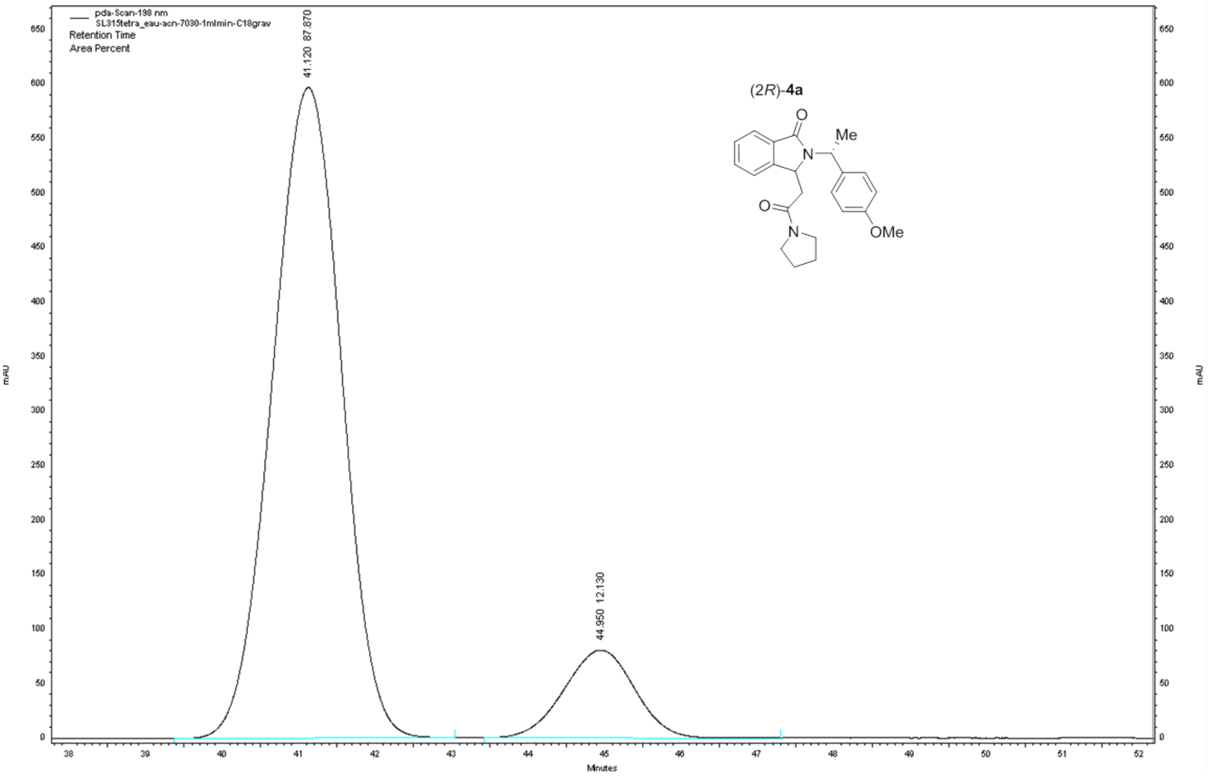
pda-Scan-198 nm
SL315tetra_eau-acn-7030-1mlmin-C18grav
Retention Time
Area Percent
41.120 87.870
44.950 12.130
(2R)-4a
Minutes
mAU

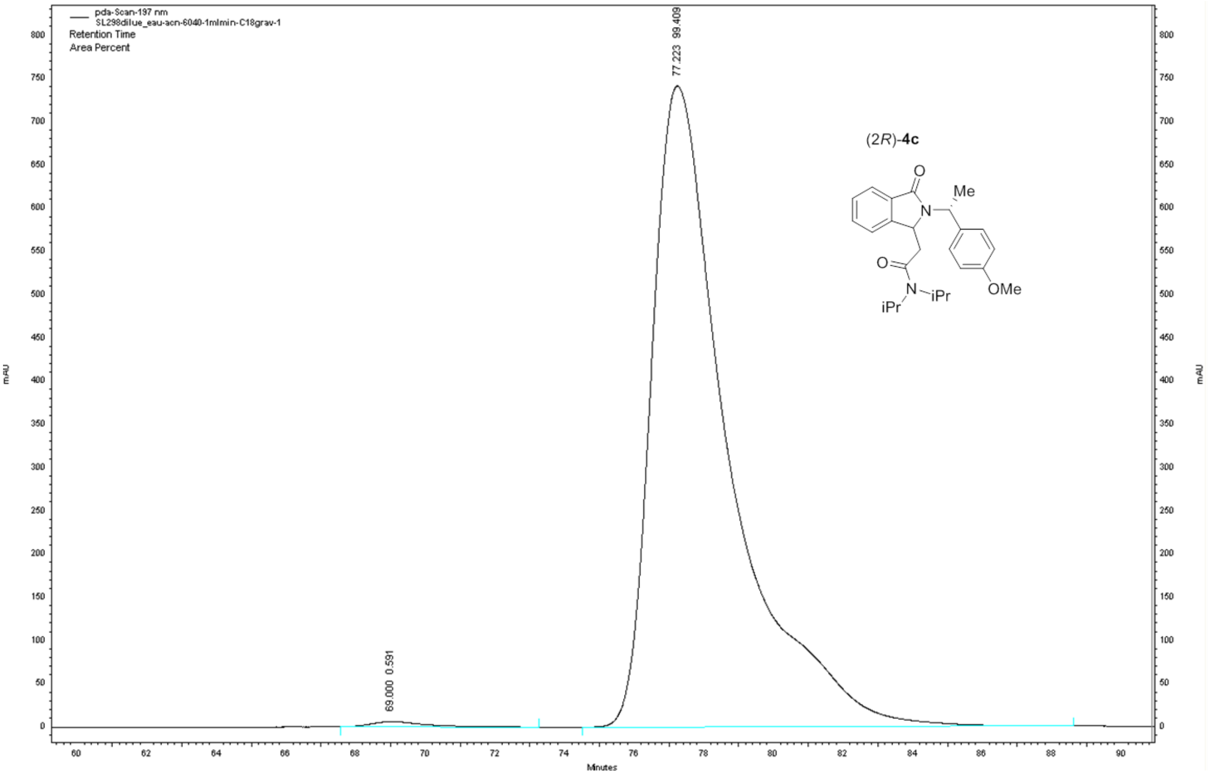
pda-Scan-197 nm
SL298dilue_eau-acn-6040-1mlmin-C18grav-1
Retention Time
Area Percent
77.223 99.409
69.000 0.591
(2R)-4c
Minutes
mAU

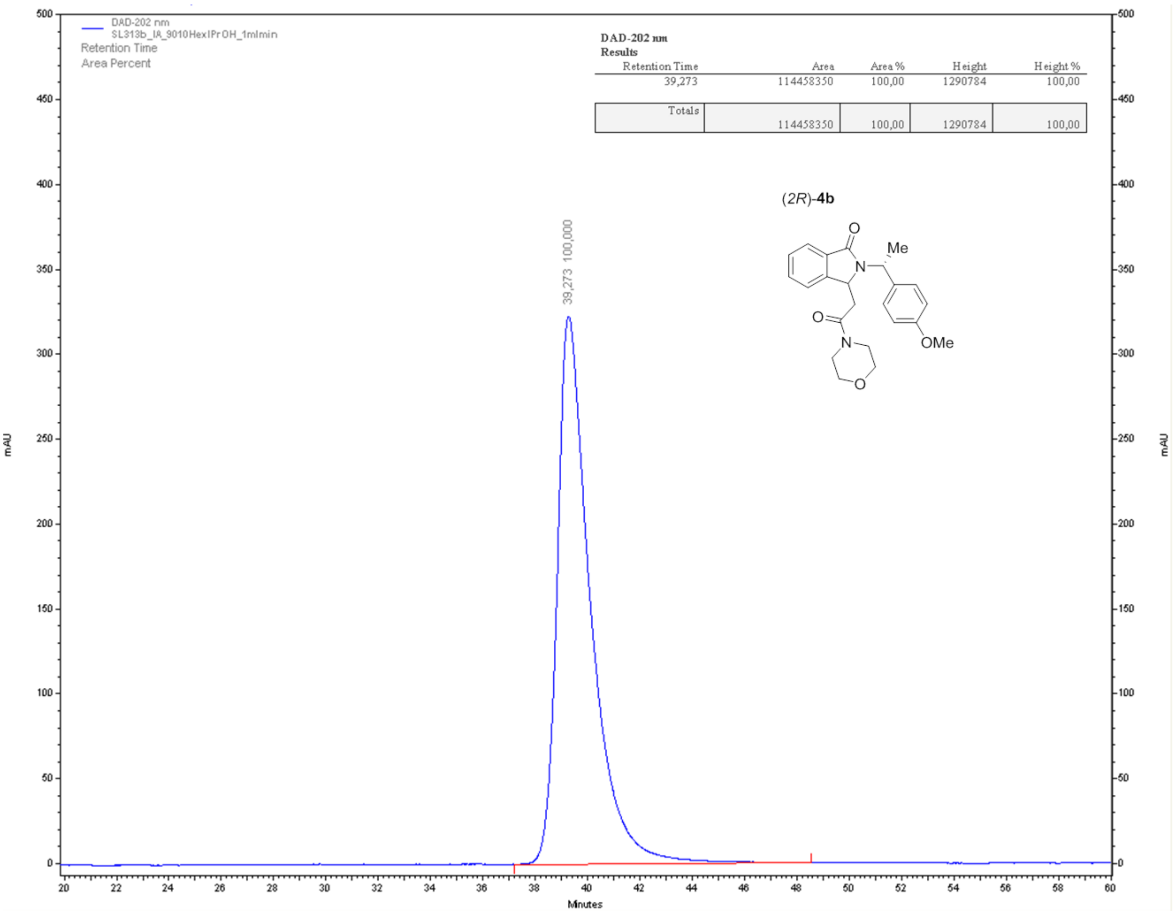
DAD-202 nm
SL313b_IA_9010HexIPrOH_1mlmin
Retention Time
Area Percent
DAD-202 nm
Results
Retention Time | Area | Area % | Height | Height %
39,273 | 114458350 | 100,00 | 1290784 | 100,00
Totals | 114458350 | 100,00 | 1290784 | 100,00
39,273 100,000
(2R)-4b
Me
OMe
mAU
Minutes

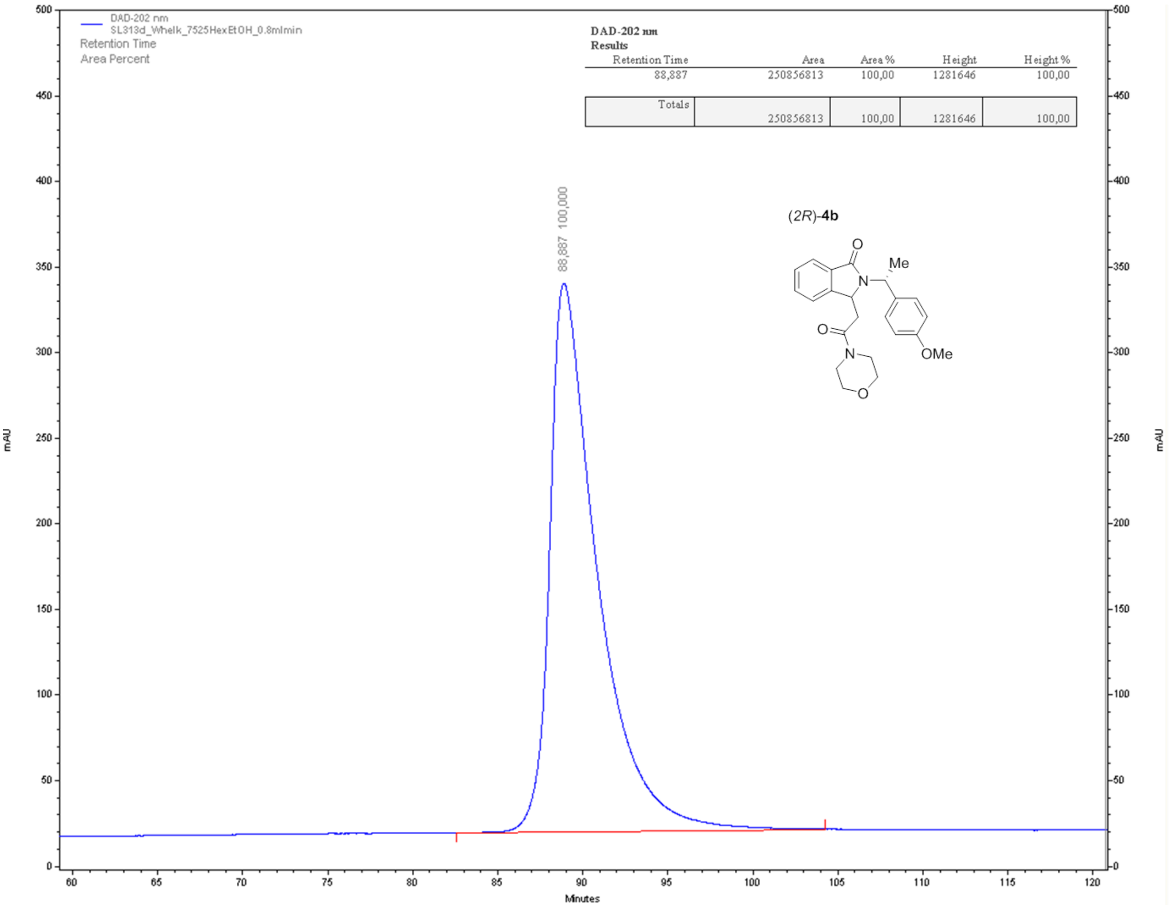
DAD-202 nm
SL313d_Whelk_7525HexEtOH_0.8mlmin
Retention Time
Area Percent
DAD-202 nm
Results
Retention Time | Area | Area % | Height | Height %
88,887 | 250856813 | 100,00 | 1281646 | 100,00
Totals | 250856813 | 100,00 | 1281646 | 100,00
88,887 100,000
(2R)-4b
Me
OMe
mAU
Minutes

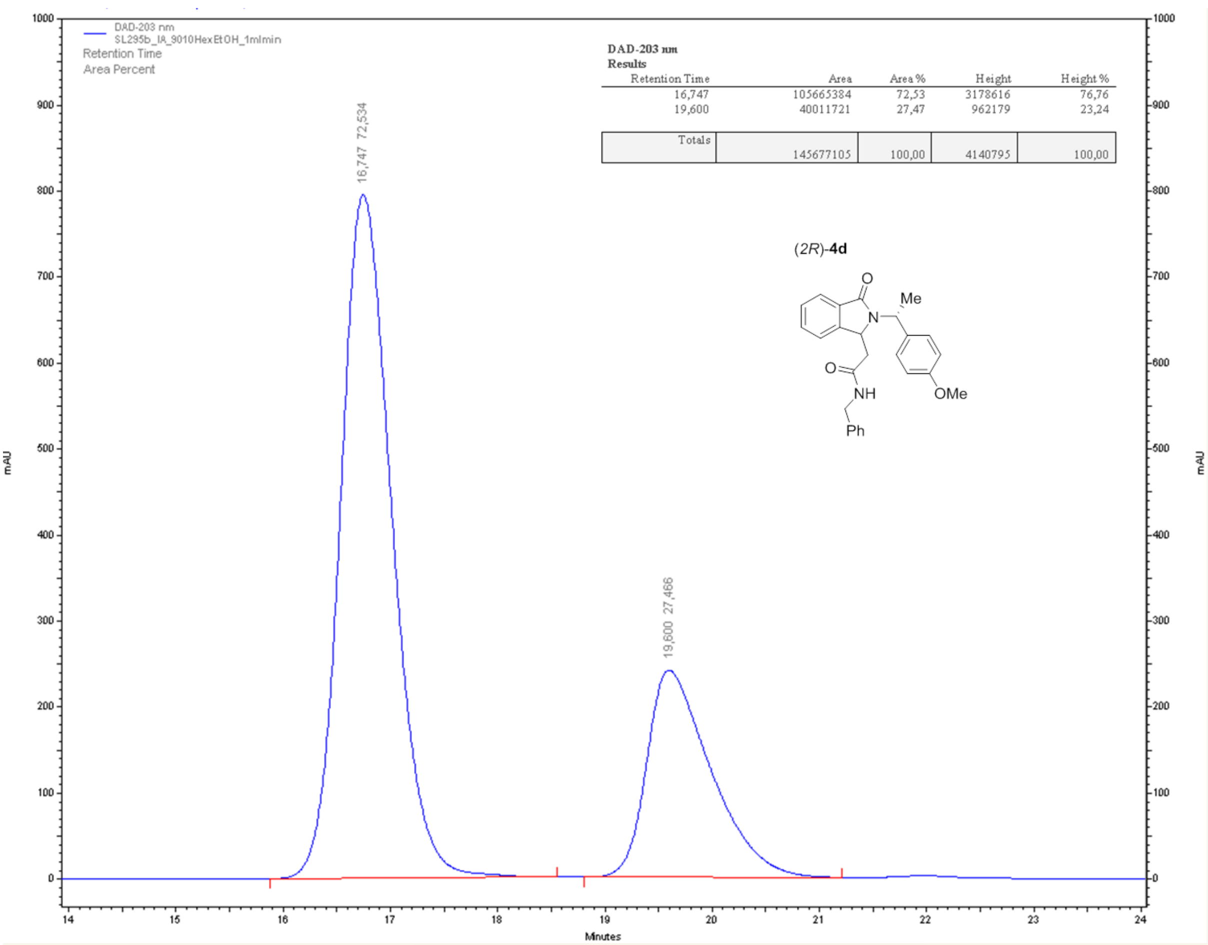

DAD-203 nm
Results

Retention Time	Area	Area %	Height	Height %
16,747	105665384	72,53	3178616	76,76
19,600	40011721	27,47	962179	23,24
Totals	145677105	100,00	4140795	100,00

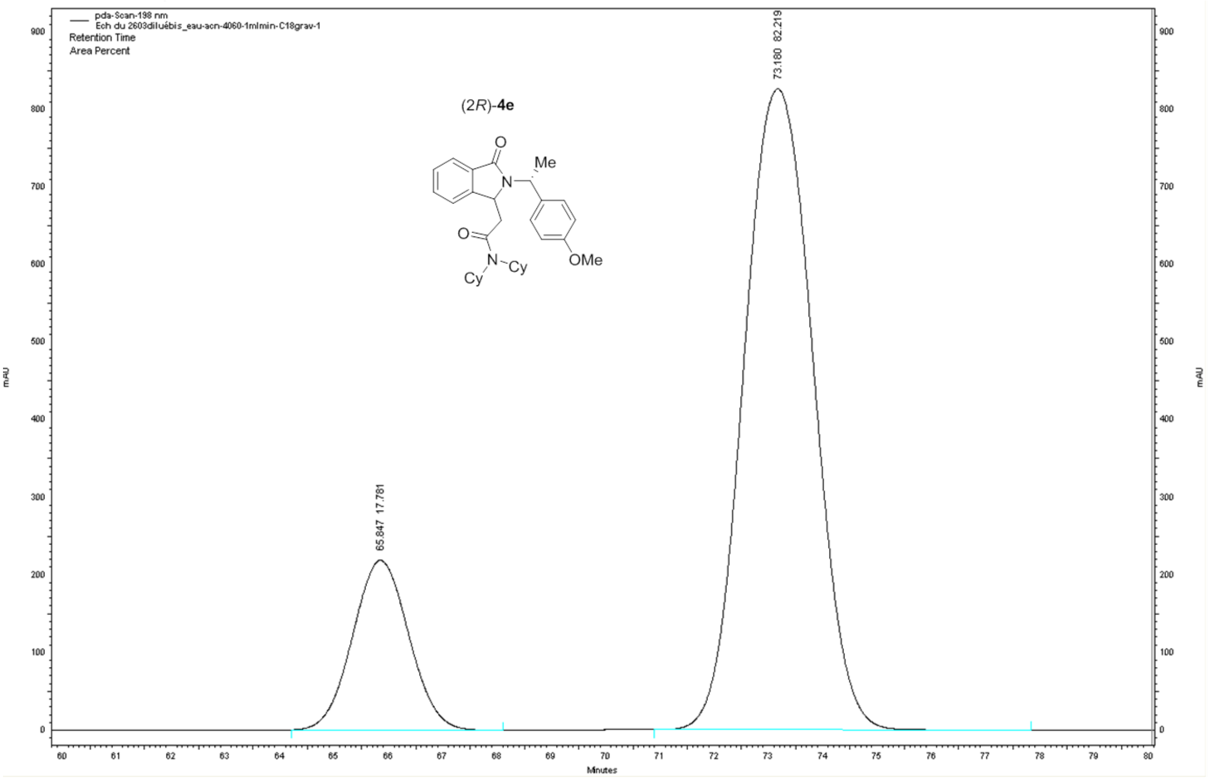

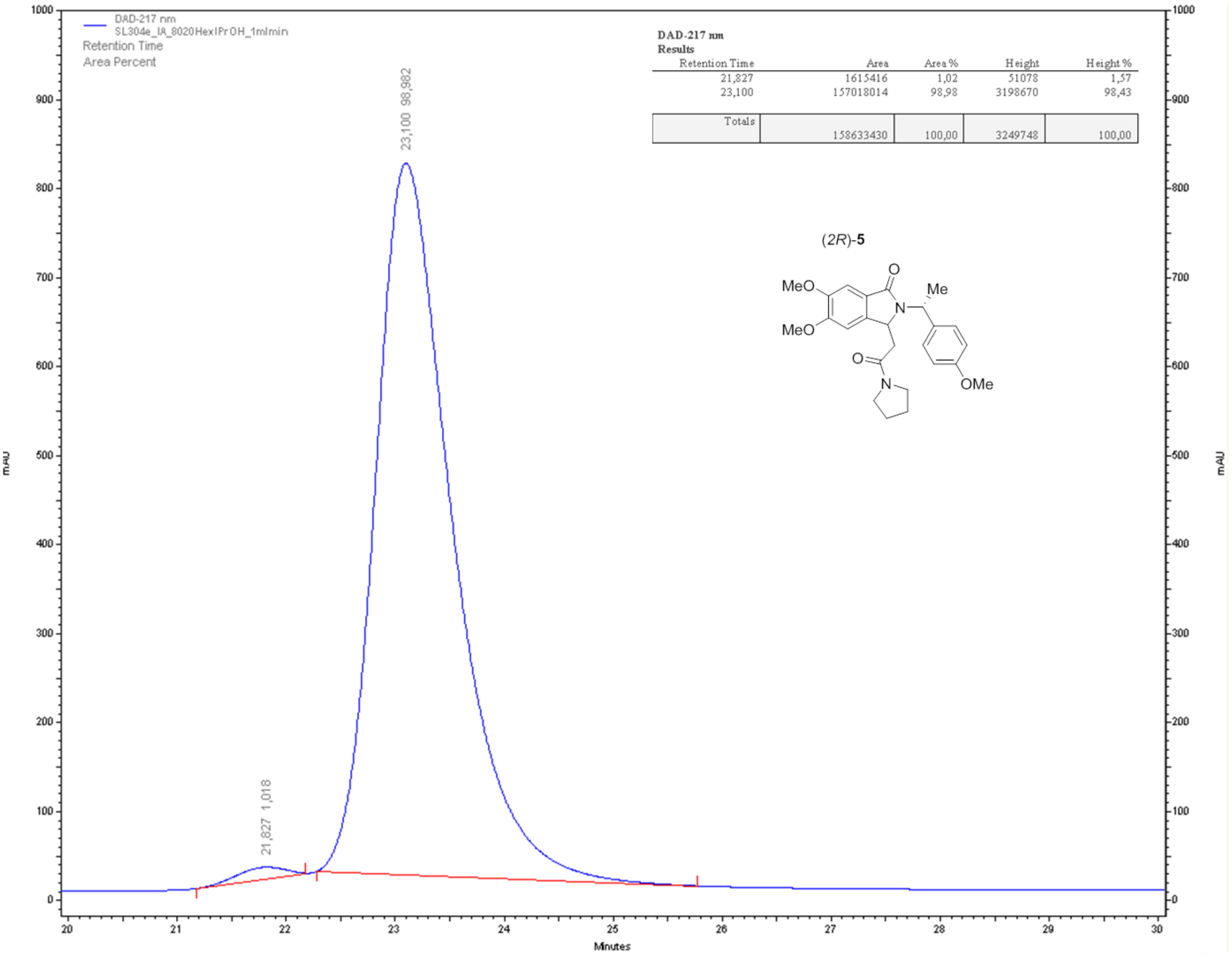
DAD-217 nm
SL304e_IA_8020HexIPrOH_1mlmin
Retention Time
Area Percent
23,100 98,982
21,827 1,018
DAD-217 nm
Results
Retention Time | Area | Area % | Height | Height %
21,827 | 1615416 | 1,02 | 51078 | 1,57
23,100 | 157018014 | 98,98 | 3198670 | 98,43
Totals | 158633430 | 100,00 | 3249748 | 100,00
(2R)-5
MeO
MeO
O
N
Me
O
N
OMe
mAU
Minutes

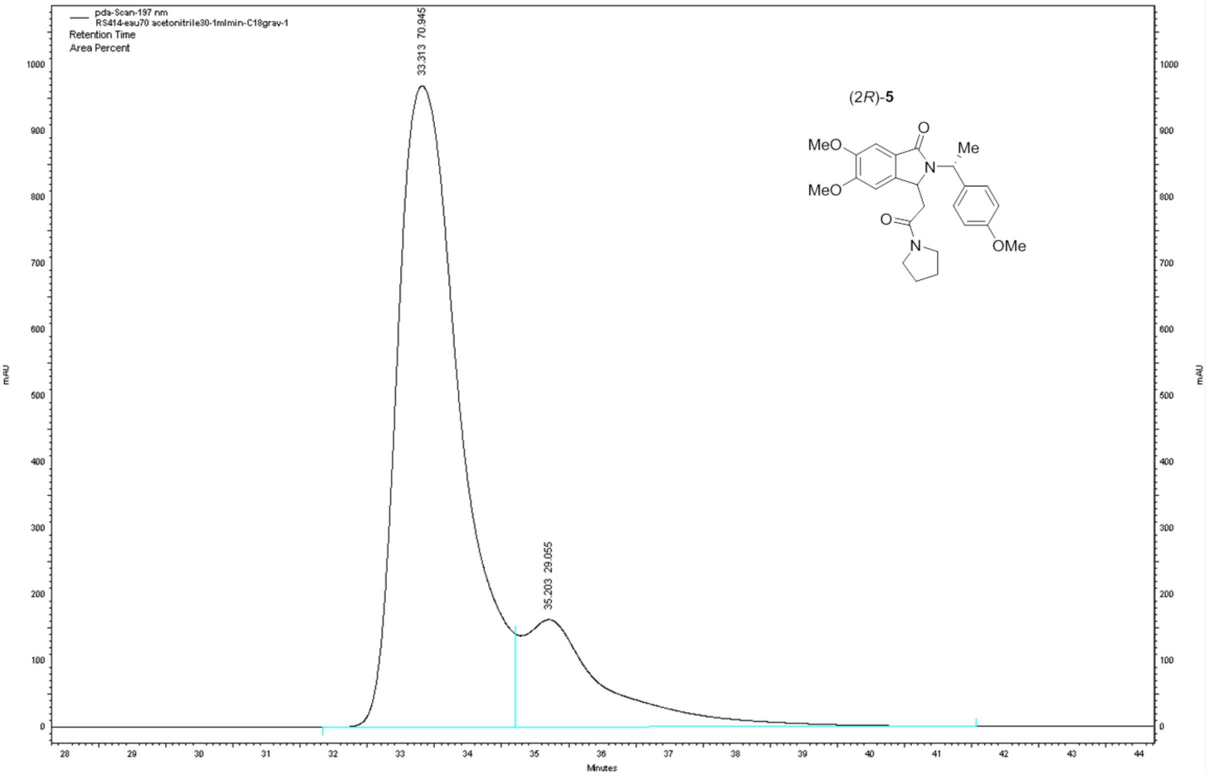
pda-Scan-197 nm
RS414-eau70 acetonitrile30-1mlmin-C18grav-1
Retention Time
Area Percent
33.313 70.945
35.203 29.055
(2R)-5
MeO
MeO
O
N
Me
O
N
OMe
mAU
Minutes

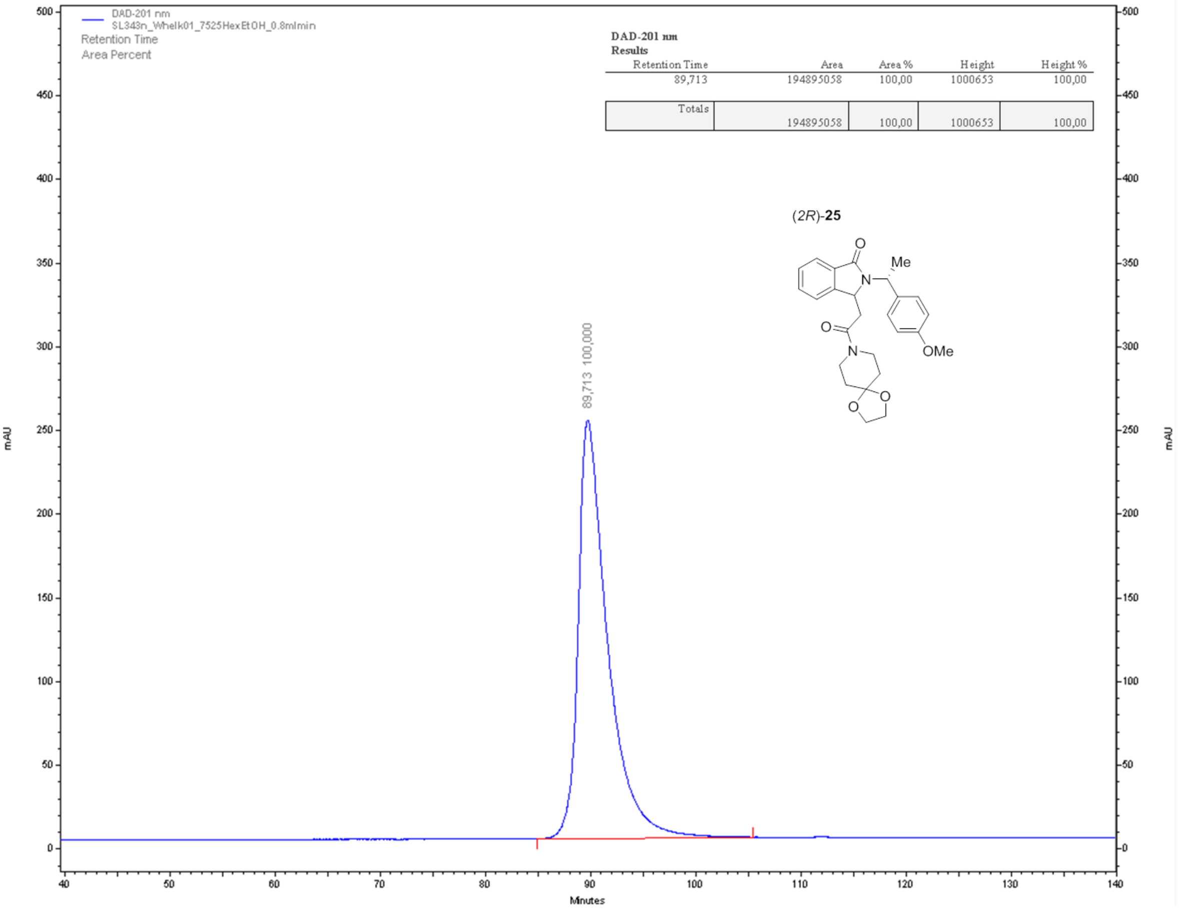

DAD-201 nm
Results

Retention Time	Area	Area %	Height	Height %
89,713	194895058	100,00	1000653	100,00
Totals	194895058	100,00	1000653	100,00

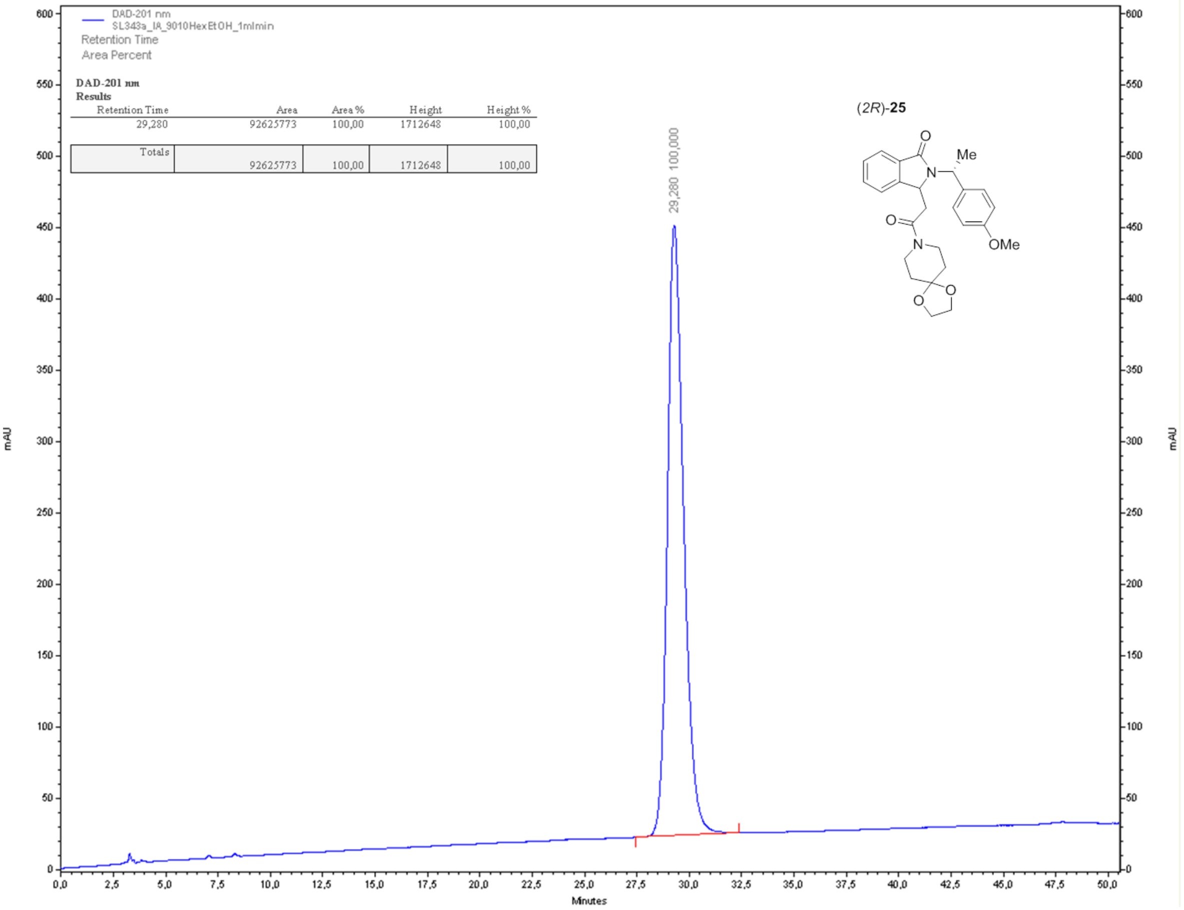

DAD-201 nm
Results

Retention Time	Area	Area %	Height	Height %
29,280	92625773	100,00	1712648	100,00
Totals	92625773	100,00	1712648	100,00

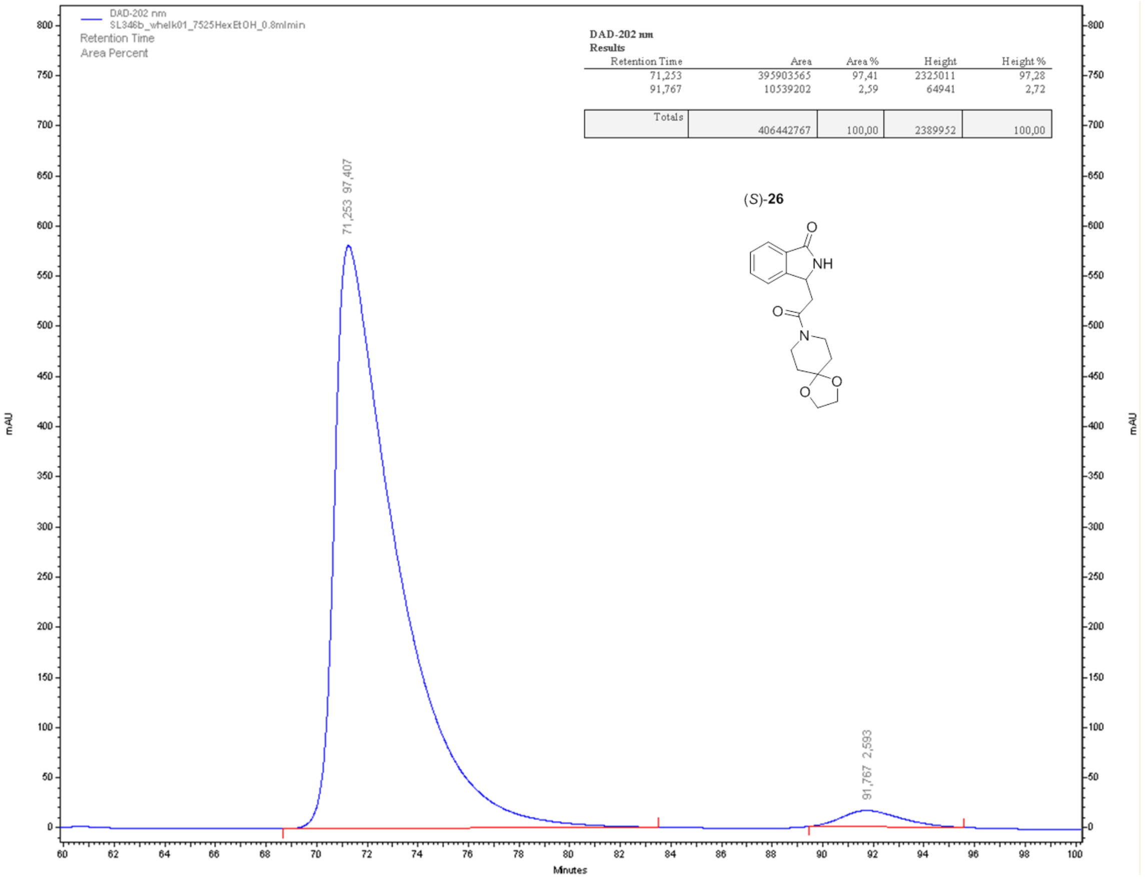

DAD-202 nm
Results

Retention Time	Area	Area %	Height	Height %
71,253	395903565	97,41	2325011	97,28
91,767	10539202	2,59	64941	2,72
Totals	406442767	100,00	2389952	100,00

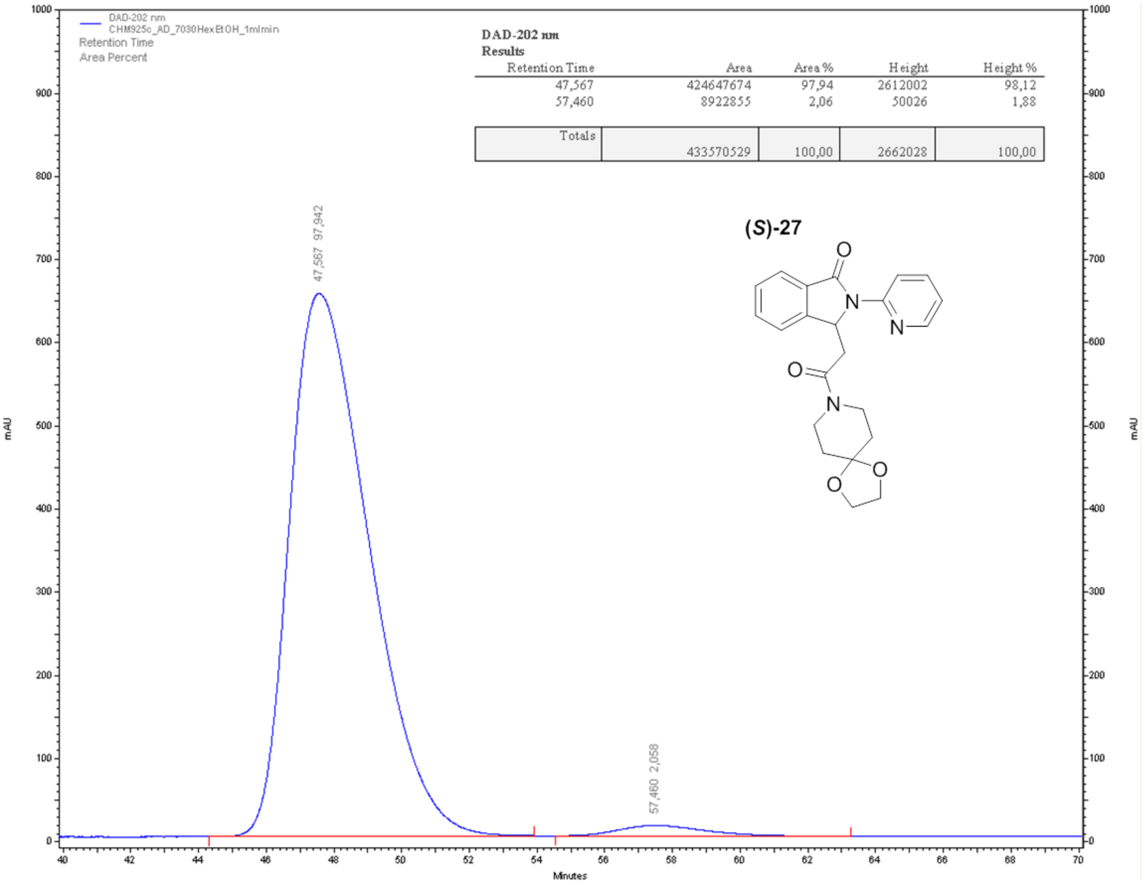

DAD-202 nm
Results

Retention Time	Area	Area %	Height	Height %
47,567	424647674	97,94	2612002	98,12
57,460	8922855	2,06	50026	1,88
Totals	433570529	100,00	2662028	100,00

6. X-ray analysis of compound 3a.

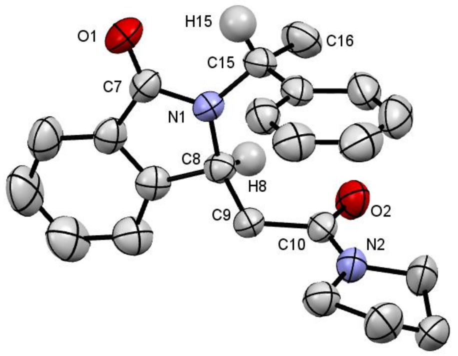

The (*S*) absolute configuration of the new stereogenic center of compound **3a** was confirmed by X-ray analysis (CCDC 1590565).

These data can be obtained free of charge from The Cambridge Crystallographic Data Centre via www.ccdc.cam.ac.uk/data_request/cif.

	Compound **3a** (CCDC 1590565)
formula	$C_{22}H_{24}N_2O_2$
mol. wt	348.43
cryst. Syst.	orthorhombic
Space group	*P 21 21 21*
a (Å)	10.4511(7)
b (Å)	12.4377(8)
c (Å)	14.3024(9)
α (deg)	90
β (deg)	90
γ (deg)	90
V (Å^3)	1859.1(2)
Z	4
colour	colourless
crystal dim. (mm)	0.32×0.23×0.08
D_{calc} (gcm^{-3})	1.245
F_{000}	744
μ (mm^{-1})	0.634
trans. Min. and max	0.5490/0.7528
T (K)	296.15
hkl limits	-12,+8/-11,+14/-16,+15
2θ limits (deg)	4.711/66.649
num. of data meas.	3130
num. of data with $I > 2$	2870
num. of var.	256
R	0.0361
R_w	0.0859
GOF	1.053
Flack	-0.19(15)
